# Selective autophagy of intracellular organelles: recent research advances

**DOI:** 10.7150/thno.49860

**Published:** 2021-01-01

**Authors:** Wen Li, Pengcheng He, Yuge Huang, Yi-Fang Li, Jiahong Lu, Min Li, Hiroshi Kurihara, Zhuo Luo, Tian Meng, Mashun Onishi, Changle Ma, Lei Jiang, Yongquan Hu, Qing Gong, Dongxing Zhu, Yiming Xu, Rong Liu, Lei Liu, Cong Yi, Yushan Zhu, Ningfang Ma, Koji Okamoto, Zhiping Xie, Jinbao Liu, Rong-Rong He, Du Feng

**Affiliations:** 1 Guangzhou Municipal and Guangdong Provincial Key Laboratory of Protein Modification and Degradation, State Key Laboratory of Respiratory Disease, School of Basic Medical Sciences, Guangzhou Medical University, Guangzhou 511436, China.; 2 International Cooperative Laboratory of Traditional Chinese Medicine Modernization and Innovative Drug Development of Chinese Ministry of Education (MOE), College of Pharmacy, Jinan University, Guangzhou 510632, China.; 3 Department of Pediatrics, The Affiliated Hospital of Guangdong Medical University, Zhanjiang, 524001, China.; 4 Department of Cardiology, Guangdong General Hospital's Nanhai Hospital, Foshan, China.; 5 Department of Cardiology, Guangdong Cardiovascular Institute, Guangdong Provincial Key Laboratory of Coronary Heart Disease Prevention, Guangdong General Hospital, Guangdong Academy of Medical Sciences, Guangzhou, China.; 6 State Key Laboratory of Quality Research in Chinese Medicine, Institute of Chinese Medical Sciences, University of Macau, Macau SAR, China.; 7 School of Pharmaceutical Sciences, Sun Yat-Sen University, Guangzhou, Guangdong, China.; 8 Affiliated Cancer Hospital of Guangzhou Medical University, Guangzhou 510095, China.; 9 Graduate School of Frontier Biosciences, Osaka University, 1-3 Yamadaoka, Suita, Osaka 565-0871, Japan.; 10 Shandong Provincial Key Laboratory of Plant Stress, College of Life Sciences, Shandong Normal University, Wenhua East Road 88, Jinan 250014, China.; 11 Department of Biochemistry and Molecular Biology, GMU-GIBH Joint School of Life Sciences, Guangzhou Medical University, Guangzhou 511436, China.; 12 Guangzhou Institute of Cardiovascular Diseases, The Second Affiliated Hospital, Key Laboratory of Cardiovascular Diseases, School of Basic Medical Sciences, Guangzhou Medical University, Guangzhou 511436, China.; 13 School of Basic Medical Sciences, The Sixth Affiliated Hospital of Guangzhou Medical University, Qingyuan People's Hospital, Guangzhou Medical University, Guangzhou, China.; 14 Department of Food Science, College of Food Science and Technology, Nanjing Agricultural University, Nanjing, Jiangsu 210095, China.; 15 National Center for International Research on Animal Gut Nutrition, Nanjing, Jiangsu 210095, China.; 16 State Key Laboratory of Membrane Biology, Institute of Zoology, Chinese Academy of Sciences; Beijing, China.; 17 Department of Biochemistry, and Department of Hepatobiliary and Pancreatic Surgery of the First Affiliated Hospital, Zhejiang University School of Medicine, Hangzhou 310058, China.; 18 State Key Laboratory of Medicinal Chemical Biology, College of Life Sciences, Nankai University, Tianjin 300071, China.; 19 State Key Laboratory of Microbial Metabolism & Joint International Research Laboratory of Metabolic & Developmental Sciences, School of Life Sciences and Biotechnology, Shanghai Jiao Tong University, Shanghai, China.

**Keywords:** selective autophagy, autophagy receptor, mitophagy, ER-phagy, proteaphagy, ribophagy, pexophagy, lipophagy, lysophagy, nucleophagy

## Abstract

Macroautophagy (hereafter called autophagy) is a highly conserved physiological process that degrades over-abundant or damaged organelles, large protein aggregates and invading pathogens via the lysosomal system (the vacuole in plants and yeast). Autophagy is generally induced by stress, such as oxygen-, energy- or amino acid-deprivation, irradiation, drugs, *etc*. In addition to non-selective bulk degradation, autophagy also occurs in a selective manner, recycling specific organelles, such as mitochondria, peroxisomes, ribosomes, endoplasmic reticulum (ER), lysosomes, nuclei, proteasomes and lipid droplets (LDs). This capability makes selective autophagy a major process in maintaining cellular homeostasis. The dysfunction of selective autophagy is implicated in neurodegenerative diseases (NDDs), tumorigenesis, metabolic disorders, heart failure, *etc*. Considering the importance of selective autophagy in cell biology, we systemically review the recent advances in our understanding of this process and its regulatory mechanisms. We emphasize the 'cargo-ligand-receptor' model in selective autophagy for specific organelles or cellular components in yeast and mammals, with a focus on mitophagy and ER-phagy, which are finely described as types of selective autophagy. Additionally, we highlight unanswered questions in the field, helping readers focus on the research blind spots that need to be broken.

## Introduction

The word *autophagy* is derived from the Greek roots “auto” (self) and “phagy” (eating). Autophagy can be divided into three main categories according to the different ways that the cellular contents are incorporated into the lysosome: microautophagy, chaperone-mediated autophagy (CMA), and macroautophagy 1-3. Neither microautophagy nor CMA requires the involvement of autophagosomes, but rather depends on the degradation function of lysosomes/vacuoles. Macroautophagy, usually referred to simply as autophagy, is a highly conserved process in which cellular contents are delivered by double-membrane vesicles, called autophagosomes, to the lysosomes for destruction 4. Autophagy is regulated by a series of autophagy related genes (ATGs). The process of autophagy can be divided into four steps. (1) Induction of autophagy: the Atg1/ULK1-containing complex is involved in this process. (2) Autophagosome formation: the class III PI3K-Atg14 complex, the Atg9-Atg2-WIPI-1 (Atg18) complex and the Atg5-Atg12-Atg16L1 complex are involved in autophagosome expansion and maturation, and lipidation of LC3/ GABARAP (Atg8 in yeast) is also required, which depends on ATG3, ATG4 and ATG7 5, 6. (3) Transport and fusion of autophagosomes with lysosomes: this process requires Rab7 7, 8, ectopic P-granules autophagy protein 5 homolog (EPG5) 8, 9, HOPS 10, 11, pleckstrin homology domain containing protein family member 1 (PLEKHM1) 12 and soluble NSF attachment protein receptors (SNAREs) 13, 14. (4) Completion of autophagy: this requires the degradation of the autophagosomal cargo by hydrolases and release of the degradation products into the cytosol by transporters/permeases, where they can be re-used to synthesize biomacromolecules or in other metabolic pathways 15, 16.

Autophagy can be either non-selective or selective. In selective autophagy, autophagy receptors bind to cargoes and result in degradation within lysosomes/vacuoles, depending on the core autophagy machinery. The typical characteristic of selective autophagy receptors is that they contain an Atg8-interacting motif (AIM)/LC3-interacting region (LIR) 17-19. The AIMs or LIRs provide selective binding to Atg8/LC3/GABARAP protein family. The AIMs or LIRs are generally characterized by sequences resembling [W/F/Y]XX[L/V/I], where X represents any amino acid. Isoleucine or leucine is typically observed as the third residue downstream of tryptophan 20. In addition to contribute to the biogenesis/maturation of autophasomes, the Atg8/LC3/GABARAP protein family also functions as a bridge between the cargo and the core autophagic machinery, ensuring efficient recognition and sequestration of the cargo within autophagosomes 21.

There are pathways for selective removal of mitochondria (mitophagy), proteasomes (proteaphagy), ribosomes (ribophagy), peroxisomes (pexophagy), ER (ER-phagy), lysosomes (lysophagy), LDs (lipophagy) and nuclei (nucleophagy) 22. Dysregulation of autophagy has a close relationship with several diseases, such as aging and neurodegeneration of the central nervous system 23, 24, cancer 25-27, metabolic disease 26, 28, heart dysfunction 29, 30 and inflammatory diseases 31, 32. The selective clearance of organelles or cellular components by autophagy is also critical for the homeostasis of eukaryote cells, protecting the cells from the potentially toxic byproducts and enabling regeneration of building blocks for synthesizing new organelle components.

**Figure 1:**
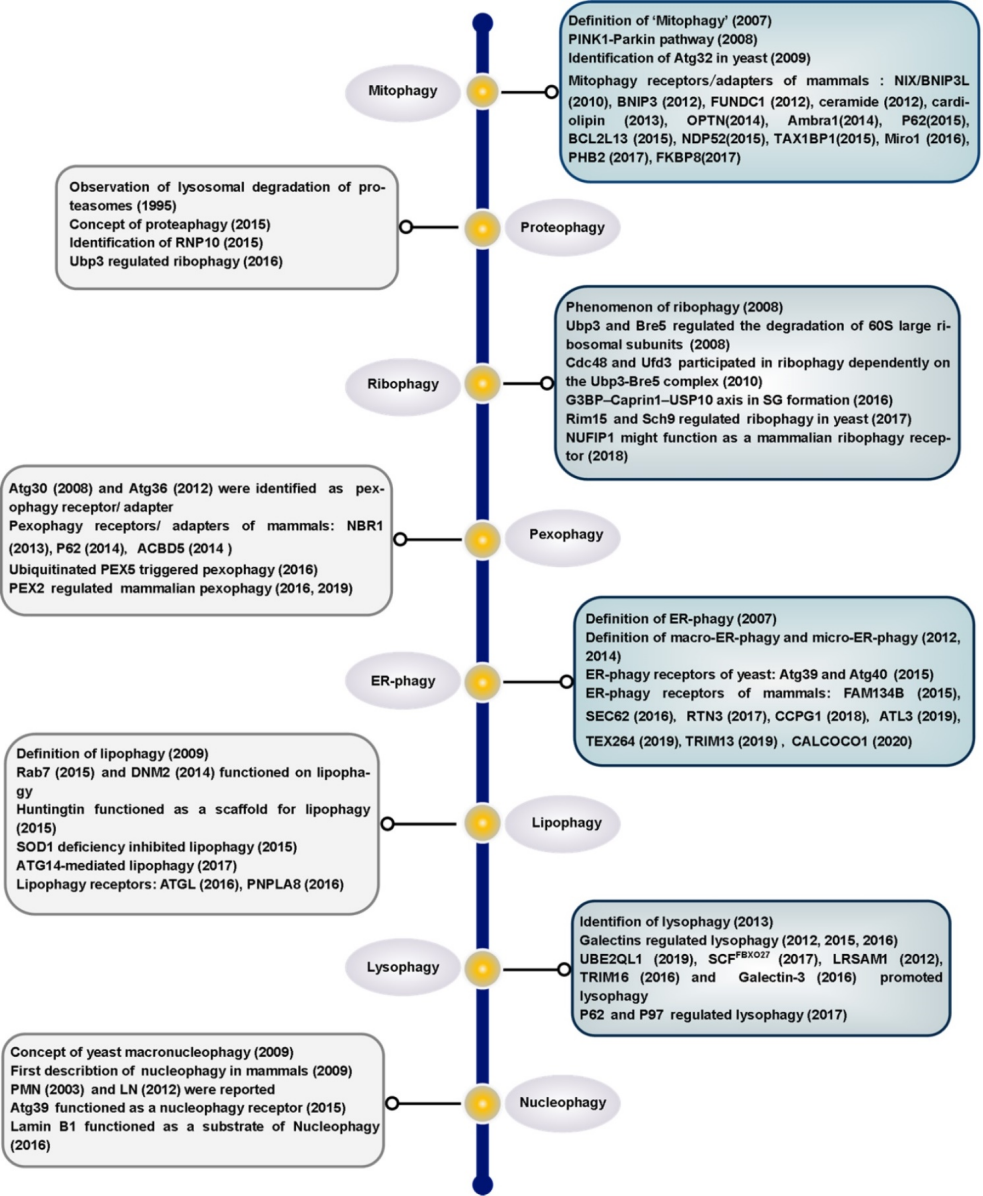
** Timeline of key discoveries in selective autophagy.** This timeline depicts a selection of important discoveries in different selective autophagy processes. However, not all important discoveries are included due to space limitations.

**Figure 2:**
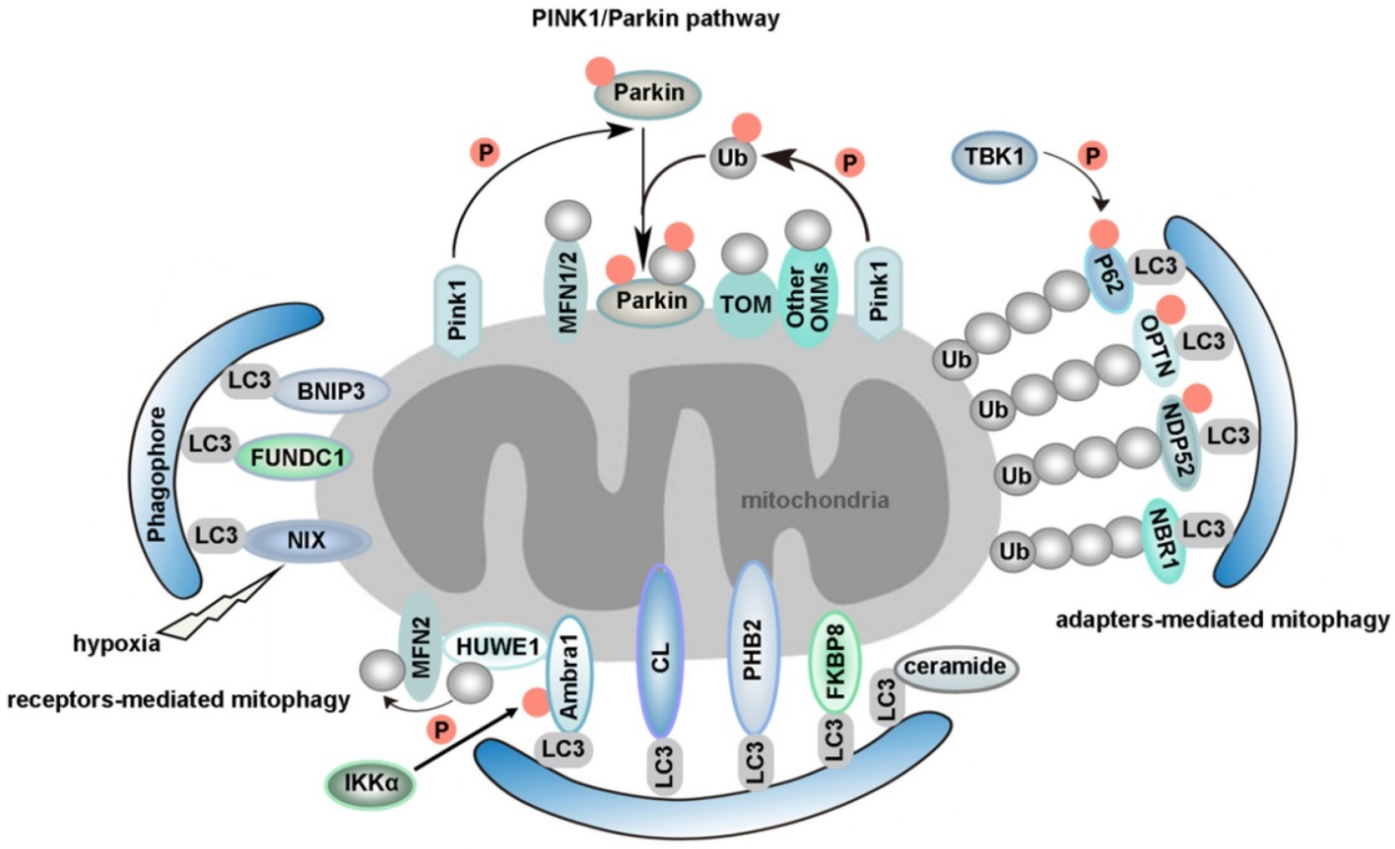
** Proposed models for different mammalian mitophagy pathways.** The diagram shows two classical mitophagy pathways: (1) mitophagy receptors/adapters-mediated mitophagy and (2) PINK1/Parkin-induced mitophagy. (1) Under hypoxic conditions, FUNDC1, BNIP3 and NIX recruits autophagosomes to mitochondria by direct interaction with LC3 through its LIR domains. Upon mitophagy induction, Ambra1 mediates HUWE1 translocation from cytosol to mitochondria, leading to the degradation of MFN2. This event is necessary for Ambra1-induced mitophagy. Additionally, the phosphorylation status of S1014 on Ambra1 by IKKα kinase enables the interaction between Ambra1 and LC3 during mitophagy. PHB2 is a newly identified inner mitochondrial protein that is crucial for targeting mitochondria for autophagic degradation. Externalization of CL to the OMM in response to mitochondrial damage serves as a recognition signal for selective autophagic clearance of dysfunctional mitochondria. CL interacts with LC3 and functions as a mitophagy receptor in cortical neurons of mammals. Ceramide has been identified as a selective receptor for mitophagy by binding directly to LC3. FKBP8, an OMM protein, is a novel mitophagy receptor, inducing the degradation of damaged mitochondria via the interaction with LC3. NDP52 and OPTN function as the bridge connecting UPS and autophagy, since they can bind both ubiquitin and LC3/GABARAP. NBR1, a functional homolog of P62, is dispensable for Parkin-mediated mitophagy regardless of the presence or absence of P62. TBK1-mediated phosphorylation promotes the recruitment of OPTN, NDP52, and P62 to depolarized mitochondria. (2) According to PINK1/Parkin-induced mitophagy, mitochondrial stress leads to mitochondrial damage, which is followed by PINK1-mediated translocation of Parkin from the cytosol to depolarized mitochondrion. Parkin then ubiquitinates outer mitochondrial membrane proteins, which further recruit P62 to the damaged mitochondrion and trigger selective mitophagy. Additionally, PINK1 becomes highly activated through cross-phosphorylation. Parkin and mitochondrial ubiquitin chains are phosphorylated by PINK1. The spatial conformation of phosphorylated Parkin is changed, which leads to the binding of phosphorylated Ub. After this stage, Parkin becomes fully active, and thus the ubiquitin-bound Parkin may transiently associate with mitochondria and interact with substrate proteins. This process compromises the integrity of the outer mitochondrial membrane, thus leading to mitophagy.

**Figure 3:**
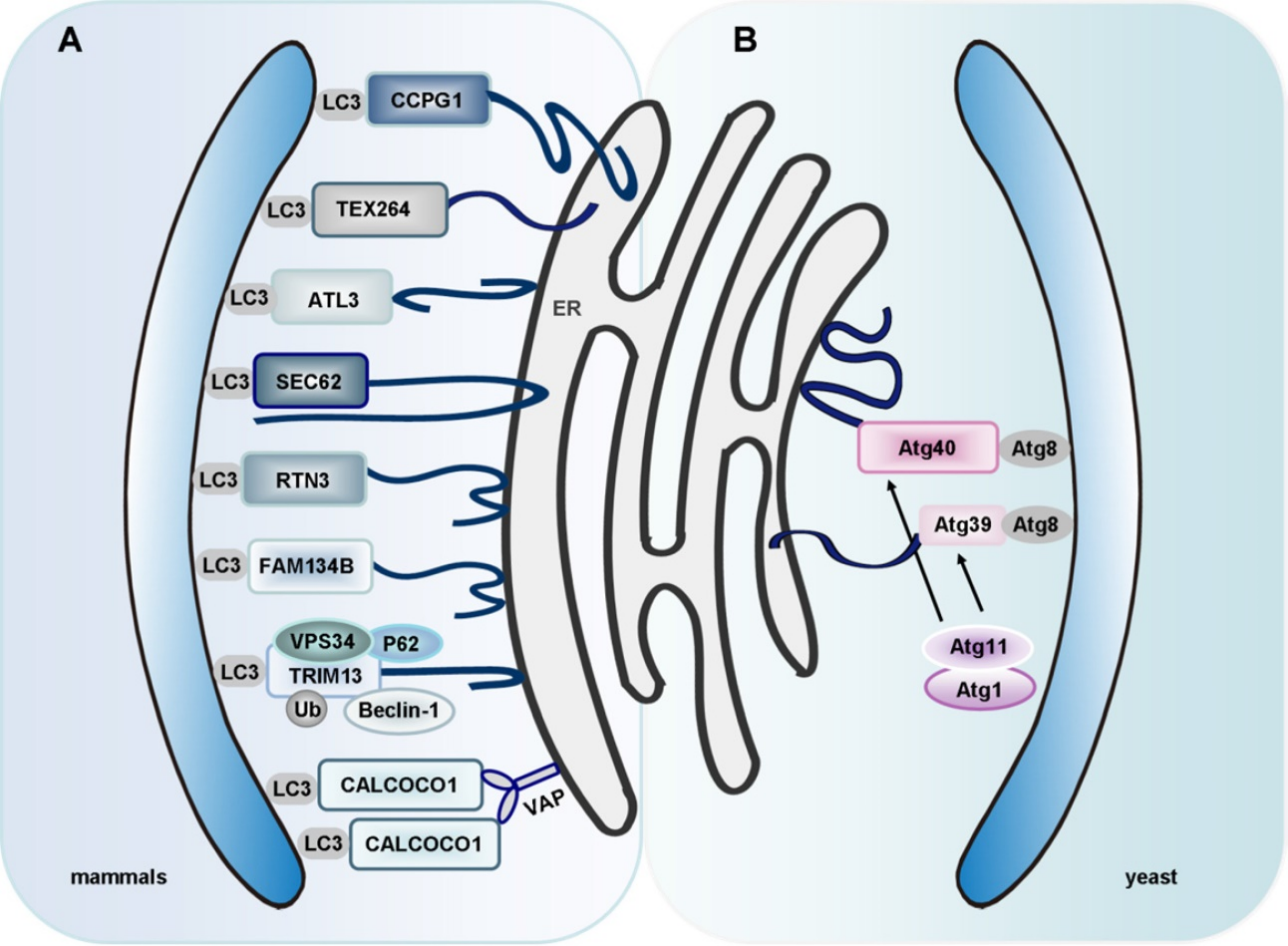
** ER-phagy: an important process for ER quality control. (A)** Eight mammalian ER-phagy receptors have been identified: FAM134B, SEC62, RTN3, CCPG1, ATL3, TEX264, TRIM13 and CALCOCO1. FAM134B, clustered at the edges of ER cisternae, binds to LC3 and GABARAP to facilitate ER-phagy. SEC62, an ER membrane protein, also works as an autophagy receptor. It is activated during the recovery from ER stresses to deliver selected portions of the ER to autolysosomes for clearance. RTN3, another specific receptor, is responsible for the degradation of ER tubules. CCPG1 directly binds to LC3 and FIP200 via a LIR motif and a FIP200-interacting region (FIR) motif, respectively. ATL3 is a receptor for selective turnover of tubular ER by autophagy upon starvation. It specifically binds to GABARAP. TEX264 is an ER-phagy receptor characterized by a single transmembrane domain and a LIR motif, and is the major contributor to ER-phagy in mammals. TRIM13 has been identified to be an ER-associated receptor of P62 in ER-phagy. The interaction between TRIM13, Beclin-1 and VPS34 is indispensable for ER membrane curvature and autophagosome biogenesis. Moreover, K63-linked Ub on TRIM13 promotes ER-phagy. CALCOCO1 directly binds to Atg8 proteins through LIR and UIR motifs. ER-phagy mediated by CALCOCO1 requires interaction with VAPs on the ER membrane. **(B)** Atg39 and Atg40 are ER-phagy receptors in yeast. Atg39 induces autophagic sequestration of part of the nucleus. However, Atg40 is enriched in the cortical and cytoplasmic ER, and induces these ER subdomains into autophagosomes.

**Figure 4:**
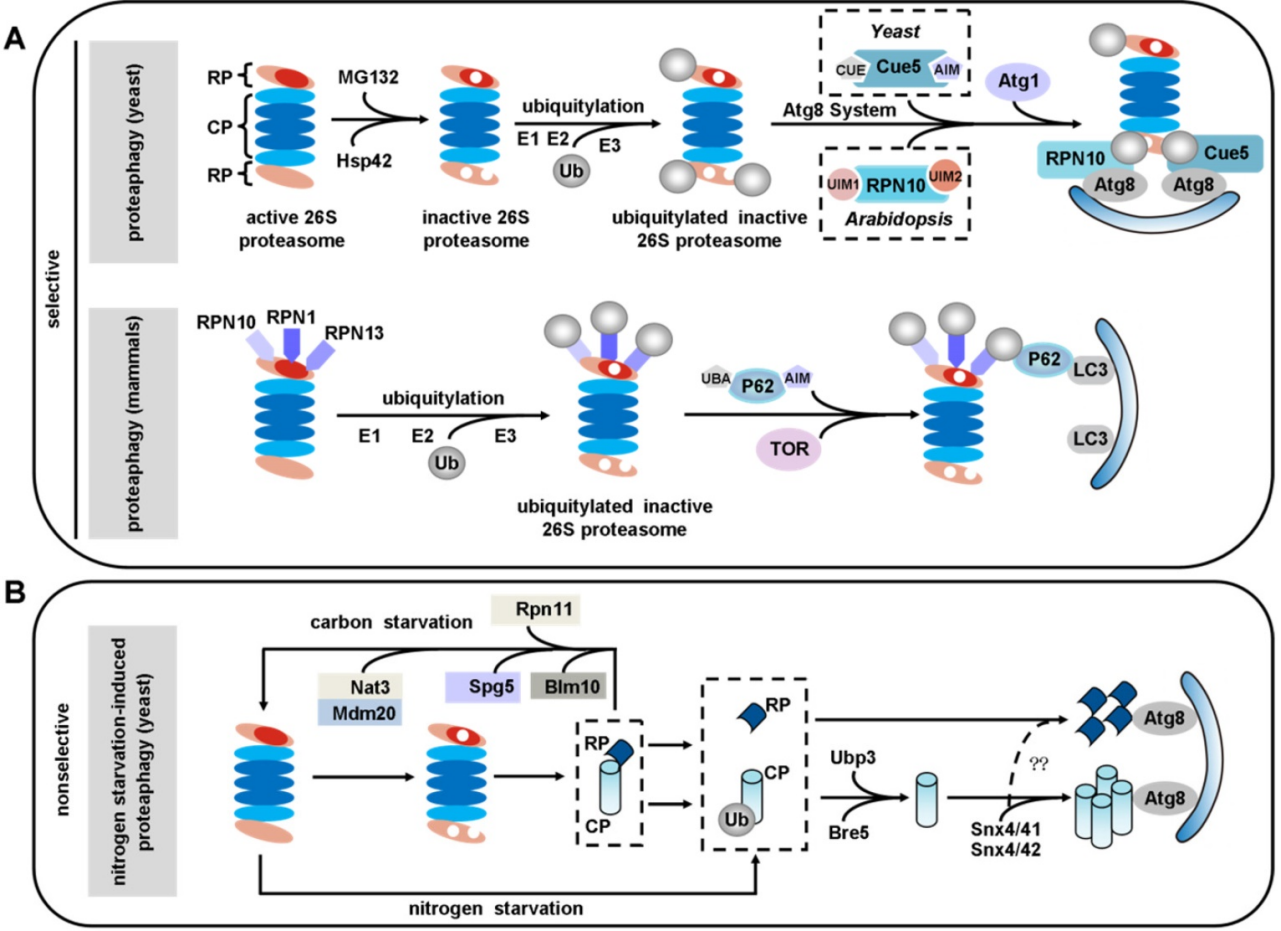
** Proteaphagy: selective autophagy of inactive proteasomes. (A)** Selective pathway: upon proteasome inactivation by MG132, 26S proteasomes are ubiquitinated in an Hsp42-dependent manner and shepherded to expanding autophagosome membranes by the selective proteaphagy receptors Cue5 in yeast or RPN10 in *Arabidopsis*. RPN10 recognizes Atg8, an ubiquitin-like modifier that decorates the autophagosome membrane, via the C-terminal ubiquitin-interacting motifs (UIMs) of RPN10. Cue5 can simultaneously bind ubiquitin and Atg8. In mammals, the proteasome subunits RPN1, RPN10, and RPN13 are poly-ubiquitinated upon amino acid starvation, which facilitates their recognition by P62. By simultaneous interaction with LC3, P62 delivers inactive 26S proteasomes to the expanding phagophore for eventual turnover by autophagy, a process that requires the TOR kinase. **(B)** Nonselective pathway: nitrogen starvation induces proteaphagy in yeast in a way that does not depend on RUNP10. During this process, 26S proteasomes dissociate into core particle (CP) and regulatory particle (RP) sub-complexes. The CP and RP then coalesce into cytoplasmic foci in a Snx4/41/42-dependent manner. Autophagy of CP (but not RP) also depends on the deubiquitinating enzyme Ubp3/Bre5. In contrast, during carbon starvation, proteasomes are reversibly sorted to avoid autophagic degradation. This process requires Blm10 for the CP, Spg5 and the C-terminus of Rpn11 for the RP, and the Nat3/Mdm20 complex for both.

**Figure 5:**
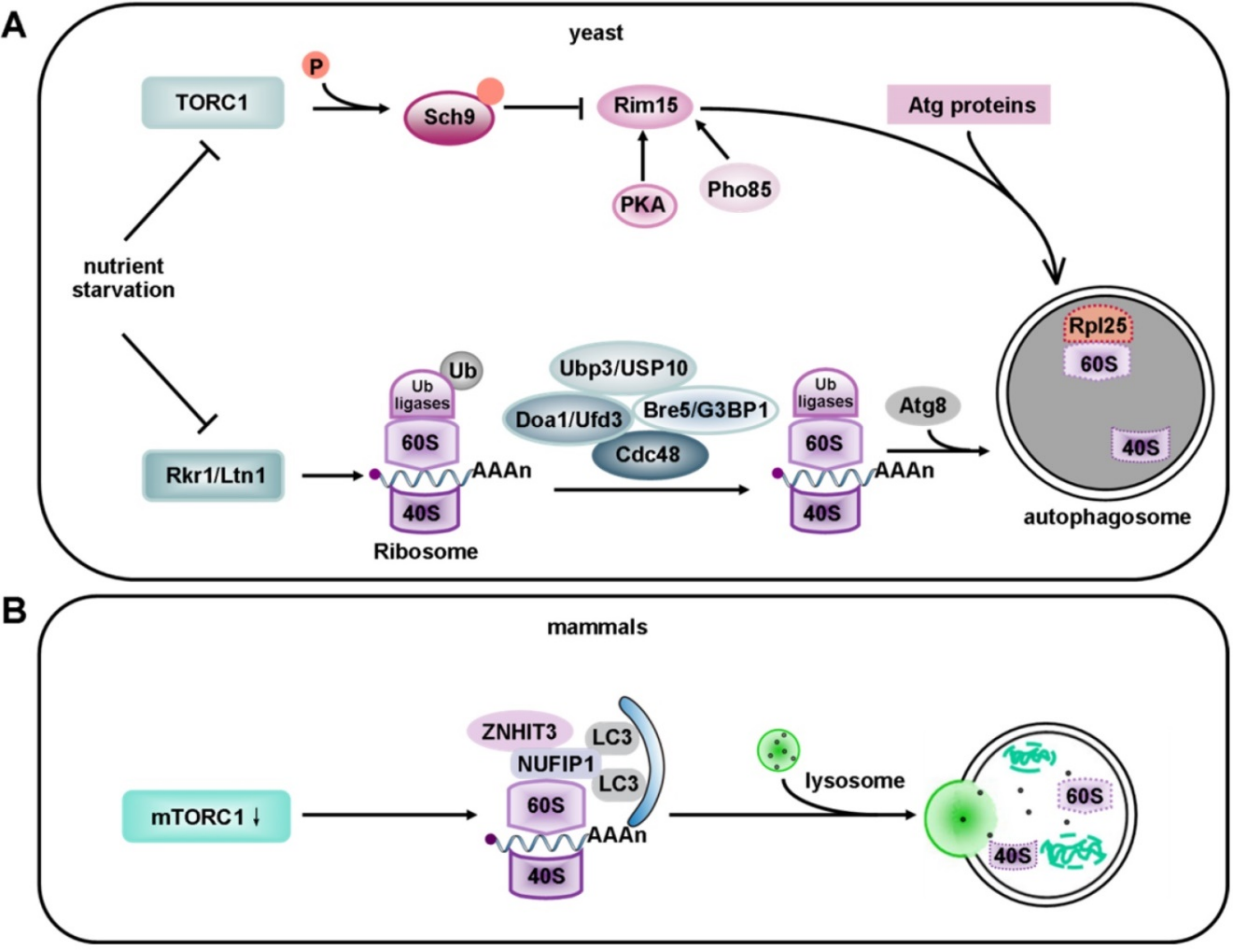
** Models of ribophagy in yeast and mammals. (A)** Ribosomes contain 40S and 60S ribosomal subunits that are selectively recruited to the phagophore. In yeast, upon the ubiquitination of 60S subunit, Rkr1/Ltn1 decreases during selective ribosome degradation under nutrient starvation. The mechanism for recruitment of 60S to the phagophore involves de-ubiquitination via the Ubp3-Bre5 complex, whereas the mechanism for 40S recruitment remains unclear. Recently, Cdc48 and Ufd3 were identified to be new partners of Ubp3. Cdc48 acts as a major factor of the ubiquitin and proteasome system, while Ufd3 functions as an ubiquitin-binding cofactor of Cdc48. Recently, the 60S ribosomal protein Rpl25 has been identified as a substrate of Ubp3 and Ltn1. Ubiquitylation of Rpl25 prevents 60S ribophagy. Upon starvation, Ubp3-mediated de-ubiquitination of Rpl25 accelerates the selective autophagy of 60S ribosomal subunits. However, in non-selective ribosome degradation, the expression of TORC1 is down-regulated after the onset of nutrient starvation, which leads to dephosphorylation of six residues in the C-terminus of Sch9. Sch9 negatively regulates Rim15 via phosphorylation. Rim15 is also regulated by protein kinase A (PKA) and Pho85. **(B)** In mammals, under nutrient-deprivation conditions, NUFIP1 and its binding partner ZNHIT3 redistribute from the nucleus to autophagosomes, lysosomes and ribosomes upon mTORC1 inhibition. NUFIP1 binds to LC3B and delivers ribosome to autolysosomes. Current evidence suggests that NUFIP1 is a mammalian ribophagy receptor.

**Figure 6:**
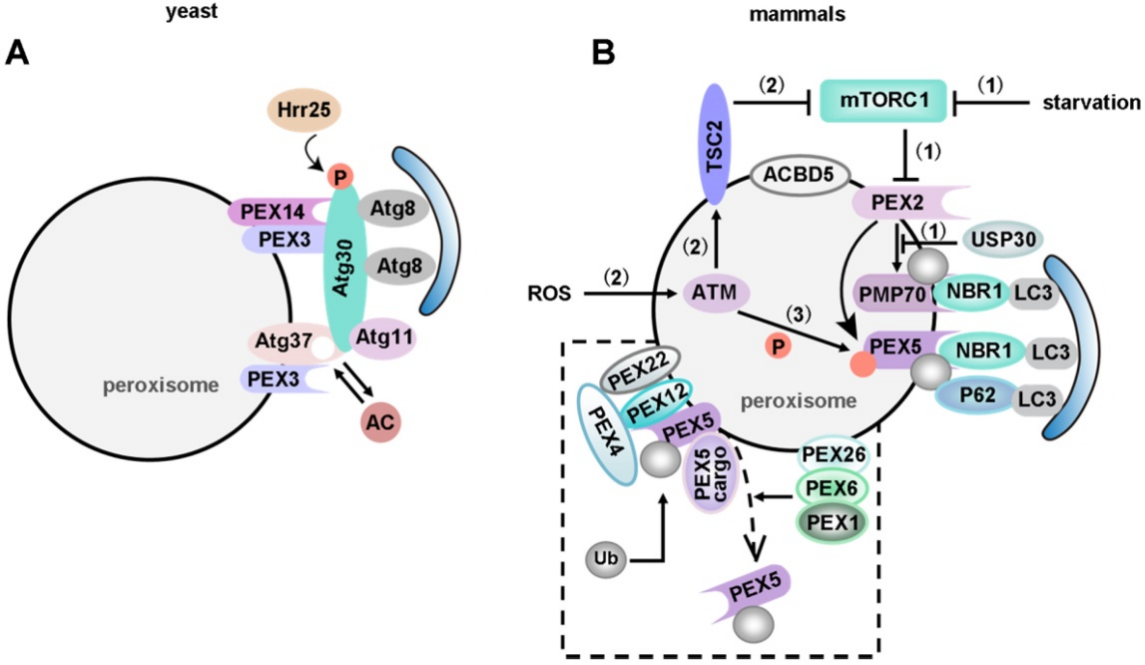
** Pexophagy in yeast and mammals. (A)** In yeast, Atg37 positively regulates the formation of RPC. Atg30 selectively degrades peroxisomes in a Pex3 and Atg37 dependent manner, by recruiting Atg8 and Atg11 to the RPC. Moreover, Atg30 is phosphorylated by Hrr25 kinase, and this phosphorylation can be regulated by Pex3 and Atg37, negatively and positively, respectively. Since Atg37 also functions as an Ac binding protein, Ac might regulate the Atg30-Atg37 interaction, hence affects the recruitment of Atg11 to the pexophagic RPC. **(B)** Pexophagy in mammals. Under normal conditions, a low PEX2 expression level is maintained via the mTORC1-mediated proteasome pathway. (1) Increased PEX2 during starvation conditions leads to the ubiquitination of PEX5 and PMP70, and ultimately induces pexophagy in an NBR1-dependent manner. On the other hand, USP30 counteracts PEX2 by deubiquitinating its substrates to prevent pexophagy. (2) ATM serine/threonine kinase is the first responder to peroxisomal ROS. The activated ATM kinase activates TSC2, and the activated TSC2 suppresses mTORC1. (3) ATM also phosphorylates PEX5 at Ser141, which triggers ubiquitination of PEX5 at Lys209. Ubiquitinated PEX5 is then bound by P62/NBR1 to induce pexophagy in response to ROS. The dashed box represents ubiquitin-dependent recognition of peroxisomes for pexophagy. The pexophagy target is PEX5 mono-ubiquitinated on Cys11. After cargo delivery, PEX1 and PEX6 (anchored on peroxisomes via PEX26) will remove ubiquitinated PEX5 from the peroxisomal membrane. ACBD5 is the only pexophagy-specific protein known to date. ACBD5 might be involved in the recruitment of pexophagy-specific receptors or adapters.

**Figure 7:**
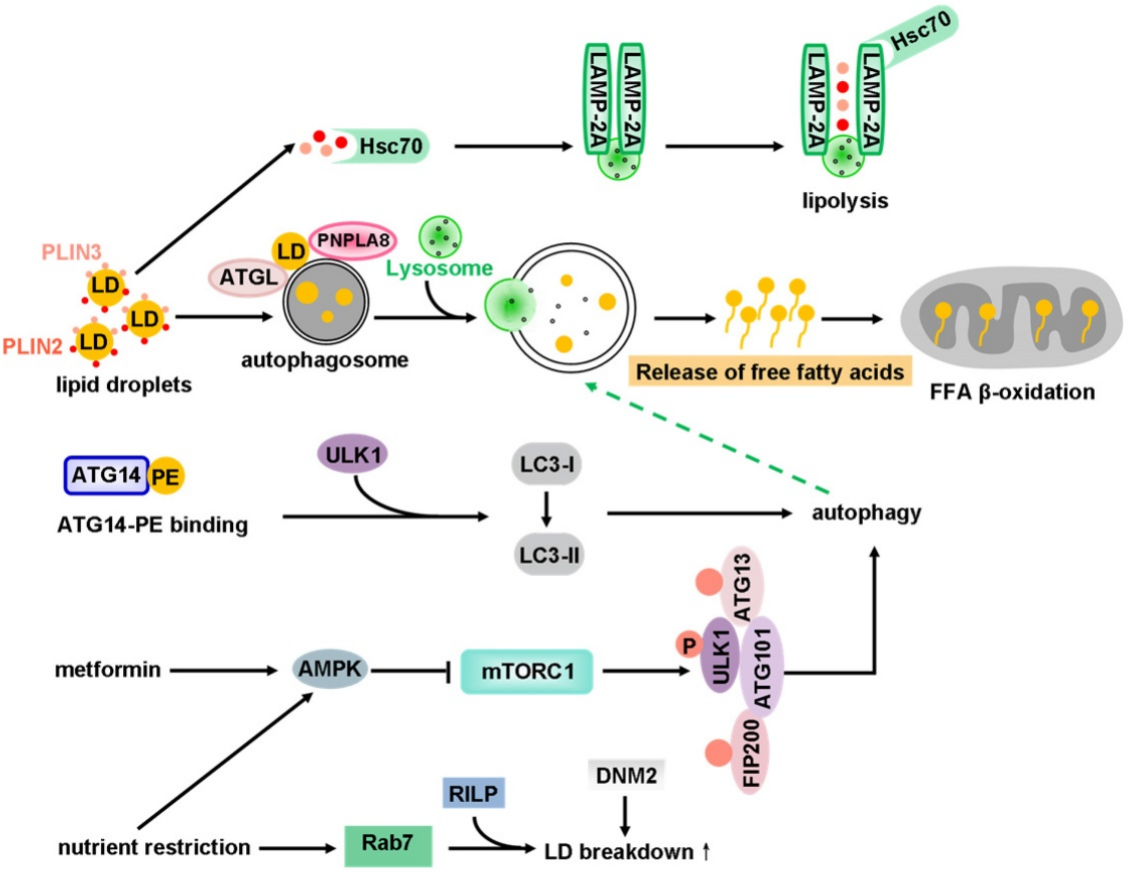
** Lipophagy: connecting autophagy and lipid metabolism.** Lipid droplets (LDs) are selectively removed by autophagy to generate free fatty acids (FFAs). The LD coat proteins PLIN2 and PLIN3 are degraded through the coordinated action of Hsc70 and the LAMP-2A receptor by CMA. ATGL and PNPLA8 function as selective autophagy receptors for lipophagy to promote LD catabolism and the oxidation of hydrolyzed FFAs. ATG14, which contains a PE-biding region, interacts with ULK1 and LC3 to induce lipophagy resulting in release of FFAs. The released FFAs continually undergo mitochondrial β-oxidation. ER stress induced by metformin or nutrient restriction activates the expression of AMPK. AMPK then inhibits mTORC1, which triggers autophagy by inducing the formation of the autophagy initiation complex (ULK1, ATG13, FIP200, ATG101). Upon nutrient deprivation, the expression of Rab7 (a small GTPase) increases. Rab7 promotes LDs breakdown via an interaction with its downstream effector RILP. DNM2, a large GTPase, regulates lipophagy at the level of autolysosome reformation due to its role in the budding of membrane tubules.

**Figure 8:**
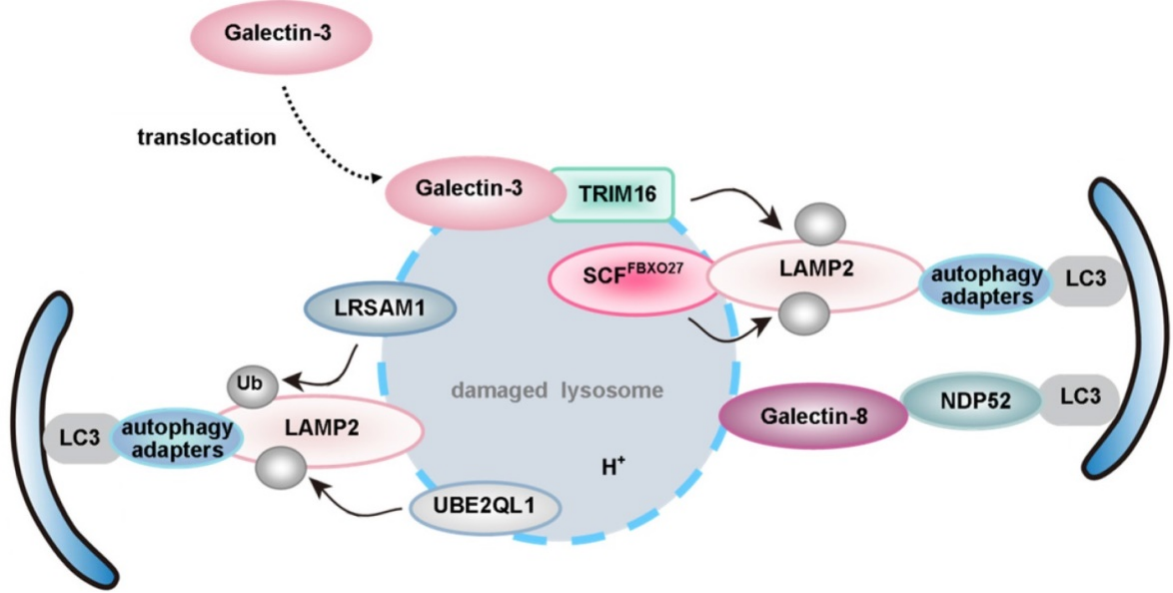
** Lysophagy: clearance of damaged lysosomes by autophagy.** When lysosomal membranes are damaged or even under normal conditions, lysophagy factors such as UBE2QL1, SCF^FBXO27^, LRSAM1 and TRIM16 are recruited to ubiquitinate lysosomal membrane proteins. Ubiquitinated proteins then recruit autophagy adapters (such as TAXBP1, SQSTM1, VCP and PLAA), leading to induction of lysophagy. Galectin-3 (which normally localizes in the cytoplasm and nucleus) can also be recruited to disrupted lysosomes, and the TRIM16-galectin-3 complex acts as a platform for assembly of autophagic initiation proteins that in turn induce phagophore formation. In contrast, galectin-8 directly binds the autophagic receptor NDP52 independently of ubiquitin, which recruits LC3-positive phagophores to mediate lysophagy.

**Figure 9:**
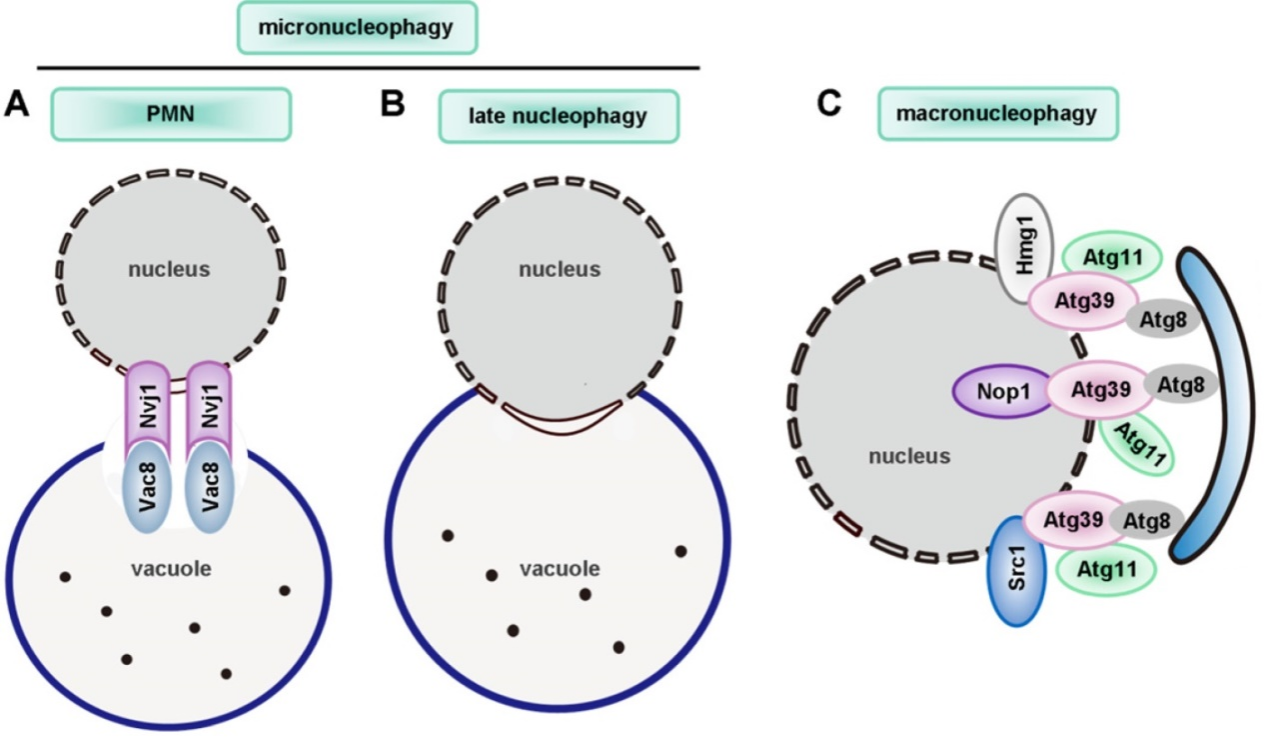
** Nucleophagy: selective degradation of genetic material.** Nucleophagy can be divided into macronucleophagy and micronucleophagy. Piecemeal micronucleophagy, also known as PMN, involves the direct engulfment of nuclear components by the vacuole, independent of autophagosome formation. **(A)** In yeast, PMN occurs under nutrient-rich and early nitrogen starvation conditions. PMN is characterized by the formation of nucleus-vacuole (NV) junctions involving Nvj1 and Vac8. The nuclear membrane protrudes toward the vacuole, and then becomes isolated from the nucleus and fuses with the vacuole for enzymatic degradation. **(B)** Under prolonged (>20 h) nitrogen starvation, late nucleophagy (LN) occurs without formation of NV junctions. **(C)** Macronucleophagy is the degradation of the nucleus and nuclear materials by auto-lysosomes (or vacuole in yeast). In yeast, the nucleophagy receptor Atg39 localizes to the nuclear envelope and interacts with Atg11 to cause the sequestration of nuclear envelope-derived double-membrane vesicles. Under various stresses, the nucleus and nuclear materials are degraded via macronucleophagy. The substrates of Atg39-dependent nucleophagy include the nuclear envelope proteins Hmg1 and Src1 and the nucleolar protein Nop1.

**Figure 10:**
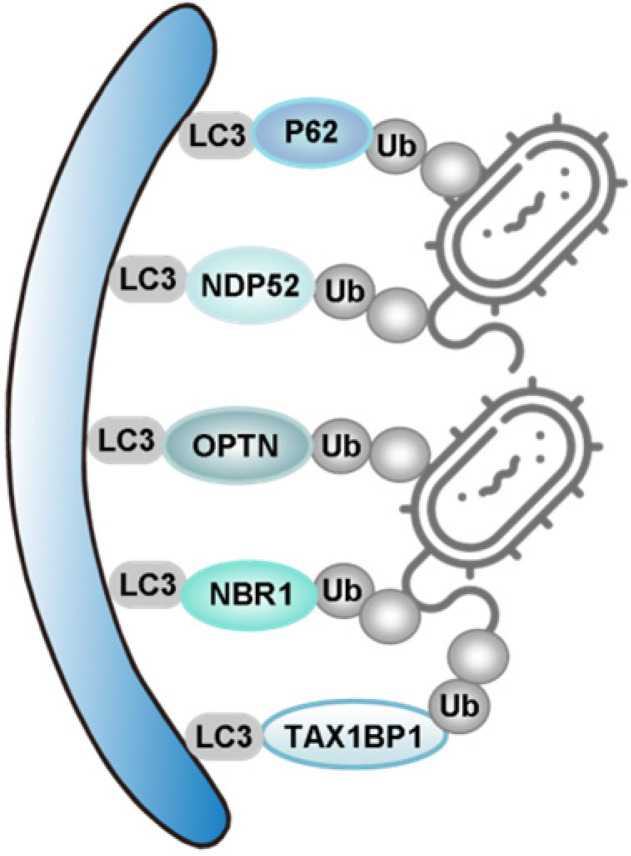
** Xenophagy: specialized elimination of intracellular pathogens.** Xenophagy utilizes autophagy receptors (P62, NDP52, OPTN, NBR1 and TAX1BP1) to selectively bring the ubiquitin-coated bacteria to autophagosome, through their interaction with LC3.

There have been many recent advances in the field of selective autophagy. We present the main discoveries and conceptual developments in the decades that have elapsed since the earliest definition of each individual selective autophagy process (**Figure 1**). Additionally, we review the literature on the clearance of specific organelles by autophagy and discuss how the 'cargo-ligand-receptor' model works in selective autophagy (**Figures 2-10**). Finally, multiple examples of receptor/adapter proteins will be showcased when we discuss different types of selective autophagy (**Table 1**).

## Mitophagy: quality control and clearance of mitochondria

Healthy mitochondria are the basis of energy production. In general, mitochondria maintain homeostasis through constant division, fusion, and mitochondrial autophagy, which are critical for maintaining the quality and quantity of mitochondria. Mitochondrial quality control occurs at three levels. First, there is regulation at the molecular level. This refers to the processes that repair or degrade misfolded or denatured proteins using the proteases and chaperones localized in mitochondria. Second, there is regulation at the organelle level. Mitochondrial repair can be achieved through fusion or fission. Insults that cause loss or degradation of proteins controlling mitochondrial fusion will likely trigger the division of mitochondria and induce mitophagy 33, 34. These signaling pathways from mitochondria to the nucleus include mitochondrial unfolded protein response (mtUPR), unfolded protein response activated by mistargeting of proteins (UPRam) and mitochondrial precursor over-accumulation stress (mPOS). The mtUPR triggers mitochondrial chaperone expression when mitochondrial protein folding is defective. The mPOS reduces protein translation and induces degradation of unimported proteins in the cytoplasm when mitochondrial import is impaired. A new mitochondrial quality control system, mitochondrial compromised import response (mitoCPR), was discovered in budding yeast. mitoCPR is triggered by protein import defects instead of by other mitochondrial defects, such as respiratory failure, and is mediated by the transcription factor pleiotropic drug resistance 3 (Pdr3). Pdr3 induces cistrinin resistance protein (Cis1, a peripheral outer membrane protein) expression when cells are exposed to mitochondrial protein import stress. Cis1 binds to the mitochondrial import receptor Tom70 and recruits Msp1 (an outer mitochondrial membrane protein) to mediate clearance and proteasomal degradation of unimported precursors from the mitochondrial surface. This plays a crucial role in maintaining mitochondrial function during mitochondrial import stress 35-38. Third, there is quality control at the cellular level. In the event of collapse of all the above mitochondrial quality control systems, cells will initiate the mitochondrial apoptotic program to remove damaged cells in some extreme cases 39.

Mitophagy is regarded as the central mechanism of mitochondrial quality control. The first mitophagy receptor, Atg32, was identified in yeast 40, 41. Several mitophagy receptors were then characterized in mammals, such as FUN14 domain containing 1 (FUNDC1) 42-44, NIX (also known as BNIP3L) 45, 46, Bcl-2/adenovirus E1B 19 kDa-interacting protein 3 (BNIP3) in the outer mitochondrial membrane (OMM) 47, 48, Prohibitin 2 (PHB2) in the inner mitochondrial membrane (IMM) 49, FKBP prolyl Isomerase 8 (FKBP8) 50, 51, optineurin (OPTN) 52, nuclear dot 52 kDa protein (NDP52) 53, P62 21, 53, neighbor of BRCA1 gene 1 (NBR1) 54, 55, activating molecule in Beclin1-regulatedautophagy (Ambra1) 56, tax1 binding protein 1 (TAX1BP1) 53, 57 and lipids such as cardiolipin (CL) 58, 59 and ceramide 60. We will review mitophagy from the following two perspectives: PINK1/Parkin-independent mitophagy and PINK1/Parkin-dependent mitophagy. Models for the major mammalian mitophagy pathways are illustrated in **Figure 2.**

### PINK1/Parkin-independent mitophagy

The typical characteristic of mitophagy receptors is that they have a LIR. In addition, phosphorylation is involved in regulating mitophagy. It has been previously demonstrated that AMP-activated kinase (AMPK) induces mitophagy through phosphorylation of unc-51 like autophagy activating kinase 1 (ULK1) under hypoxic conditions 61. However, the substrate and specific molecular mechanisms of ULK1 in the induction of mitophagy remain unknown. FUNDC1 is specific for hypoxia-induced mitophagy 42. Our previous study indicated that under hypoxic conditions, the expression of ULK1 increased and ULK1 translocated to mitochondria. On mitochondria, ULK1 interacted with FUNDC1, phosphorylating it at S17, which enhanced the binding of FUNDC1 to LC3. An ULK1-binding-deficient mutant of FUNDC1 prevents ULK1 translocation to mitochondria and inhibits mitophagy 62. It has been reported that casein kinase 2 (CK2) and Src phosphorylate FUNDC1 at S13 and Y18 to suppress mitophagy. Hypoxia also induces the release of phosphoglycerate mutase family member 5 (PGAM5), which enables the dephosphorylation of FUNDC1 at S13 to promote mitophagy 42. Conversely, ULK1 can be ubiquitinated by mitochondrial E3 ubiquitin protein ligase 1 (MUL1) after treatment of cells by selenite 62. BCL2 like 1 (BCL2L1) suppresses FUNDC1-mediated mitophagy via its inhibitory effect on PGAM5 63. A feedback mechanism to regulate FUNDC1 by the mitochondrial ubiquitin ligase membrane-associated RING-CH 5 (MARCH5) via ubiquitination desensitizes mitochondria to hypoxia-induced mitophagy 64. The bidirectional regulation of mitophagy ensures the quality control and dynamic equilibrium of mitochondria. We have shown that FUNDC1 is not only a novel mitochondrial-associated membrane (MAM) protein, enriched at the MAM by interacting with calnexin (CANX), but is also a new mitochondrial receptor for dynamin-related protein 1 (DRP1) to mediate mitochondrial fission in response to hypoxia 65, 66. In addition to interacting with DRP1, FUNDC1 can also interact with the inner mitochondrial membrane fusion factor optic atrophy type 1 (OPA1) to coordinate mitochondrial fission or fusion before mitophagy 44.

B-cell lymphoma 2 (BCL2), Bcl-2/adenovirus E1B 19-kDa-interacting protein 3 (BNIP3) and NIX belong to the BH3-only protein family. Although NIX is induced by hypoxia, it mainly functions in mitochondrial elimination in reticulocytes, which is essential for red blood cell maturation 46, 67, 68. NIX interacts with LC3 family proteins via its LIR motif to mediate autophagic clearance of mitochondria during reticulocyte maturation 45 (Figure 2). Interestingly, we have found that microRNA-137 is a novel hypoxia-responsive microRNA which inhibits mitophagy via regulation of both FUNDC1 and NIX 69. Similar to the post-translational modification of FUNDC1, the existing evidence indicates that phosphorylation of BNIP3 at S17 or S24 can enhance the BNIP3-LC3 interaction 48. BCL2-like 13 (BCL2L13), a functional mammalian homolog of yeast Atg32, can also induce mitophagy via its WXXI motif even in the absence of DRP1 and Parkin (also known as PARK2). In Atg32 knockout yeast, the expression of BCL2L13 rescued mitophagy 70. Moreover, the phosphorylation of serine residues in Atg32 and BCL2L13 is the key regulator of mitophagic activities. It has been revealed that phosphorylation of S114 of Atg32 is critical for the interaction between Atg32 and Atg11 71. S272 of BCL2L13 is also phosphorylated, and a BCL2L13 S272A mutant decreases the interaction with LC3 and the mitophagic activity of cells 70, 72. However, much work is needed to identify the kinase(s) for BCL2L13 S272. While BCL2L13 can induce mitophagy in a Parkin-independent way 73, the crosstalk between BCL2L13 and Parkin during mitophagy also deserves a better examination in the future.

Ambra1 is widely expressed in adult mouse brain 74. After autophagy stimulation, Ambra1 translocates from the cytoskeleton to ER and regulates autophagosome nucleation 75. Upon mitophagy induction, Ambra1 mediates HUWE1 (a novel E3 ubiquitin ligase) translocation from cytosol to mitochondria, favoring its binding to its substrate MFN2 and targeting it to the proteasome. This event is crucial and required for Ambra1-induced mitophagy. Moreover, the phosphorylation status of S1014 on Ambra1 by inhibitor of kappaB kinase alpha (IKKα) enables the interaction between Ambra1 and LC3 during mitophagy 76 (Figure 2). Interestingly, the pro-survival myeloid cell leukemia 1 (MCL1) may interfere with the recruitment of HUWE1 to mitochondria, thus inhibiting ubiquitylation of mitochondria, a critical event for Ambra1-mediated mitophagy. Mechanismly, glycogen synthase kinase-3 beta (GSK-3β) phosphorylates MCL1 on S159 and leads to the proteasomal degradation of MCL1 by HUWE1, therefore removing the inhibitory effect on mitophagy 77.

There are a few recently described but not well studied mitophagy receptors, such as PHB2 and FKBP8 (**Figure 2**). PHB2 is an inner mitochondrial protein that is crucial for targeting mitochondria for autophagic degradation. Wei *et al*. demonstrated that knockdown of PHB2 in HeLa cells prevented a decrease in the number of mitochondria following treatment with oligomycin and antimycin-A (OA), which indicated that PHB2 deficiency caused a defect in mitochondrial clearance. PHB2 is exposed and binds to LC3 via a canonical LIR, upon mitochondrial depolarization and Parkin- and proteasome-dependent outer mitochondrial rupture. In *C. elegans*, paternal mitochondria are selectively eliminated soon after fertilization by autophagy. Interestingly, mitochondrial DNA can still be detected in the subsequent generation after knockdown of PHB2 in males. In summary, PHB2 is required for both Parkin-mediated mitophagy in mammals and for paternal mitochondrial clearance in *C. elegans* embryos 49, 78, 79. CED-3 protease suppressor 6 (CPS-6), a mitochondrial endonuclease G, is critical for selective paternal mitochondrial elimination (PME) in *C. elegans*. CPS-6 translocates from the intermembrane space of paternal mitochondria to the matrix after fertilization to mediate the internal breakdown of paternal mitochondria and their enclosure by autophagosomes. Unlike mitophagy receptors, this protein acts with both the maternal autophagy and proteasome machineries to promote PME 80. Importantly, the PHB2-presenilin associated rhomboid like (PARL)-PGAM5-PINK1 axis is a novel pathway of PHB2-mediated mitophagy. Overexpression of PHB2 promotes Parkin recruitment to mitochondria. However, PHB2 depletion activates PARL (an IMM-resident protease) which cleaves PINK1 and PGAM5. Destabilized PINK1 blocks the mitochondrial recruitment of Parkin. Consequently, PHB2 depletion inhibits mitophagy 81.

FKBP8, an OMM protein, is a novel identified mitophagy receptor. Overexpression of FKBP8 induces mitochondrial fission. Mechanistically, FKBP8 acts with LC3 via the LIR motif to induce degradation of damaged mitochondria, while escaping degradation itself. Mutations in FKBP8 LIR lead to weakened binding of FKBP8 to Atg8 proteins 50, 51. Nevertheless, further investigation of FABS in mitophagy is needed. For instance, the possible interplay between FABS and other mitophagy receptors in mitochondrial clearance needs to be unraveled. We also wonder how FABS escapes from mitochondria to ER during mitophagy. How is FKBP8-LC3 binding normally regulated? Moreover, it will be important to elucidate how FABS is activated as a mitophagy receptor and whether there are post-translational modifications in FABS.

Whereas most selective autophagy receptors are proteins, some studies have shown that lipids can also function as mitophagy receptors. The unique dimeric phospholipid CL is located predominantly in the IMM. Owing to their metabolic features and post-mitotic state, neurons have evolved mechanisms to trigger externalization of CL to the OMM in response to mitochondrial damage, serving as a recognition signal for selective autophagic clearance of dysfunctional mitochondria. It has been revealed that CL interacts with LC3 and functions as a mitophagy receptor in cortical neurons of mammals. Blocking the synthesis of CL or inhibiting the translocation of CL might reduce mitophagy in these neurons 58, 59. An obvious question is if CL structurally mimics LIR domains in its binding to LC3, and if so how and why does this phenocopy arise? Given that CL interacts with LC3 and the key autophagy regulator Beclin-1, CL may also contribute to the formation of phagophores, which are autophagosome precursors 82. Interestingly, CL also interacts with other mitochondrial proteins such as voltage-dependent anion channel (VDAC), disrupting VDAC supramolecular assemblies 83. However, whether the effects of CL on VDAC gating functions may potentially affect mitophagy is still unknown. Moreover, CL was reported to be involved in regulating mitophagy in* Saccharomyces cerevisiae*. Loss of CL impairs activation of the Protein Kinase C (PKC) and High Osmolarity Glycerol (HOG) pathways, leading to defective mitophagy 84.

Additionally, ceramide, a bioactive sphingolipid, has been identified as a selective receptor for mitophagy by binding directly to the LC3-II protein 60. Nevertheless, how ceramide recruits the mitophagy machinery in relation to the function of CL remains to be determined. In addition, since DRP1 is required for ceramide-mediated mitophagy 85, further studies should be invested to clarify the relationship between ceramide and mitochondrial dynamics. Moreover, the mechanism underlying DRP-1 activation in ceramide-mediated mitophagy is still not clear. Studies looking at other bioactive sphingolipid molecules that can regulate mitophagy are also important for the field.

### PINK1/Parkin-dependent mitophagy

The PINK1/Parkin pathway is another important quality control system which participates in the selective clearance of unhealthy mitochondria 86, 87. In mammals, mitophagy can also be induced by respiratory inhibitors, respiratory uncouplers, mitochondrial reactive oxygen species (mtROS), or proteotoxicity, *etc*, via a Pink1/Parkin-dependent mechanism 88, 89. For instance, carbonyl cyanide m-chlorophenylhydrazone (CCCP), an uncoupler of the mitochondrial respiratory chain, can change the mitochondrial membrane potential, which leads to collapse of mitochondria. Parkin is a cytosolic E3 ubiquitin ligase, and PTEN-induced putative kinase 1 (PINK1) is an OMM kinase. Previous data indicates that the accumulation of PINK1 in the injured mitochondria can provide the signal for Parkin to selectively degrade mitochondria 90, 91. PINK1 activates Parkin *in vitro* by phosphorylating it at S65. In addition, PINK1 promotes the recruitment of Parkin from the cytosol to the OMM and enhances Parkin's E3 ligase activity 92-97. PINK1 becomes highly activated through cross-phosphorylation. A mechanistic study of mitophagy revealed that the phosphorylation of Ub by PINK1 is essential during the activation and translocation of Parkin from the cytoplasm to damaged mitochondria 98. After this stage, Parkin becomes fully active, and thus the ubiquitin-bound Parkin may transiently associate with mitochondria and interact with substrate proteins. In conclusion, the processes of Parkin activation and Parkin-induced mitophagy can be regarded as a feed-forward amplification loop which involves two steps: (1) during the initiation of activation, Parkin ubiquitinates a variety of substrates, including itself 99-103; and (2) the Ub conjugates attached to these substrates can in turn be phosphorylated by PINK1, followed by Parkin recruitment and activation. This process ultimately leads to polyubiquitination of multiple mitochondrial substrates. The newly formed Ub chains are phosphorylated by PINK1, which elicits further recruitment and activation of Parkin 95, 104 (**Figure 2**). It has been uncovered that sorting and assembly machinery (SAM) 50 is a significant regulator of mitochondrial dynamics and PINK1/Parkin-mediated mitophagy. Deprivation of Sam50 leads to PINK1 accumulation, Parkin recruitment, and mitophagy 105.

Targeting of ubiquitinated mitochondria to autophagosomes is a critical step for mitophagy 106. TAX1BP1 has been described as a bridge linking selective ubiquitinated cargoes to autophagosome components 107. It has been revealed that TAX1BP1 is required for PINK1/Parkin-mediated mitopahgy 103, 108. Overexpression of TAX1BP1 can promote mitophagy 53.

Ambra1 plays a role in both PINK1/Parkin-independent mitophagy and canonical PINK1/Parkin-dependent mitophagy 76, 109. The Ambra1-LC3 interaction is crucial to amplify Parkin-mediated mitophagy 109. Ambra1 has been identified as a Parkin interactor during mitochondrial depolarization. Although Ambra1 is not required for the translocation of Parkin to depolarized mitochondria, Ambra1 is involved in the clearance of mitochondria after Parkin translocation. Ambra1 knockdown inhibits CCCP-induced mitophagy, but in contrast overexpression of Ambra1 promotes mitophagy in the presence of Parkin. Mechanismly, recruitment of Ambra1 induced by Parkin activates class III PI3K around depolarized mitochondria 110. It has been revealed that Ambra1 regulates the activity and stability of ULK1 by promoting the ubiquitylation and self-association of ULK1 through tumor necrosis factor receptor-associated factor 6 (TRAF6). ULK1 has been shown to activate Ambra1 by phosphorylation. These regulatory events represent a positive regulation loop to sustain autophagy 111.

Advances in methodology have also boosted the progress of basic research. A quantitative proteomics approach to measure the dynamics, site-specificity and stoichiometry of Parkin-dependent substrate ubiquitination was reported, which provides a quantitative analysis of relevant pathways 112. Using this technology, Harper *et al*. demonstrated that hyper-phosphorylation of Ub chains on mitochondria inhibited the recruitment of the mitophagy receptor OPTN 113. OPTN and NDP52 (also known as CALCOCO2) function as the bridge connecting ubiquitin-proteasome system (UPS) and autophagy, since they can bind both ubiquitin and LC3/GABARAP 114. PINK1 recruits NDP52 and OPTN to damaged mitochondria independently of Parkin 53. Moreover, ubiquitinated mitochondria serves as a self-reinforcing positive feedback signal to coordinate TANK-binding kinase 1 (TBK1) activation. Hence, TBK1 activation-mediated phosphorylation of OPTN and NDP52 establishes a second feed-forward mechanism to promote mitophagy 108, 115 (**Figure 2**). Interestingly, a recent study revealed that PINK1/Parkin mediated mitophagy depending on LC3/GABARAPs-driving recruitment of OPTN and NDP52, in addition to phospho-ubiquitin. This pathway functions as a complementary mechanism to promote effective clearance of damaged mitochondria 116.

Several studies indicate an important role for P62 (also known as sequestosome 1, SQSTM1) in mitophagy. On one hand, P62 promotes mitochondrial ubiquitination independently of PINK1 and Parkin during mitophagy 117. On the other hand, P62 prevents excessive inflammasome activation depending on Parkin-mediated ubiquitination of damaged mitochondria 118. Parkin can also ubiquitinate NIX to induce mitophagy, by recruiting NBR1 to damaged mitochondria 54. NBR1, a functional homolog of P62, is dispensable for Parkin-mediated mitophagy regardless of the presence or absence of P62 55 (**Figure 2**).

Dynamic balance of mitophagy plays a vital role in physiological and pathological processes. Too much mitophagy is harmful to the cellular dynamic equilibrium. Consequently, this pathway must be counteracted by negative regulators. Ubiquitin-specific protease 15 (USP15), a cytoplasmic deubiquitinating enzyme (DUB), can resist Parkin-mediated mitophagy by attenuating ubiquitination 119. In addition, the mitochondrial DUBs USP30 and USP35 are two other factors that reduce mitophagy. USP30 and USP35 remove ubiquitin chains from mitochondrial proteins rather than affecting autoubiquitination and mitochondrial localization of Parkin 119, 120. Degradation of USP30, which is a mitochondrion-anchored protein as well as a Parkin substrate, leads to the effective elimination of damaged mitochondria 121-123. An earlier study suggested that pSer65-Ub was resistant to deubiquitinases because it has an altered structure. Consequently, dephosphorylation of ubiquitin was considered to be a negative regulatory mechanism in mitophagy 97. The latest research demonstrates that phosphatase and tensin homolog-long (PTEN-L, an OMM protein) is a novel negative regulator of mitophagy. Mechanistically, PTEN-L prevents translocation of Parkin to mitochondria, reduces Parkin phosphorylation (pSer65), and inhibits its E3 ligase activity, consequently maintaining Parkin in a closed inactive conformation 124.

Impairment of mitophagy is a well-established pathological mechanism implicated in Parkinson's disease (PD). Parkin is implicated in the pathogenesis of PD but the mechanism is still elusive. A recent study delivered insights from a different angle. The researchers investigated the role of Parkin in mouse skeletal muscle. They reported that the mitochondrial function of skeletal muscles was dramatically disabled in Parkin knockout mice, and this was accompanied by decreased expression of mitofusin 2 (MFN2), an OMM protein that mediated mitochondrial fusion 125. Interestingly, other studies have found that Parkin is required for ubiquitination of mitochondrial MFN1 and MFN2 126, 127, which ensures that MFN proteins are degraded during the translocation of Parkin to senescent and damaged mitochondria. This mechanism effectively guarantees the complete clearance of damaged mitochondria by mitophagy. It has been revealed that MFN2 mediates Parkin recruitment to damaged mitochondria in a PINK1-dependent manner. Phosphorylation of MFN2 by PINK promotes Parkin-mediated ubiquitylation, hence accelerating the culling of damaged mitochondria 128.

Mitochondrial impairment also induces an interaction between leucine-rich repeat kinase 2 (LRRK2), PINK1/Parkin and Mitochondrial Rho GTPase 1 (Miro1, alternatively referred to as RHOT1, an OMM protein), leading to the proteasome-mediated degradation of Miro1. The degradation of Miro1 is important because it halts mitochondrial movement and functions to quarantine damaged mitochondria for engulfment by phagophores 129, 130. However, mitochondrial translocation of Parkin was still observed in cells carrying LRRK2^ G2019S^, which has a mutation in the kinase domain. Interestingly, overexpression of Parkin in LRRK2^G2019S^ mutant cells does not rescue the delayed degradation of Miro1, which indicates that both the PINK1/Parkin and LRRK2 pathways are necessary for the degradation of Miro1 130. These studies revealed that Miro1 degradation-mediated mitophagy might serve as a mechanistic link between sporadic and familial Parkinson's disease 131. Additionally, mitochondrial stress seems to be an inducing factor to trigger innate immunity, and mitophagy induced by the PINK1/Parkin pathway can mitigate stimulator of interferon genes (STING)-induced inflammation, hence preventing neurodegeneration 132. These studies enrich our understanding of the role of mitophagy in the pathogenesis of PD. Intriguingly, basal mitophagy can occur independently of PINK1 in various tissues, and the degree of mitophagy depends on the metabolic context 133. Nevertheless, much work is still needed to identify the key regulators of basal mitophagy.

Additionally, mitochondrial fission is thought to be important for mitophagy. DRP1, a large GTPase, is the major protein effector of mitochondrial fission. DRP1 interacts with four mitochondrial receptor proteins: fission 1 (Fis1), mitochondria fission factor (Mff), mitochondrial dynamics protein of 49 and 51kDa (MID49/51) 134, 135. The translocation of DRP1 from cytosol to mitochondria promotes fission 136. Accumulating evidence indicates that posttranslational modifications of DRP1 is an important mechanism for regulating its function 137. Phosphorylation of DRP1 at S637 and dephosphorylation at S616 lead to the inhibition of mitochondrial fission 138-140. However, phosphorylation of DRP1 at S585 increases DRP1 activity 141.

Mitophagy is crucial for cellular homeostasis and function. Accumulating evidence shows that mitophagy is significantly involved in several human pathologies such as NDDs, cardiovascular pathologies (CVDs) and cancer 142. Therapeutic interventions aiming at counterbalancing mitophagy might be a promising approach to ameliorate these dysfunctions. Interestingly, DRP1-dependent mitophagy plays a protective role against pressure overload-induced mitochondrial dysfunction and heart failure 143 and cisplatin-induced acute kidney injury (AKI) 144. Moreover, DRP1-dependent mitochondrial remodeling and autophagy play an important role in neuronal differentiation 145. The crosstalk between mitophagy and bacterial pathogen infection has also been revealed. It has been reported that the intracellular bacterial pathogen *Listeria monocytogenes* can induce mitophagy in macrophages. Interestingly, levels of mtROS were significantly increased in *Nlrx*^-/-^ peritoneal macrophages (PMs). Given that mtROS has a pivotal role in controlling infection 146, 147, and accumulation of damaged mitochondria correlates with higher mtROS production 148, it seems that *L. monocytogenes* exploits host mitophagy through NLRX1 to remove damaged mitochondria, hence reducing mtROS and benefiting its survival. Mechanistically, *L. monocytogenes* and listeriolysin O (LLO, a virulence factor) promote mitophagy by inducing the oligomerization of NLR family member X1 (NLRX1), which promotes the binding of the LIR motif to LC3 149. Thus, targeting the mitophagy machinery might be useful for developing effective strategies against infections.

## ER-phagy: an important process for ER quality control

ER is an organelle that is composed of membranous sheets and tubules. ER creates, packages and secretes many of the proteins and lipids in eukaryotic cells. Hypoxia, aggregation of unfolded proteins, energy deprivation and other stimulating factors can lead to ER stress. ER stress is involved in many diseases including inflammatory diseases, cancer, diabetes, non-alcoholic fatty liver disease (NAFLD) and NDDs 150-152.

ER turnover and modulation are dynamically regulated and continuously adjusted to meet different cellular requirements. To alleviate stress and reestablish homeostasis, the ER activates intracellular signal transduction pathways, termed the ER unfolded protein response (UPR). Emerging evidence suggests that non-selective autophagy occurs during ER stress 153, 154, and this ER stress-induced autophagy has two main functions. The first is the formation of ER-containing autophagosomes (ERAs), which engulf the ER or aggregated proteins that cannot be processed by other pathways. The second function is to reduce the size of the expanded ER to normal levels when the ER-stress fades away. However, unlike non-selective autophagy during ER stress, ER-phagy occurs continuously under normal conditions and is enhanced during starvation. Mechanistically, less is known about the regulation of ER-phagy. The concept of ER-phagy originally appeared in 2007, to describe the induction of selective autophagy of the ER in yeast induced by ER stress 155.

### ER-phagy in yeast

In yeast, ER-phagy is divided into two types: macro-ER-phagy and micro-ER-phagy. Here we will primarily discuss macro-ER-phagy, a selective autophagy pathway. This pathway usually depends on Atgs and Ypt/Rab guanosine triphosphatases (GTPases). Three distinct steps are required for macro-ER-phagy. In the first step, Atg9 functions upstream of other phagophore assembly site/pre-autophagosomal-structure (PAS) organizers in macro-ER-phagy and is necessary for the formation of ER-to-autophagy membranes (ERAM). In addition, overexpressed Snq2 and Snc1 function as macro-ER-phagy cargo to form ERAM. In the second step, Ypt1 and core Atgs mediate PAS formation. Lastly, Atg8 and vacuolar protein sorting 21 (Vps21, yeast ortholog of human Rab5) are required to mediate the fusion of autophagosomes with the vacuole 156. The ER-resident membrane proteins Sec61 and high mobility group 1 (Hmg1) are transported together with Snc1 to the vacuole via macro-ER-phagy.

Atg9 is the only characterized transmembrane Atg protein. Atg2 and Atg18 can regulate Atg9 by mediating its retrograde return from the PAS to peripheral sites 157. However, Atg9 functions upstream of Ypt1. Ypt/Rab GTPases regulate all membrane transport events in eukaryotic cells. Ypt1 is a well-known critical factor in ER-to-Golgi transport 158. Nevertheless, not much is known about Ypt1 in the regulation of ER-phagy. Evidence indicates that membrane proteins accumulate in the abnormal ER in *ypt1* mutant cells. Trs85 functions upstream of Ypt1 to clean out accumulated ER proteins through autophagy 159, 160. Atg11 plays a crucial role in all types of selective autophagy. Furthermore, overexpression of Atg11 can rescue the GFP-Snc1-PEM intracellular accumulation and UPR induction phenotypes of *ypt1-1* mutant cells. All these lines of evidence indicate that Trs85-Ypt1-Atg11 mediate autophagic degradation of excess ER proteins 161. Using yeast cells expressing GFP-Atg8, it was found that GFP-Atg8 located close to ERAs, which indicated that Atg8 was involved in ER-phagy 162.

Drug-induced ER-phagy is a non-selective autophagy process, named micro-ER-phagy. It does not use autophagic organelles and machinery. Walter and his colleagues found that tunicamycin-induced Yop1-Pho8∆60 activation was reduced about 30% in mutants lacking Atg1, Atg6, Atg7, Atg8, Atg14 or Atg16, indicating that the core autophagy machinery was not essential for ER-phagy. Furthermore, they verified that autophagy of the ER did not require the EGO complex, the VTC complex or the nucleus-vacuole junction. Cells with* vacuolar protein sorting 34 (Vps34)* and *Vps23* mutation still showed ER whorls in the vacuole under ER stress. All these data indicated that the core autophagy machinery was not essential for non-selective autophagy of the ER 163.

Interestingly, researchers identified two novel receptors for selective ER-phagy in yeast, termed Atg39 and Atg40. These receptors mediated autophagic degradation of distinct ER subdomains. Atg39 localizes to the perinuclear ER, so it induces autophagic sequestration of part of the nucleus. In contrast, Atg40 is enriched in the cortical and cytoplasmic ER, and induces engulfment of these ER subdomains by autophagosomes (**Figure 3B**). In general, Atg39-dependent ER-phagy is required for cell survival under conditions of nitrogen deficiency. However, Atg40 is more likely to act as the yeast counterpart of family with sequence similarity 134, member B (FAM134B, a mammalian ER-phagy receptor) 164. However, it remains to clarify whether these two receptors can co-operate in a common ER-phagy pathway. Interestingly, researchers found that an ER membrane protein, Lnp1, was also involved in yeast ER-phagy. In *lnp1* mutant cells, the localization of Atg40 to sites of autophagosome formation was blocked, and this interfered with the interaction between Atg40 and Atg11 and the packaging of the ER into autophagosomes 165.

### ER-phagy in mammals

In mammals, there is also a receptor-mediated ER-phagy pathway. Currently, eight specific receptors are involved in mammalian ER-phagy: FAM134B, translocation protein SEC62 (SEC62), reticulon 3 (RTN3), cell cycle progression 1 (CCPG1), atlastin 3 (ATL3), testis-expressed protein 264 (TEX264), tripartite motif containing 13 (TRIM13, a transmembrane E3 ligase, also known as RFP2) and CALCOCO1 166-169 (**Figure 3A**).

FAM134B is responsible for the turnover of ER sheets and it can facilitate ER-phagy by binding to LC3 and GABARAP. An in-depth study indicates that down-regulation of FAM134B causes expansion of the ER, while overexpression of FAM134B leads to ER fragmentation and lysosomal degradation. Physiologically, mutant FAM134B results in sensory neuron dysfunction. This phenomenon is consistent in humans and mice 170. It has been reported that ER expansion is required for the biogenesis of the viral replication complex 171. However, FAM134B-dependent ER-phagy has also been reported to be an important limiting event in Ebola virus (EBOV) replication in mouse cells due to its negative regulation of EBOV replication 172. Moreover, knockdown of FAM134B modestly enhanced the replication of the Zika virus (ZIKV) and Dengue virus (DENV) in human cells 173.

SEC62 is a candidate oncogene that is frequently amplified in prostate, non-small cell lung, thyroid cancers, and head and squamous cell carcinoma 174-177. Interestingly, SEC62 has been identified as an ER-phagy receptor that regulates the size and function of ER once the stress is resolved. Mechanistically, the conserved LIR in the C-terminal cytosolic domain of SEC62 is required for ER-phagy. SEC62 is a translocon component. Since functional disturbance of the translocon complex does not affect SEC62-mediated ER-phagy, it is thought that SEC62 exits the translocon complex prior to the induction of ER-phagy 178. High levels of SEC62 are accompanied by enhanced ER-phagy activity. Enhanced ER-phagy activity increases the ability of cells to eliminate excessive and damaged ER, thereby enhancing the tolerance of ER stress and reducing cell death induced by ER stress 179.

RTN3, another specific receptor, is responsible for the degradation of ER tubules. Structural analysis of the RTN3 protein revealed several LIRs in the long N-terminal region of RTN3. The other three RTN family members, RTN1, RTN2, and RTN4, do not directly bind to LC3 180. Considering that reticulon 3 long isoform (RTN3L) and FAM134B share many potential interactors, we wonder if any regulatory mechanism exists between RTN3L and FAM134B. Interestingly, FAM134B interacts only with RTN2L and short RTN isoforms instead of RTN3L. RTN3L mediates ER-phagy in a FAM134B-independent manner, and this may be due to the different localization and distribution of these two receptor proteins in ER subdomains.

Smith *et al.* have identified CCPG1 as a new mammalian ER-phagy receptor. CCPG1 is an ER-resident transmembrane protein. The expression of CCPG1 is activated upon UPR. A mechanistic study uncovered that CCPG1 directly bound to LC3 and FAK-family kinase interacting protein of 200 kDa (FIP200) via a LIR motif and a FIR motif, respectively. Briefly, the C-terminal domain of CCPG1 is responsible for the recognition of misfolded or aggregated cargoes within the ER lumen, whereas the LIR and FIR motifs are necessary for the assemblage of CCPG1-cargo complexes and the recruitment of autophagic membrane 181, 182.

ATL3 belongs to a family of dynamin-like GTPases that function in ER fusion. ATL3 has been identified as a receptor for selective turnover of tubular ER by autophagy following the onset of starvation. Unlike other autophagy receptors, ATL3 specifically binds to GABARAP subfamily proteins, but not LC3, via two GABARAP interaction motifs (GIMs). Two ATL3 mutations, Y192C and P338R, which are associated with hereditary sensory and autonomic neuropathy type I (HSAN I), disrupt the binding of ATL3 to GABARAP and impair ATL3's function in ER-phagy. This indicates that defective ER-phagy is involved in HSAN I 167.

Interestingly, TEX264 has been identified as a ubiquitously expressed ER-phagy receptor 168, 183. TEX264 is an ER protein with a single-span transmembrane domain and a conserved LIR (**Figure 3A**). TEX264 also interacts with the ULK1 and phosphoinositide 3-kinase (PI3K) complexes 183. Among the ER-phagy receptors, TEX264 seems to interact with LC3 and GABARAP family proteins most efficiently. ER-phagy is greatly reduced by deletion of TEX264 alone and almost completely abolished by additional deletion of FAM134B and CCPG1. Also, the long intrinsically disordered region (IDR) near the LIR of TEX264 is required for autophagosome binding and ER-phagy 168. Identification of TEX264 as an ER-phagy receptor enriches our knowledge of the mechanisms that underlie ER-phagy.

Given that endogenous BNIP3 localizes to both mitochondria and ER, it is very likely that BNIP3 promotes ER-phagy via its interaction with LC3 47. Intriguingly, the mitophagy receptor BNIP3 and the general autophagy receptor P62 have been reported to participate in ER turnover 184, 185. In mammals, the liver is the major site for drug metabolism, and the ER in liver cells serves as the main organelle for synthesis of the vital metabolic enzyme cytochrome P-450 (CYP). More than 50% of drugs are oxidatively metabolized by CYP in the liver. On one hand, sufficient ER is needed for rapid synthesis of CYP during drug metabolism; on the other hand, the excess ER and CYP must be removed to maintain cellular homeostasis after the drug metabolism is complete. Consequently, the role of ER quality control in drug metabolism should not be underestimated 186. Ding and co-workers have demonstrated that autophagy plays an important role in removing excess hepatic ER. Inhibition of autophagy contributed to the expression of CYP2B even after withdrawal of the hepatic CYP2B inducer TCPOBOP (hepatic mitogen 1, 4-bis [2-(3,5-dichloro-pyridyloxy)] benzene). Additionally, P62 knockout mice had an increased ER content in the liver compared with wild-type mice after withdrawal of TCPOBOP. Based on the critical role of autophagy receptors in selective autophagy 21, 187, 188, it is likely that excess hepatic ER is removed via ER-phagy that is initiated by the interaction between LC3 and ubiquitin-P62-decorated ER 185. Nevertheless, further research is needed to elucidate the underlying mechanism of Ub-dependent ER-phagy. TRIM13 has been identified to be an ER-associated receptor of P62 in ER-phagy. The interaction between TRIM13, Beclin-1 and VPS34 is indispensable for ER membrane curvature and autophagosome biogenesis. Moreover, K63-linked Ub on TRIM13 recruits P62, hence promoting ER-phagy 169.

CALCOCO1 is a novel soluble ER-phagy receptor for the degradation of tubular ER in response to proteotoxic- and nutrient stress. CALCOCO1 binds directly to Atg8 proteins via LIR- and UDS-interacting region (UIR) motifs. Unlike the other ER-phagy receptors, CALCOCO1 is peripherally associated with the ER, targeting to it by interacting with VAMP-associated A (VAPA) and/or VAPB (ER transmembrane proteins) via a FFAT-like motif. VAPs are involved in forming contacts between the ER and other membranes. Loss of VAPs impairs CALCOCO1-mediated degradation of tubular ER 189 (**Figure 3A**). Whether CALCOCO1 cooperates with other proteins needs to be addressed in the future. An important question is how to regulate CALCOCO1-mediated ER-phagy, considering the competition of FFAT-containing proteins for VAP interaction. Other roles of CALCOCO1-VAP coupling besides ER-phagy are also worth exploring, given that CALCOCO1 is localized in the Golgi and constitutes part of ER-Golgi contact sites.

Achievements have been made in ER-phagy that is mediated by the interaction between ER transmembrane proteins and phagophores. In conclusion, FAM134B, SEC62, RTN3, CCPG1, TEX264 and CALCOCO1 all have the LIR domains. However, ATL3 specifically binds to GABARAP subfamily proteins, but not LC3, via two GIMs. Interestingly, TEX264 seems to interact with LC3 and GABARAP family proteins most efficiently. TRIM13 functions in Ub-dependent ER-phagy. It has been identified to be an ER-associated receptor of P62 in ER-phagy. However, open questions still remain regarding whether and how ER-phagy receptors recognize specific ER subdomains. What is the specific role of Lnp1 in ER-phagy? Also, considering that the interaction between CCPG1 and LC3 is sufficient to cause ER-phagy, what is the role of FIP200 in this process? Given that the ER is a highly dynamic intracellular organelle with a large membrane, and that the ER may be a major source for autophagic membranes, it would be of great significance to investigate the role of ER-phagy receptors in the formation and elongation of autophagic membranes. In addition, further studies are required for the investigation of posttranslational modification of ER-phagy receptors during ER-phagy.

ER-stresses induced by unfolded or misfolded proteins aggregating within the ER lumen have been considered to be the important pathological mechanisms of NDDs, metabolic disorders and cancers 190. In a recent review, Dikic *et al*. summarize ER-phagy receptors that are involved in various human disorders, such as cancer, Alzheimer's disease (AD) and vascular disease 191. Taken together, targeting ER-stresses and characterizing the role of different ER-phagy receptors will be helpful for our understanding of their physiological and pathological roles and evoke a promising therapeutic field in the future.

## Proteaphagy: selective autophagy of inactive proteasomes

Three classical pathways of protein degradation in eukaryotic cells have been elucidated: the autophagy-lysosome pathway, the caspase pathway, and the ubiquitin-proteasome pathway 192, 193. In the UPS, multiple ubiquitin molecules are covalently attached to target proteins and degraded by the 26S proteasome. Proteasomes themselves can also undergo degradation. The lysosomal degradation of proteasomes was first identified in 1995, when it was found that proteasomes were accumulated in the lysosomes of rats treated with leupeptin (an inhibitor of lysosomal proteases) 194.

The concept of proteaphagy, was originally proposed in 2015 in *Arabidopsis*. It was reported that 26S proteasome subunits were increased in autophagy mutants, and the excess 26S proteasomes were degraded by Atg8-mediated autophagy 195. There are two distinct types of proteaphagy: one is nonselective proteaphagy, which is induced by starvation; and the other is selective proteaphagy, which is induced by proteasome inhibitors and requires the ubiquitination of inactive proteasomes 196, 197 (**Figure 4**).

### Proteaphagy in yeast

Proteasome inhibitors like MG132 induced proteaphagy, and inhibitor-induced proteaphagy was blocked in a regulatory particle non-ATPase 10 (RPN10) mutant. A mechanistic study identified *Arabidopsis* RPN10 as a new selective autophagy receptor that targeted inactive 26S proteasomes by interacting with Atg8 via a specific ubiquitin-interacting motif 2 (UIM2) and ubiquitinated proteasome subunits 195, 197. Interestingly, the inactive 26S proteasomes underwent degradation in a similar fashion in yeast, just using different ubiquitin receptors. Evidence has revealed that MG132 triggers ubiquitylation of proteasomes and association with Cue5. Further research revealed that Cue5 synchronously binds ubiquitin and Atg8 in yeast proteaphagy, and an oligomeric heat shock protein 42 (Hsp42) chaperone is also required in this process 198, 199 (**Figure 4A**).

### Proteaphagy in mammals

In mammals, the proteasome subunits RPN1, RPN10 and RPN13 have been identified as major ubiquitin receptors 200, 201. These three subunits are poly-ubiquitinated upon amino acid starvation, which facilitates their recognition by the autophagy receptor P62. By simultaneous interaction with LC3, P62 delivers inactive 26S proteasomes to the expanding phagophore for eventual turnover by autophagy, a process requiring the target of rapamycin (TOR) kinase 202-204 (**Figure 4A**). Transport of proteasomes can also be mediated by a heat shock cognate protein 73 (HSC73) under starvation conditions 194. However, further work will be required to uncover the details of how HSC73 and ATP are involved in the selective degradation of proteasomes.

Ubiquitin carboxyl-terminal hydrolase 3 (Ubp3) has been reported to play a role in proteaphagy in yeast under nitrogen starvation. This process involves dissociation of the proteasomal core particle (CP) and regulatory particle (RP), nuclear export, and independent vacuolar targeting of CP and RP 205. The CP and RP coalesce into cytoplasmic foci in a sorting nexin (Snx) 4/41/42-dependent manner during nitrogen starvation or proteasome inhibition. As this is dependent on Snx4's capacity to bind to phosphatidylinositol 3-phosphate (PtdIns3P or PI3P)-containing membranes, it may function by recruiting cargo to the autophagic membrane. Disruption of Snx4 localization also compromises proteaphagy 206. Interestingly, Snx4 is required for both proteaphagy and ribophagy, and Ubp3 is the common regulator of both proteaphagy and ribophagy 206-208. In contrast, during carbon starvation, proteasomes are reversibly sorted to avoid autophagic degradation. This process requires Blm10 for the CP, Spg5 and the C-terminus of Rpn11 for the RP and the Nat3/Mdm20 complex for both 209 (**Figure 4B**).

Since most proteaphagy research has been carried out in yeast, there are still many unsolved mysteries that need further exploration. Is there conservation between mammalian proteaphagy and yeast proteaphagy? Is there a different selective autophagy receptor for proteaphagy? Our understanding of proteaphagy is very limited at present, and future research will undoubtedly fill in additional mechanistic details of this intriguing and critical process of protein quality control.

Protein aggregation can also be degraded by aggrephagy, a selective disposal of protein aggregates by autophagy 210. Overexpression of toll interacting protein (Tollip) leads to efficient degradation of Huntington's disease (HD)-linked polyglutamine (polyQ) proteins. Conversely, Tollip depletion causes cytotoxicity toward overexpression of polyQ proteins. Mechanistically, Tollip binds to and colocalizes with ubiquitin and LC3, indicating that Tollip is an ubiquitin-Atg8 receptor 211, 212. The autophagy receptors P62 and NBR1 and the large adapter protein autophagy-linked FYVE (ALFY) also play important role in aggrephagy 213, 214. Interestingly, OPTN was recently found to be involved in aggrephagy 215, 216. Aggrephagy is regulated at three levels: (1) the level of autophagic machinery; (2) the level of autophagy receptors (P62, NBR1, ALFY and OPTN); and (3) the level of the protein aggregates 217. Although these autophagy receptors are subjected to a variety of posttranslational modifications including phosphorylation, ubiquitination, and acetylation as in other selective autophagy processes 218, hitherto, the mechanism of aggrephagy is still poorly understood. Major NDDs are characterized by aberrant protein aggregations in neurons, glial cells and/or extracellular plaques. Given that neurons are largely nondividing cells, even a subtle detrimental effect on the cell's capacity over enough time can eventually damage neurons. As expected, aggrephagy is a critical mechanism involved in the pathogenesis of NDDs 219. However, whether modulation of aggrephagy is a therapeutic strategy for NDDs and other proteinopathies is an open question. Amyotrophic lateral sclerosis (ALS) is characterized by accumulated protein aggregates, dysfunctional mitochondria, and stress granules (SGs). In ALS, these cargoes are targeted to selective autophagy for degradation. Dysfunctions of autophagy receptors/adapters in selective autophagy thus may be related to the pathogenesis of ALS. Hence, manipulating autophagy activity has been considered as a therapeutic approach for treating this disease 220.

## Ribophagy: selective autophagic degradation of protein production factories

Like the proteasome, ribosomes have also been detected inside autophagosomes by electron microscopy 221. For a long time, it was assumed that these autophagosome-engulfed ribosomes came from the nonselective bulk degradation pathway. However, emerging evidence has indicated connections between ribosomes and selective autophagy. Ribophagy, as the name implies, is a special type of autophagy that selectively degrades ribosomes (**Figure 5**).

Ribosomes are essential cellular organelles that constitute about 50% of all cellular proteins. The number and quality of ribosomes are therefore of great significance. Inclusion of ribosomes into autophagosomes, for instance, may be involved in the neuroprotective effect of neonatal hypoxia-ischemia 222. Amino acid or insulin deprivation can induce selective degradation of cytoplasmic RNAs by autophagy in rat hepatocytes 223. Kraft *et al*. showed that inhibiting starvation-induced ribophagy can accelerate cell death 207. In zebrafish, genomic mutations leading to ribosomal stress cause severe defects in intestinal, liver, pancreas and craniofacial development. Interestingly, autophagy may function as a response mechanism to promote survival in this case 224.

### Ribophagy in yeast

It has been reported that the degradation of 40S and 60S subunits in yeast is abolished in *atg7 deficient* cells, based on the Atg7's essential role in the Atg12 and Atg8 conjugation systems. It has also been documented that mature ribosomes are rapidly degraded by autophagy under nutrient starvation in *S. cerevisiae*. This process requires the Ubp3p/Bre5p ubiquitin protease. However, 60S ribosomal proteins accumulate in cells without Ubp3 and Bre5 225. Interestingly, the Ubp3-Bre5 complex seems to regulate mitophagy as well as ribophagy 226. Ribophagy is an evolutionarily conserved pathway. The mammalian homologs of Ubp3 and Bre5 are USP10 and G3BP 227. USP10 has no currently known function. Ras-GTPase -activating protein (GAP)- binding protein (G3BP) has been assigned a role in constraining the formation of SGs by interacting with 40S ribosomal subunits through its RGG motif (a RNA-binding domain), and it also functions in the clearance of SGs by autophagy 228, 229. Peter and Kraft found that the degradation of 60S large ribosomal subunits in yeast required the participation of Ubp3 and Bre5. In addition, Rsp5, an ubiquitin ligase, appeared to work in ribophagy together with Ubp3 230. Although Rsp5 is not essential for ribophagy, its substrates are ribosomal proteins 231. Thus, further work will be required to identify the relevant ubiquitin ligase that may be involved in the regulation of ribophagy under nutrient starvation. Dargemont's team has identified two factors, Cdc48 and Ufd3; that participate in starvation-mediated ribophagy depending on the Ubp3-Bre5 complex. These two factors collaborate to recognize and deubiquitinate a yet-to-be identified ubiquitinated target 232. Therefore, some effort is still needed to identify the potential substrate of Ubp3-Bre5. The 60S ribosomal protein Rpl25 has been identified as a substrate of Ubp3 and listerin (Ltn1). Ubiquitylation of Rpl25 prevents 60S ribophagy. Upon starvation, Ubp3-mediated de-ubiquitination of Rpl25 accelerates the selective autophagy of 60S ribosomal subunits 225 (**Figure 5A**). Moreover, the Ubp3-Bre5 complex can interact with Atg19, the receptor of Ape1 and Ams1, and regulate the ubiquitination of Atg19 233.

Ribosomes can be degraded via autophagy induced by starvation, mammalian target of rapamycin complex 1 (mTORC1) inactivation, or translational inhibition by arsenite 234, 235. Interestingly, some kinases that regulate the degradation of ribosomes by macroautophagy are also involved in ribophagy. Rim15 (a direct downstream protein kinase of Sch9) plays the role of a double-edged sword in the degradation of ribosomes. On one hand it upregulates ribophagy, while on the other hand, it downregulates the non-selective degradation of ribosomes after target of rapamycin complex 1 (TORC1) inactivation 236. In non-selective ribosome degradation, the expression of TORC1 is down-regulated after nutrient starvation. TORC1 phosphorylates six residues in the C-terminus of the AGC kinase Sch9, so the phosphorylation of these residues is lost under starvation conditions 237. Sch9 negatively regulates Rim15 via phosphorylation 236. Rim15 is also regulated by protein kinase A (PKA) and Pho85 238, 239. However, it is still unclear whether Sch9 controls every event via Rim15. Interestingly, this non-selective degradation process takes place in an Atg11-independent way 240 (**Figure 5A**). Surprisingly, in plants, the endoribonuclease Rns2 is required for ribosomal RNA degradation in a ribophagy-like pathway. However, further studies are required to determine whether Rns2 is involved in the degradation of ribosomes 241. As previously reported, ribophagic flux in yeast is largely Atg8-dependent 207, 242. In contrast, the Atg8 conjugation system seems not to be essential for starvation-induced ribophagic flux in mammals, even though its deletion reduces the closure of autophagosomes 243. Harper *et al*. applied Ribo-Keima flux reporters to show that starvation or mTOR inhibition promoted VPS34-dependent ribophagic flux 235.

### Ribophagy in mammals

Although selective factors for ribophagy in yeast were discovered many years ago, the receptors specific for mammalian ribophagy have not been characterized. It was shown that nuclear fragile X mental retardation-interacting protein 1 (NUFIP1), a protein previously known to be localized in the nucleus, and its binding partner zinc finger HIT domain-containing protein 3 (ZNHIT3) redistributes from the nucleus to autophagosomes, lysosomes and ribosomes upon mTORC1 inhibition. ZNHIT3 delivers ribosomes to autolysosomes by directly binding to LC3B. These results suggest that NUFIP1 is a mammalian ribophagy receptor 234, 244 (**Figure 5B**). However, it is still unclear which part of the 60S ribosomal subunit functions as the actual ligand.

It is worth mentioning that Wyant *et al*. have proposed a strategy for identifying novel selective autophagy receptors based on the common features of selective receptors. Given that all these autophagy receptors will eventually be degraded in lysosomes, it is possible to identify additional receptors for selective autophagy in mammals by combining rapid method for the immunopurification of lysosomes (LysoIP) proteomic analysis with sequence analysis to search for potential LIRs 245, 246. This provides a new approach to finding more receptors.

## Pexophagy: a mechanism for peroxisome turnover and homeostasis

Peroxisomes are single membrane-enclosed organelles that are essential for the homeostasis of nearly all eukaryotic cells. A conspicuous characteristic of peroxisomes is their ability to multiply or be degraded in response to specific stimuli 247. In mammals, functional peroxisomes regulate a variety of cellular metabolic events, such as lipid generation and metabolism, inactivation of toxic substances, and regulation of oxygen concentration. Increasing evidences indicate that imbalanced peroxisome homeostasis may lead to a wide range of physiological disorders and associated diseases, such as Zellweger spectrum disorder, neonatal adrenoleukodystrophy, and infantile Refsum disease 248. Therefore, the quality control of peroxisomes is very important.

Peroxisomes have a half-life of 1.3~2.2 days 249, so efficient removal and generation of peroxisomes is necessary. There are three main ways to eliminate peroxisomes: (1) peroxisomal matrix proteins can be degraded by Lon protease (LONP) 250; (2) peroxisomes can undergo autolysis mediated by 15-lipoxygenase (15-LOX) 251, 252; and (3) peroxisomes can be selectively degraded by autophagy, a process known as pexophagy 253, 254.

Just like mitophagy, the pexophagy pathway also needs specific receptors and/or adapters. Upon induction of pexophagy, an adapter protein translocates onto the peroxisomal membrane and connects the peroxisome to an autophagosome. Five receptors/adapters have been identified among eukaryotes: Atg30 in *Pichia pastoris* (*P. pastoris*) 255, Atg36 in *Saccharomyces cerevisiae* (*S. cerevisiae*) 256, NBR1 257, P62 258, 259 and acyl-CoA-binding domain containing protein 5 (ACBD5) in mammals 260 (**Figure 6**).

Most pexophagy researchers have used methylotrophic yeasts as the model organism. In *P. pastoris*, Atg30 localizes at the peroxisome membrane and transiently translocates to the PAS during pexophagy. Atg30 controls pexophagy through the assembly of pexophagic receptor-protein complex (RPC). Overexpression of Atg30 can induce pexophagy, and Pex3 is necessary for the phosphorylation of Atg30 255. Pex3 mutant *P. pastoris* cells showed pexophagy defects because the mutant Pex3 protein was unable to bind Atg30 261. Subramani *et al*. have reported that Atg37 and ACBD5 (the human orthologue of Atg37) positively regulate the formation of RPC 262. Atg30 selectively degrades peroxisomes in a Pex3 and Atg37 dependent manner, by recruiting Atg8 and Atg11 to the RPC. Moreover, Atg30 is phosphorylated by Hrr25 kinase, and this phosphorylation can be regulated by Pex3 and Atg37, negatively and positively, respectively 263. Since Atg37 also functions as an acyl-CoA (Ac) binding protein, Ac might regulate the Atg30-Atg37 interaction, hence affects the recruitment of Atg11 to the pexophagic RPC 260 (**Figure 6A**).

PEX2, an ubiquitin ligase, also plays an important role in mammalian pexophagy. Expression of PEX2 leads to ubiquitination of peroxisomes and pexophagy in an NBR1-dependent manner. Under normal conditions, a low PEX2 expression level is maintained by mTORC1 via the proteasome pathway, which ensures that PEX5 is ubiquitinated and consequently recycled from the peroxisomal membrane. However, PEX2 expression is up-regulated by both amino acid starvation and rapamycin, and the increased PEX2 leads to the ubiquitination of PEX5 and the 70-kDa peroxisomal membrane protein (PMP70, a substrate of PEX2). Consequently, NBR1 is recruited, targeting ubiquitinated peroxisomes to autophagosomes for degradation 264. Interestingly, in addition to regulating mitophagy, USP30, the OMM protein, also targets to peroxisomes in a PINK1/Parkin-independent manner 265. Overexpression of USP30 prevents pexophagy during amino acid starvation, by counteracting the action of the peroxisomal E3 ubiquitin ligase PEX2 266, 267 (**Figure 6B**). Therefore, an important focus for future studies of USP30 may provide new targets and tools for studying pexophagy in human health and disease.

Several yeast peroxins (such as Pex5, Pex7 and Pex20) are ubiquitinated during the peroxisomal protein import cycle 268, but only PEX5 was identified as an ubiquitinated protein in mammals. Two independent reports indicate that ubiquitination of mammalian PEX5 also triggers pexophagy 269. The first report showed that mono-ubiquitination of PEX5-EGFP at C11 is crucial for triggering ubiquitin-dependent pexophagy 270. The second report demonstrated that ATM phosphorylates human PEX5 at S141 in response to reactive oxygen species (ROS), followed by mono-ubiquitylation of PEX5 at K209 271. Thereby, pexophagy was activated via binding of P62 to mono-ubiquitinated PEX5. Blocking the recycling of PEX5 from peroxisomes to the cytosol by PEX1 and PEX6 (anchored on peroxisomes via PEX26) will induce pexophagy 272. Pex14 plays a pivotal role during pexophagy in LC3-dependent manner in mammals, because the selective degradation of Pex14 and Pex13 was not observed in LC3-knockdown cells under nutrient deprivation. Interestingly, the binding of PEX5 and LC3-II to PEX14 is competitive. In normal conditions, PEX5 binds to PEX14, thus limiting pexophagy. Under starvation, PEX14 regulates pexophagy via interaction with LC3-II. Co-IP assays have identified that the binding region is proved to be at the N-terminus 273. On the other hand, ataxia-telangiectasia mutated kinase (ATM) can also repress mTORC1, a negative autophagy regulator. Therefore, AMPK, activated by ATM signaling, subsequently phosphorylates ULK1 to induce autophagy in mammals 61, 274-276. By interacting with PEX19 and PEX5, tuberous sclerosis complex (TSC) is localized to peroxisome membranes in response to ROS 277. Moreover, phosphorylated TSC, activated by ATM signaling, is also able to repress mTORC1 activity 278. ACBD5 is the only mammalian pexophagy-specific protein known to date. Studies have shown that ACBD5 might be involved in the recruitment of a pexophagy-specific receptor or adapter 260 (**Figure 6B**).

In mammals, NBR1 and P62 are multifunctional proteins involved in various processes, such as degradation of protein aggregates and midbody rings. The endogenous level of peroxisomes was not affected after knocking down the expression of the autophagy receptor NDP52 using siRNAs. However, siRNA knockdown of P62 and NBR1 in cells causes an increase in catalase levels, suggesting that P62 and NBR1 are both required for the basal turnover of peroxisomes. Since both NBR1 and P62 are involved in the process of peroxisome degradation, Kim *et al.* wondered whether these two proteins acted in two distinct parallel pathways. Interestingly, they found that NBR1 and P62 acted in the same pathway. They provided several lines of evidence that the amphipathic α-helical J domain and ubiquitin-associated (UBA) domain of NBR1 were required for pexophagy. Moreover, P62 was not required when cells overexpressed NBR1, but it increased the efficiency of NBR1-mediated pexophagy 257. It should be mentioned that both P62 and NBR1 are also involved in the selective degradation of other cargoes, in addition to pexophagy 21.

It is worth mentioning that Atgs also play an important role in pexophagy. Atg7 was the first gene that was identified to be required in mammalian pexophagy. In mammalian autophagy, LC3-I is lipidated to LC3-II by Atg7 and Atg3. The autophagic process is completely absent in Atg7 knockout mice after the induction of peroxisome proliferation by a 2-week treatment with 2% di-(2-ethylhexyl)phthalate (DEHP) 279. In *Arabidopsis*, the autophagosome marker Atg8 was observed to colocalize with peroxisome aggregates, suggesting that damaged peroxisomes would be selectively removed by autophagy. Moreover*,* pexophagy induced by hydrogen peroxide is deficient in *peroxisome unusual positioning 1* (*peup1*, a homolog of yeast Atg2) mutant cells 280. Atg5 and Atg7 are also required for pexophagy in *Arabidopsis* hypocotyls 281. Peroxisomes are highly dynamic organelles, changing in abundance in response to stress. Since DRP1-mediated mitochondrial fission facilitates mitophagy, future studies should reveal if peroxisomal fission also plays a role in pexophagy.

Peroxisomes are involved in redox balance, lipid metabolism and bile acid synthesis 282, 283. Therefore, regulation pexophagy levels might influence these biological processes. Tsvetkov *et al*. recently summarize peroxisomal dysfunction in age-related diseases in the central nervous system (CNS) 284. Understanding how pexophagy is regulated and the role of pexophagy in numerous physiological processes will help to develop novel strategies preventing peroxisomal dysfunctions-related diseases.

## Lipophagy: connecting autophagy and lipid metabolism

Intracellular triglycerides (TGs) and cholesterol are stored in LDs and metabolized by cytoplasmic neutral hydrolases. LDs are therefore the main source of cellular energy (via metabolism) and membrane components. The metabolism of cellular LDs appears to proceed via two main pathways: lipolysis and lipophagy (**Figure 7**).

Lipolysis refers to the catabolism process of TGs stored in cellular LDs 285. Perilipins (PLINs), which include five family members with similar sequences 286, play a role in regulating lipolysis by recruiting or preventing lipase activity on LDs. The LD coat proteins PLIN2 and PLIN3 are degraded through the coordinated action of Hsc70 and the lysosome-associated membrane protein type 2A (LAMP-2A) receptor by chaperone-mediated autophagy (**Figure 7**). Different PLIN members appear to recognize different sizes or maturation states of LDs for binding 287. Until recently, the mechanism by which each PLIN family member regulates lipolysis has not been well defined.

For a long time, it was thought that lipids could not be selectively degraded as autophagy substrates. The degradation of lipids via the autophagy-lysosome pathway has gained considerable attention. Lipophagy, the alternative pathway of lipid metabolism by the autophagy-lysosome system, is the other main lipid metabolic process 150, 288, 289. Lipophagy was initially described in hepatocytes, due to their ability to accumulate excess lipids. Using Atg7 knockout mice, Singh and colleagues have found that the LD lipolysis in hepatocytes depends on autophagy 288. Interestingly, knockout of Atg7 in brown adipose tissues (BAT) or liver blocks LDs breakdown in response to rapamycin 290. Subsequently, lipophagy has been found in different kinds of cells, such as hypothalamic cells 291, striatal neurons 292, glial cells 292, adipocytes, fibroblasts 293 and enterocytes 294.

ATG14, which contains a PE-binding region, interacts with ULK1 and LC3 to induce lipophagy, resulting in release of free fatty acids (FFAs). The released FFAs continually undergo mitochondrial β-oxidation. AMPK expression is activated by ER stress induced by metformin or nutrient restriction. AMPK then inhibits mTORC1, which triggers autophagy by inducing the formation of the autophagy initiation complex (ULK1, ATG13, FIP200, ATG101). Nutrient restriction also activates lysosome-mediated lipid metabolism. Rab7, a small GTPase, is dramatically activated in cells under nutrient depletion conditions. Depletion of Rab7 causes attenuated lipophagy in hepatocytes. A study of the underlying molecular mechanism reveals that Rab7 interacts with its downstream effector Rab-interacting lysosomal protein (RILP) to accelerate LDs breakdown. Consequently, Rab7 functions as a central regulator of hepatocellular lipophagy 295. In addition, the large GTPase dynamin2 (DNM2) functions in lipophagy. Genetic manipulation or pharmacological inhibition of DNM2 inhibits efficient catabolization of LDs under nutrient stress. However, overexpression of DNM2 can rescue the metabolic defects mentioned above 296 (**Figure 7**).

Interestingly, both lipolysis and lipophagy may be regulated by adipocyte triglyceride lipase (ATGL, also known as patatin-like phospholipase domain-containing protein 2 (PNPLA2)) 297. ATGL initiates triacylglycerol (TAG) hydrolysis to release FFAs 298. Singh *et al*. have revealed that ATGL contains LIR motifs within its patatin domain, and mutation in LIR displaces ATGL from LDs and blocks lipolysis. Hence, ATGL is suggested to act as a selective autophagy receptor for lipophagy 299 (**Figure 7**). Similarly, a recent study reveals that ATGL-induced lipophagy accelerates LD catabolism and the oxidation of hydrolyzed FFAs. Mechanistically, ATGL promotes Sirtuin 1 (SIRT1) activity, and SIRT1 is required for ATGL-mediated induction of lipophagy 300. Another lipase of the same family, PNPLA8, can also interact with LC3 to induce lipophagy in mouse model of a high fat diet 301 (**Figure 7**). In addition, PNPLA3 is required for lipophagy in starved human hepatocytes 302, and PNPLA5 contributes to lipophagy as well as proteolysis and mitophagy 303. It should be mentioned that all these lipases might also regulate autophagosome formation via mobilization of stored lipid content 304, 305.

Although the upstream signals or stimuli for activating lipophagy are not the same, the primary mechanisms of lipophagy activation are conserved in most cell types. For example, rapamycin-induced activation of lipophagy can increase the colocalization of BODIPY (LD marker) with LAMP1 (lysosomal marker) 288. Treatment with AMPK inhibitors reduces the kaempferol-induced co-localization of LDs with autophagosomes and lysosomes 306. Oxidative stress is alleviated by superoxide dismutase 1 (SOD1) converting superoxide (generated from ROS) to hydrogen peroxide. Interestingly, SOD1 deficiency inhibits lipophagy as evidenced by abnormally accumulation of LC3 and P62, leading to enhanced LD accumulation in the fasting mouse liver 307. Transcription factor E3 (TFE3) has been reported to function in autophagy flux, lysosome and hepatic lipid metabolism. Overexpression of TFE3 enhances autophagy flux and alleviates hepatic steatosis by increasing peroxisome proliferator-activated receptor-γ coactivator-1α (PGC1α)-dependent mitochondrial fatty acid β-oxidation. Mechanistic studies have revealed that TFE3 regulates PGC1α by binding to the promoter region of its cognate gene 308. Increasing evidence suggests that transcription factor-EB (TFEB) and P62 also regulate lipophagy under some physiological conditions, including exercise and fasting 309. The subcellular localization and activity of TFEB are regulated by mTOR-mediated phosphorylation. TFEB translocates from the cytoplasm to the nucleus after dephosphorylation, inducing transcription of numerous autophagy genes 310, 311. Interestingly, the role of TFEB in lipid catabolism is evolutionary conserved because the loss-of-function of *HLH-30* (worm ortholog of TFEB) also results in impaired lipophagy 309, 312. It has been revealed that trehalose treatment alleviates the mitochondrial dysfunction and attenuates cisplatin-induced kidney injury by activation of TFEB-mediated autophagy 313. Therefore, alternative strategies, such as inducing TFEB activity for only limited periods or enhancing only specific subsets of the TFEB-regulated gene network, should be considered when using TFEB as a therapeutic tool. Using a fusion protein of P62 and a LD-binding domain, Tsukamoto and co-workers have revealed that lipophagy is induced by localization of P62 on the surface of LDs, where it assists the association of LDs and the autophagosomal membrane 314. Intriguingly, lipophagy is also activated by high levels of dietary lipids. Pharmacological activation of peroxisome proliferator-activated receptor-α (PPARα) induces lipophagy. In contrast, pharmacological activation of farnesoid X receptor (FXR, a sensing nuclear receptor) suppresses the induction of autophagy in liver 315. Taken together, the above studies reveal that lipophagy can be regulated in multiple ways.

Lipophagy degrades TGs and cholesterol esters to supply FFAs that continually undergo mitochondrial β-oxidation in order to sustain cellular lipid content as well as energy homeostasis 316. Cellular TG content, as well as LDs number and size, are increased in response to inhibition of lipophagy by drugs or genetic manipulation 288, 316. Considering the concept that lipophagy is involved in a series of physiological processes, regulation of lipophagy may also be an effective strategy for the prevention and treatment of metabolic syndrome. Quercetin, a traditional Chinese medicine, has been widely used in the treatment of NAFLD. However, the mechanism underlying its activity remains unclear. Yao *et al*. have shown that quercetin ameliorates high-fat diet (HFD)-induced NAFLD by promoting hepatic very low density lipoproteins (VLDL) assembly and lipophagy via the inositol-requiring transmembrane kinase/endoribonuclease 1α (IRE1α)-X box binding protein 1 (XBP1) pathway 317. Interestingly, lipophagy also functions as a protective mechanism against alcoholic fatty liver disease (AFLD) caused by short-term ethanol supplementation 318. In addition to supplying free fatty acids to maintain cellular energy supply, lipophagy has some other functions. For example, it can prevent rat enterocytes from lipid toxicity owing to excess LDs accumulation 319. Moreover, lipophagy regulates cell death and inflammation in nonalcoholic fatty liver disease 320. Interestingly, flaviviruses can exploit lipophagy to drive virus production under the action of unmodified ancient ubiquitous protein 1 (AUP1, a type-III membrane protein) 321. Furthermore, accumulation of LDs is associated with neurotoxicity, and activation of lipophagy has been shown to reduce dihydroceramide-related neurodegeneration in drosophila 322. Lipophagy has been shown to play a dual role in cancer growth. On one hand, lipophagy supplies energy to tumor cells 323. On the other hand, lipophagy inhibits tumorigenesis, since lysosomal acid lipase (LAL, a lipase that facilitates lipophagy) exhibits tumor suppressor activity 324, 325. Hence, clarifying the mechanism of lipophagy helps to reveal therapeutic targets for these diseases.

In sum, the current research on lipophagy is mainly focused on its activation or inhibition to observe the role of lipophagy disorders in life activities. To date, only ATGL and PNPLA8 have been identified as lipophagy receptors in mammals. Much work is definitely required to clarify how ATGL regulates lipophagy and whether ATGL-mediated lipophagy is implicated in NDDs. Huntingtin (HTT) has been reported to function as a scaffold for selective autophagy 326. Lipids accumulated in cells expressing mutant huntingtin 327. Consequently, huntingtin might be an LD recognition receptor protein. Nevertheless, further research focusing on validating this hypothesis is necessary. Hence, identifying and characterizing the receptors of lipophagy will attract the interest of researchers'.

## Lysophagy: clearance of damaged lysosomes by autophagy

Lysosomes are the major acidic organelles for digesting unwanted intracellular materials. Lysosomes contain membrane proteins such as LAMP1 and more than 60 resident soluble hydrolytic enzymes 328. The release of large amounts of hydrolases from destabilized lysosomes into the cytosol is detrimental to the cell 329. Ruptured lysosomes also induce the release of protons and calcium from the lysosomal compartment to impair cellular functions 330. Cells are unable to maintain cellular homeostasis if damaged lysosomes are not removed, since the total number of intracellular lysosomes varies only slightly even if some of them become dysfunctional 331. Damaged lysosomes can be sequestered by autophagosomes in a process termed lysophagy 331, 332. Therefore, lysophagy plays an essential role in preserving the number of functional lysosomes and maintaining cellular homeostasis.

A variety of materials, including mineral crystals, bacterial or viral toxins, lipids, β-amyloid, and lysosomotropic agents such as L-leucyl-L-leucine methy ester (LLOMe), can induce lysophagy 329, 331, 333. These substances often cause Parkinson's disease and hyperuricemia nephropathy *in vivo* 331, 334*.* When lysosomal membranes are damaged or even under normal conditions, lysophagy factors such as ubiquitin conjugating enzyme E2Q family-like 1 (UBE2QL1), SKP1/CUL1/F-box protein (SCF)^FBXO27^, Leucine-rich repeat and sterile alpha motif-containing protein 1 (LRSAM1) and TRIM16 are recruited to ubiquitinate lysosomal membrane proteins. Ubiquitinated proteins then recruit autophagy adapters (such as TAXBP-1, SQSTM1, valosin-containing protein (VCP) and phospholipase A 2-activating protein (PLAA)), leading to induction of lysophagy. UBE2QL1, an E2 enzyme, is crucial for lysophagy by ubiquitinating lysosomal proteins following various types of lysosomal damage 335. However, how UBE2QL1 is recruited to damaged lysosomes has not been clearly illustrated. SCF^FBXO27^ (a glycoprotein-specific F-box protein, part of the SCF ubiquitin complex) can ubiquitinate glycoproteins like LAMP2 in damaged lysosomes. After treatment with LLOMe, FBXO27 on the cytoplasmic surface of membranes can bind to LAMP2, which is localized on the luminal side of lysosome membranes. Interestingly, endogenous P62 can colocalize with LAMP2 upon treatment with LLOMe. Hence, ubiquitinated lysosomes are degraded by autophagosomes 336. In addition to F-box protein 27 (FBXO27), the RING E3 ligase LRSAM1 is involved in the ubiquitination of lysosomes damaged by bacteria 337 (**Figure 8**).

Lysosomal membrane breakage and its removal by autophagy can also be monitored by galectins 331. Galectins are galactose-binding lectins which are normally localized in the cytoplasm and nucleus, whereas galactose-containing carbohydrate chains are enriched at the cell surface and at the luminal side of endosomes, lysosomes, and the Golgi apparatus 338. Endosomal or lysosomal membrane damage enables the recruitment of galectin-3 to the ruptured membrane 333, 339. In addition to galectin-3, galectin-1, -8 and -9 are also recruited to damaged membranes by bacterial invasion 340. In particular, galectin-8 binds to NDP52, an autophagy receptor with ubiquitin-binding activity 340, which suggests that galectin-1, -3, -8, and -9 are useful markers for damaged endomembranes. There are two mechanisms by which galectins can modulate downstream events. Galectin-3 recruits TRIM16, an atypical TRIM E3 ligase, which promotes K63-linked ubiquitination of ULK1 and ATG16L1. This suggests that a TRIM16-galectin-3 complex acts as a platform for autophagic initiation proteins that in turn induce phagophore formation 341. In contrast, galectin-8 directly binds the autophagic receptor NDP52 via a dedicated galectin-8 binding (GALBI) domain in NDP52, which recruits LC3-positive phagophores that then mediate lysophagy (**Figure 8**) 340. Although some galectins and autophagy receptors/adapters have been found to participate in the process of lysophagy, detailed molecular mechanisms and appropriate disease models of lysophagy are still limited.

Most selective autophagy processes share a common feature, which is that some proteins on the membrane of the damaged organelles are ubiquitinated 106, 342. Many ubiquitin-modified lysosomal proteins were identified by mass spectrometry when lysosomes were damaged 343, 344. P62 plays a major role in lysophagy due to its recruitment to damaged lysosomes. Moreover, depletion of P62 impairs lysosomal clearance 345. Following LLOMe-mediated lysosomal damage, K63- and K48-linked ubiquitin chains were shown to have functional and temporal differences 345, 346. Coinciding with the recruitment of the autophagy receptor P62 after lysosomal damage, K63-linked chains occur on lysosomes within an hour, while K48-linked conjugates peak at 2~4 hours after damage. Interestingly, K48-linked conjugates are targeted by the ubiquitin-directed p97 345. Impaired p97 function in lysophagy facilitates the accumulation of K48-linked conjugates and alleviates the degradation of damaged lysosomes. This means that K48-linked chains are prone to trigger the removal of some lysosomal proteins by p97, whereas K63-linked chains might tend to recruit autophagy receptors. Further studies of the role of ubiquitin, ubiquitin receptors, and the factors that modulate their activity will be required to enhance our understanding of lysophagy.

The pivotal role of lysosomes in cellular processes has been increasingly appreciated. Lysosomal storage diseases (LSDs) are characterized by disturbances of the activity of acid hydrolases, lysosomal membrane proteins and intralysosomal accumulation of substrates 347, 348. Various physiological functions may be affected by dysfunctional lysosomes resulting from LSDs. It has been uncovered that LSDs are associated with autoimmune phenomena 349, cholesterol homeostasis 350, cardiovascular diseases 351, 352, and NDDs 353. Some excellent recent reviews cover the current therapeutic strategies against LSDs 347, 354-356. Despite the progress made in understanding lysophagy and LSDs, we still cannot fully explain the individual pathologies. In addition, alteration in the activities of lysosomes is only one aspect of disease pathology, and combination therapies are encouraged to circumvent any unwanted side effects when targeting lysosomal diseases.

## Nucleophagy: selective degradation of genetic material

The nucleus is a double-membrane organelle containing most of cell's genetic material. It is found in eukaryotes but is absent in prokaryotes. The nucleus is composed of the nuclear envelope (a membrane structure that isolates the entire nuclear contents from the cytoplasm), the nuclear matrix, and the highly compact DNA molecules which are organized into chromosomes. The nucleus is essential for cell survival and proliferation, and regulates various cell activities by controlling gene expression.

Nucleophagy is the selective degradation of nuclear components including DNA, RNA, the nucleolus, nuclear proteins and the nuclear envelope. Nucleophagy can be divided into two forms: macronucleophagy and micronucleophagy (**Figure 9**). Macronucleophagy is the degradation of nuclear components as a selective form of macroautophagy, and the nuclear materials are engulfed by autophagosomes before fusing with lysosomes for degradation 357, 358. Micronucleophagy is the direct engulfment of nuclear material by the vacuole (in yeast), independent of autophagosome formation 359.

Nucleophagy in yeast is executed via micronucleophagy, which can be further classified into two types: piecemeal microautophagy of the nucleus (PMN) (**Figure 9A**) and late nucleophagy (LN) (**Figure 9B**). PMN is induced under nutrient-rich and early nitrogen starvation conditions and is characterized by the formation of direct contacts called nucleus-vacuole junctions (NVJs) between the nucleus and lytic vacuole. The nuclear membrane protrudes toward the vacuole and is then isolated from the nucleus and fuses with the vacuole for enzymatic degradation. Nucleus-vacuole junction protein 1 (Nvj1) and vacuolar protein 8 (Vac8) are required for PMN (**Figure 9A**). Nuclear envelope proteins, nuclear pore complexes, chromatin, and spindle pole bodies can be degraded by PMN 360, 361. Execution of PMN is independent of the core autophagy machinery including the Ub-like conjugation system and the Atg9 cycling system. Normally, LN is induced under long-term (20 ~ 24 hours) nitrogen starvation and is accompanied by structural changes in the nucleus. Unlike PMN, LN occurs without formation of NVJs (**Figure 9B**). Several components of the core autophagy machinery such as *Atg3*, *Atg4*, *Atg7* and *Atg8* are essential for the LN; however, *Atg6*, *Vps15* and *Vps34* are not required for this process. Defects in LN, caused by depletion of essential autophagy genes such as *Atg1* and *Atg8*, lead to abnormal nuclear structure, which implies that LN has a critical role in the maintenance of nuclear homeostasis 362.

Atg39 is required for degradation of both ER and nuclear components but is not essential for PMN. Very recent work has identified that Atg39 is a nucleophagy receptor in yeast. Atg39 localizes to the nuclear envelope and interacts with Atg11 to cause the sequestration of nuclear envelope-derived double-membrane vesicles. The substrates of Atg39-dependent nucleophagy include the nuclear envelope proteins Hmg1 and steroid receptor coactivator 1 (Src1) and the nucleolar protein 1 (Nop1) 164 (**Figure 9C**). These results indicate that a highly selective form of autophagy exists for nuclear components. However, it is not known whether the same mechanism exists in mammals because no Atg39 homologs have been identified. In general, nucleophagy plays a vital role in maintaining the health of yeast nuclei so that cells can survive under nutrient starvation.

Compared with the yeast system, nucleophagy is poorly understood in mammalian cells, although the connection between nuclear materials and the autophagic machinery has been constantly observed. Nucleophagy in mammal cells was first described in 2009. Perinuclear autophagosomes/autolysosomes containing nuclear components in nuclear envelopathies could be seen when the genes encoding A-type lamins (LMNA) and emerin (EMD) were mutated. Interestingly, giant autophagosomes/autolysosomes could also be seen even in wild-type cells to clean up wastes produced by nuclear damage 357. Many autophagic proteins, including LC3, ATG5, ATG7, NRBF2 and Beclin1, have been shown to localize to the nucleus, either under normal or stressed conditions 363-367. The autophagic adapter P62 is present both in the nucleus and in the cytoplasm. Under stress, P62 binds to ubiquitinated proteins and shuttles to the cytoplasm from the nucleus along with another autophagic receptor, ALFY 368. The nuclear lamina plays an important role in nuclear mechanical strength and higher-order chromatin organization, and hence modulates gene expression and silencing 369. Lamina protein lamin B1 is dramatically downregulated during oncogene-induced senescence instead of starvation or rapamycin treatment 370, 371. Notably, lamin B1 functions as an autophagy substrate which co-localizes and interacts with LC3 via its LIR in response to tumorigenic stress 372, 373. This interesting phenomenon suggests that selective degradation of the nuclear lamina by autophagy may restrain tumorigenesis. Micronuclei are fragments of nuclear material enclosed by nuclear membrane, which can be generated when cells are exposed to genotoxic compounds. Micronuclei have been shown to colocalize with LC3 and can be degraded by macroautophagy 374. Autophagic degradation of micronuclei has been regarded as a cellular protective mechanism to prevent chromosome instability during genetic toxicity. Currently, the mechanism underlying the selective macroautophagic degradation of nuclei is still elusive and the specific receptors for nucleophagy in mammals have not been identified.

Although accumulating evidence suggests that nucleophagy is implicated in human diseases, the relationship between nucleophagy and human pathological conditions is not well understood. Recent studies highlight the potential association between nucleophagy and diseases such as psoriasis, tumorigenesis and NDDs. Keratinization is a highly organized process in which keratinocytes lose their nuclei and become corneocytes. During this process, autophagy markers can be observed in the perinuclear region and nucleophagy may serve as a mechanism for removal of the nuclei. In psoriasis, autophagic structures can barely be observed, which suggests that there is a defect in nucleophagy 375. Oncogenic mutations and chromosome instability are markers of tumorigenesis. The autophagic degradation of nuclear protein lamin B1 can be triggered by KRAS mutation 373, which indicates that nucleophagy may help maintain chromosome stability by eliminating the mutated DNA. Dentatorubral-pallidoluysian atrophy (DRPLA) is a polyglutamine (polyQ) disease caused by the expansion of CAG repeats in the atropin-1 gene. In a mouse DRPLA model, nuclear degeneration, autophagy inhibition and activated nucleophagy-based lamin B1 degradation and excretion have been observed 376. The above-mentioned evidence reveals the association of nucleophagy with multiple disease conditions. However, the causative connection between nucleophagy dysregulation and the pathogenesis of human diseases has yet to be established.

In summary, nucleophagy is the process by which cells degrade and recycle the components of the nucleus. Although nucleophagy is a process that is conserved from yeast to mammals, its regulatory machinery in yeast and in mammals is distinct. Micronucleophagy is the major route for nuclear recycling in yeast, while mammalian cells rely on macronucleophagy for degradation of nuclear components. Nucleophagy has been regarded as a mechanism that protects cells against prolonged starvation, DNA damage and nuclear protein aggregations. However, there is also evidence suggesting that over-activation of nucleophagy leads to ageing-related phenotypes 377. Although the link between nucleophagy and human diseases has been revealed, more efforts are still needed to fully dissect the causative connection. Poor understanding of the regulatory mechanisms and a lack of animal models with nucleophagy defects are current obstacles for defining the role of nucleophagy in human diseases.

## Xenophagy and virophagy: specialized elimination of intracellular pathogens

Xenophagy plays a critical role in specialized elimination of intracellular pathogens (fungi, bacteria and viruses) 378. Analogous to aggrephagy or mitophagy, xenophagy utilizes autophagy receptors (P62, NDP52, OPTN and NBR1) to selective bring the cargo to the autophagosomal membrane 379. Interestingly, TAX1BP1, a paralogue of NDP52, functions as a novel autophagy receptor in myosin VI-mediated xenophagy. Depletion of TAX1BP1 causes an accumulation of ubiquitin-positive Salmonella 57 (**Figure 10**). The immune system plays a significant role in defending against pathogen invasion. Therefore, it is particularly important to clarify the regulation of autophagy on immunity. It has been uncovered that TAX1BP1 is necessary for early autophagy induction in LC3-dependent manner during T cell activation 380. Additionally, TRIM32-TAX1BP1-dependent selective autophagy degrades Toll-IL-1 receptor (TIR) domain-containing adapter-inducing IFN (TRIF) and effectively shuts down Toll-like receptor (TLR) 3/4-mediated innate immune and inflammatory responses 381.

As a subroutine of xenophagy, virophagy (the xenophagic disposal of viruses) has been described for different viral pathogens, including human immunodeficiency virus-1 (HIV-1) 382, 383 and herpes simplex virus-1 (HSV-1) 384. The coronavirus disease 2019 (COVID-19) pandemic is endangering individuals, governments and societies around the world 385. Hence, the possibility of virophagy exerting its antiviral role in COVID-19 is attractive from a therapeutic standpoint. Further study on the precise mechanisms of selective autophagy will greatly help us understand the pathogenesis of these diseases, which in turn will help us find prevention or treatment strategies.

## Conclusions

Autophagy plays an important role in cellular quality control and the clearance of misfolded protein aggregates and damaged organelles. Autophagy was originally thought to be a non-selective biological process within the cell. However, recent studies have shown that there are many types of selective autophagy in both yeast and higher eukaryotic cells, and selectivity of autophagy is probably a widespread phenomenon in cells.

While selective autophagy occurs in different forms, which correspond to the different cargoes, it shares most of its mechanisms with non-selective autophagy during autophagosome formation. The reason for the selectivity is that certain autophagy-related proteins or other effectors can specifically recognize receptor molecules on the cargoes (including organelles, pathogens, or large protein aggregates) and then initiate the formation of autophagosomes on the cargo surface 18, 21. In contrast, autophagy receptors and adapters do not seem to be required for nonselective autophagy. Although some cargo receptors directly bind their cargoes, in mammalian cells several receptor proteins recognize poly-ubiquitin chains attached to the surface of cargoes for selective autophagy. Therefore, the hypothesis of high-avidity interactions between cargo receptors and clustered target molecules should be tested for other cargo receptor proteins. Similarly, it will be interesting to see whether LIR-Atg8 interaction is conducive to processes other than autophagy, and to study whether LIR-Atg8 interaction can be used as drug target. Nevertheless, not all LIR-containing proteins are autophagy receptors. For instance, Atg3 and Atg4B, two LIR-containing proteins, are necessary for autophagosome formation 386, 387. Dishevelled (Dvl), an adapter protein in the Wnt signaling pathway, is degraded by the LIR motif 388. Other LIR-containing protein, like FYVE and coiled-coil domain-containing protein 1 (FYCO1), participate in the transportation and maturation of autophagosomes 389.

Even though substantial progress has been made in understanding the mechanism of selective autophagy, there are still unsolved problems about the identity of the new receptor for selective autophagy. It has been revealed that Atg11 acts as a scaffold protein by connecting cargo-receptor complexes and organelles in various types of selective autophagy in yeast 240. Receptor proteins like Atg19 390, Atg34 391, Atg32 41 and PpAtg30 255 have been identified from the Atg11-receptor-cargo complex. Therefore, it is necessary to identify mammalian homologues of Atg11 in order to discover new selective autophagy receptor proteins. Rigorous investigation is also required to clarify whether lipophagy is the same in all cell types. In addition, further researches are needed to resolve the controversy about the role of lipophagy in liver steatosis, and to find out whether the molecular mechanism of pexophagy is conserved in eukaryotes. Additionally, there is still a long way to go to identify the novel MAM proteins that mediate selective autophagy and the inactive proteasomes.

Available methods aimed at studying selective autophagy have promoted the vigorous development of this field. LysoIP proteomic analysis combined with sequence analysis and yeast two-hybrid screens is possible to identify new cargo receptors 246. Some biological protocols and excellent recent reviews cover many biochemical, cell biological and genetic methods, such as the alkaline phosphatase (ALP) assay, GFP-Atg8 assays, pH sensitive fluorescent reporter, fluorescence microscopy-based methods, the methylation-based M-Track assay, induced bypass (iPass), electron microscopy (EM), western blot-based assays, CRISPR/Cas9, *etc*, that are widely used in the study of selective autophagy 392-394. These tools allow researchers to solve problems in a variety of ways to confirm whether autophagy flow has changed.

So far, we have witnessed great progress in our understanding of selective autophagy. Here, we have discussed how specific stimuli or stresses are sensed by the cell and how selective autophagy receptors respond to these signals. We have also summarized the importance of post-translational modifications in regulating selective autophagy networks, including phosphorylation, dephosphorylation and ubiquitination. Since most selective autophagy is mediated by the interaction between receptors and ligands, how do post-translational and structural modifications affect the interaction between receptor and ligand? Similarly, kinase(s), E3-ubiquitin ligase(s), or phosphatase(s) should also be defined, which regulate the activity of selective autophagy receptors. In addition, the selectivity of autophagy receptors for cargo and their spatial and temporal function are important avenues for further study.

Selective autophagy is important for organelle quality control and for responding to nutrient supply. It is therefore closely related to cellular homeostasis and the development of diseases. Defective selective autophagy is harmful to cells, leading to cancer, neurodegeneration, metabolic diseases, heart dysfunction and inflammatory diseases 395, 396. It is extremely important to modulate selective autophagy in pathological states. Considering the specificity of receptors/adapters in different types of selective autophagy, it would be an interesting strategy to increase the activity of specific autophagy receptors/adapters, and promote the degradation of substrates at specific time points during the pathogenesis. Since the beginning of 1960s, the research on autophagy has almost increased exponentially. We believe that the ongoing research on selective autophagy will provide novel insights for answering these questions.

## Figures and Tables

**Table 1:** Selective autophagy receptors/adapters in yeast and mammals

Process	Mammals			Yeast		
	Receptors/Adapters	Positive regulation	Negative regulation	Receptors/Adapters	Positive regulation	Negative regulation
mitophagy	FUNDC1, NIX/BNIP3L, BNIP3, PHB2, NDP52, P62, OPTN, NBR1, TAX1BP1, Miro1, FKBP8, Ambra1, CL, ceramide	AMPK-mediated phosphorylation of ULK1, Phosphorylation of FUNDC1 (S17), Dephosphorylation of FUNDC1 (S13) by PGAM5, Phosphorylation of DRP1(S585), Phosphorylation of BNIP3(S17, S24), OA, CCCP, Hypoxia, Decreased expression of MFN2, ∆ψm↓, Phosphorylation of MFN2, TBK1, Iron depletion, Calcium dysregulation, IKKα, GSK-3β	CK2 and Src-mediated phosphorylation of FUNDC1(S13 and Y18), MUL1-meidated ubiquitination of ULK1, BCL2L1, MARCH5, microRNA-137, USP15, USP30, USP35, Dephosphorylation of ubiquitin, PTEN-L, Phosphorylation of DRP1(S637), Dephosphorylation of DRP1 (S616), MCL1	Atg32	Phosphorylation of Atg32 (S114), Atg11	―
ER-phagy	FAM134B, SEC62, RTN3, CCPG1, ATL3, TEX264, TRIM13, CALCOCO1.	BNIP3, P62, Beclin-1, VPS34, K63-linked Ub on TRIM13, VAPA , VAPB	―	Atg39, Atg40	Trs85, Lnp1	―
proteaphagy	P62	Proteasome inhibitors, Poly-ubiquitinated RPN1, RPN10 and RPN13, HSC73, ATP	―	―	Proteasome inhibitors, Cue5, Hsp42, Ubp3, Snx4, Snx41, Snx42	Blm10, Spg5, Rpn11, Nat3/Mdm20 complex
aggrephagy	P62, NBR1, ALFY , OPTN, Tollip	―	―	Cue5	―	―
ribophagy	NUFIP1	VPS34	―	―	Ubp3p/Bre5p, Rim15, Cdc48, Ufd3, arsenite	―
pexophagy	NBR1, P62, ACBD5	PEX2, ubiquitinated PEX5, PEX14, Atg7, peup1	USP30	Atg30, Atg36	Pex3, Atg37, Atg5, Atg7, Hrr25 kinase	―
lipophagy	ATGL, PNPLA8	Atg7, ATG14, AMPK, TFE3, TFEB, DNM2, Activation of PPARα, quercetin, flaviviruses, HTT	SOD1 deficiency, Depletion of Rab7, Activation of FXR	―	―	―
lysophagy	P62	LLOMe, UBE2QL1, SCF^FBXO27^, LRSAM1, TRIM16-galectin-3 complex, galectin-1/3/8/9	―	―	―	―
nucleophagy	―	Mutation of LMNA and EMD	―	Atg39	Atg1, Atg3, Atg4, Atg7, Atg8, Atg11	―
xenophagy	P62, NDP52, OPTN, NBR1, TAX1BP1	TRIM32	―	―	―	―

## Abbreviations

15-LOX: 15-lipoxygenase
Ac: acyl-CoA
ACBD5: acyl-CoA-binding domain containing protein 5
AD: Alzheimer's disease
AFLD: alcoholic fatty liver disease
AIM: Atg8-interacting motif
AKI: acute kidney injury
ALFY: autophagy-linked FYVE
ALP: alkaline phosphatase
ALS: Amyotrophic lateral sclerosis
Ambra1: Activating molecule in Beclin1-regulatedautophagy
AMPK: AMP-activated kinase
ATGL: adipocyte triglyceride lipase
ATGs: autophagy related genes
ATL3: atlastin 3
ATM: ataxia-telangiectasia mutated kinase
AUP1: ancient ubiquitous protein 1
BAT: brown adipose tissues
BCL2: B-cell lymphoma 2
BCL2L1: BCL2 like 1
BCL2L13: BCL2-like 13
BNIP3: Bcl-2/adenovirus E1B 19-kDa-interacting protein 3
CANX: calnexin
CVDs): cardiovascular pathologies
CCCP: carbonyl cyanide m-chlorophenylhydrazone
CCPG1: cell cycle progression 1
Cis1: cistrinin resistance protein
CK2: casein kinase 2
CL: cardiolipin
CMA: chaperone-mediated autophagy
CNS: central nervous system
COVID-19: coronavirus disease 2019
CP: core particle
CPS-6: CED-3 protease suppressor 6
DEHP: di-(2-ethylhexyl)phthalate
DENV: Dengue virus
DNM2: dynamin2
DRP1: dynamin-related protein 1
DRPLA: Dentatorubral-pallidoluysian atrophy
DUB: deubiquitinating enzyme
Dvl: Dishevelled
EBOV: Ebola virus
EM: electron microscopy
EMD: emerin
EPG5: Ectopic P-Granules Autophagy Protein 5 Homolog
ER: endoplasmic reticulum
ERAM: ER-to-autophagy membranes
ERAs: ER-containing autophagosomes
FAM134B: family with sequence similarity 134, member B
FAs: fatty acids
FBXO27: F-box protein 27
FFAs: free fatty acids
FIP200: FAK-family kinase interacting protein of 200 kDa
Fis1: fission 1
FKBP8: FKBP Prolyl Isomerase 8
FUNDC1: FUN14 domain containing 1
FXR: farnesoid X receptor
FYCO1: FYVE and coiled-coil domain-containing protein 1
G3BP: Ras-GTPase -activating protein (GAP)-binding protein
GALBI: galectin-8 binding
GIMs: GABARAP interaction motifs
HFD: high-fat diet
GSK-3β: glycogen synthase kinase-3 beta
HD: Huntington's disease
Hmg1: high mobility group 1
HOG: High Osmolarity Glycerol
HSAN I: autonomic neuropathy type I
HSC73: heat shock cognate protein 73
Hsp42: heat shock protein 42
HSV-1: herpes simplex virus-1
HTT: Huntingtin
IDR: intrinsically disordered region
IKKα: inhibitor of kappaB kinase alpha
iPass: induced bypass
IRE1α: inositol-requiring transmembrane kinase/endoribonuclease 1α
LAMP-2A: lysosome-associated membrane protein type 2A
LDs: lipid droplets
LIR: LC3-interacting region
LLO: listeriolysin O
LLOMe: L-leucyl-L-leucine methy ester
LMNA: lamins
LN: late nucleophagy
LONP: Lon protease
LRRK2: leucine rich repeat kinase 2
LRSAM1: Leucine-rich repeat and sterile alpha motif-containing protein 1
LSDs: Lysosomal storage diseases
Ltn1: listerin
LysoIP: immunopurification of lysosomes
MAM: mitochondrial-associated membrane
MARCH5: membrane-associated RING-CH 5
MCL1: myeloid cell leukemia 1
Mff: mitochondria fission factor
MFN2: mitofusin 2
MID49/51: mitochondrial dynamics protein of 49 and 51kDa
mPOS: mitochondrial precursor over-accumulation stress
mTORC1: mammalian target of rapamycin complex 1
mtROS: mitochondrial reactive oxygen species
mtUPR: nucleus include mitochondrial unfolded protein response
Miro1: Mitochondrial Rho GTPase 1
mitoCPR: mitochondrial compromised import response
MUL1: mitochondrial E3 ubiquitin protein ligase 1
NAFLD: non-alcoholic fatty liver disease
NBR1: neighbor of BRCA1 gene 1
NDDs: neurodegenerative diseases
NDP52: nuclear dot 52 kDa protein
NLRX1: NLR family member X1
Nop1: nucleolar protein 1
NUFIP1: nuclear fragile X mental retardation-interacting protein 1
Nvj1: Nucleus-vacuole junction protein 1
NVJs: nucleus-vacuole junctions
OA: oligomycin and antimycin-A
OPA1: optic atrophy type 1
OPTN: optineurin
PAS: phagophore assembly site/pre-autophagosomal-structure
PD: Parkinson's disease
Pdr3: pleiotropic drug resistance 3
peup1: peroxisome unusual positioning 1
PGAM5: phosphoglycerate mutase family member 5
PGC1α: peroxisome proliferator-activated receptor-γ coactivator-1α
PHB2: Prohibitin 2
PI3K: phosphoinositide 3-kinase
PINK1: PTEN-induced putative kinase 1
PKA: protein kinase A
PKC: Protein Kinase C
PLAA: phospholipase A 2-activating protein
PLEKHM1: Pleckstrin homology domain-containing protein family member 1
PLINs: Perilipins
PMN: piecemeal microautophagy of the nucleus
PMP70: 70-kDa peroxisomal membrane protein
PMs: peritoneal macrophages
PNPLA2: patatin-like phospholipase domain-containing protein 2
polyQ: polyglutamine
PPARα: peroxisome proliferator-activated receptor-α
*P. pastoris*: *Pichia pastoris*
PtdIns3P or PI3P: phosphatidylinositol 3-phosphate
PTEN-L: phosphatase and tensin homolog-long
RILP: Rab-interacting lysosomal protein
ROS: reactive oxygen species
RP: regulatory particle
RPC: receptor-protein complex
RPN10: regulatory particle non-ATPase 10
RTN3: reticulon 3
RTN3L: reticulon 3 long isoform
SAM: sorting and assembly machinery
*S. cerevisiae*: *Saccharomyces cerevisiae*
SCF: SKP1/CUL1/F-box protein
SEC62: translocation protein SEC62
SGs: stress granules
SIRT1: Sirtuin 1
SNAREs: soluble NSF attachment protein receptors
Snx: sorting nexin
SOD1: superoxide dismutase 1
Src1: steroid receptor coactivator 1
STING: stimulator of interferon genes
TAG: triacylglycerol
TAX1BP1: Tax1 Binding Protein 1
TBK1: TANK-binding kinase 1
TEX264: testis-expressed protein 264
TFE3: Transcription factor E3
TFEB: transcription factor-EB
TGs: triglycerides
TLR: Toll-like receptor
Tollip: toll interacting protein
TOR: target of rapamycin
TORC1: target of rapamycin complex 1
TRAF6: tumor necrosis factor receptor-associated factor 6
TRIF: Toll-IL-1 receptor (TIR) domain-containing adapter-inducing IFN
TRIM13: tripartite motif containing 13
TSC: tuberous sclerosis complex
UBA: ubiquitin-associated
UBE2QL1: ubiquitin conjugating enzyme E2Q family-like 1
Ubp3: Ubiquitin carboxyl-terminal hydrolase 3
UIM2: ubiquitin-interacting motif 2
UIR: UDS-interacting region
ULK1: unc-51 like autophagy activating kinase 1
UPR: unfolded protein response
UPRam: unfolded protein response activated by mistargeting of proteins
UPS: ubiquitin-proteasome system
USP15: Ubiquitin-specific protease 15
Vac8: vacuolar protein 8
VAPA: VAMP-associated A
VCP: valosin-containing protein
VDAC: voltage-dependent anion channel
VLDL: very low density lipoproteins
Vps21: vacuolar protein sorting 21
Vps34: vacuolar protein sorting 34
XBP1: X box binding protein 1
ZIKV: Zika virus
ZNHIT3: zinc finger HIT domain-containing protein 3

## References

[1] Kroemer G, Marino G, Levine B (2010). Autophagy and the integrated stress response. Mol Cell.

[2] Mizushima N, Komatsu M (2011). Autophagy: renovation of cells and tissues. Cell.

[3] Choi AM, Ryter SW, Levine B (2013). Autophagy in human health and disease. N Engl J Med.

[4] Feng Y, He D, Yao Z, Klionsky DJ (2014). The machinery of macroautophagy. Cell Res.

[5] Tanida I (2011). Autophagosome formation and molecular mechanism of autophagy. Antioxid Redox Signal.

[6] Tanida I (2011). Autophagy basics. Microbiol Immunol.

[7] Hyttinen JM, Niittykoski M, Salminen A, Kaarniranta K (2013). Maturation of autophagosomes and endosomes: a key role for Rab7. Biochim Biophys Acta.

[8] Wang Z, Miao G, Xue X, Guo X, Yuan C, Wang Z (2016). The Vici Syndrome Protein EPG5 Is a Rab7 Effector that Determines the Fusion Specificity of Autophagosomes with Late Endosomes/Lysosomes. Mol Cell.

[9] Zhao H, Zhao YG, Wang X, Xu L, Miao L, Feng D (2013). Mice deficient in Epg5 exhibit selective neuronal vulnerability to degeneration. J Cell Biol.

[10] Takats S, Pircs K, Nagy P, Varga A, Karpati M, Hegedus K (2014). Interaction of the HOPS complex with Syntaxin 17 mediates autophagosome clearance in Drosophila. Mol Biol Cell.

[11] Jiang P, Nishimura T, Sakamaki Y, Itakura E, Hatta T, Natsume T (2014). The HOPS complex mediates autophagosome-lysosome fusion through interaction with syntaxin 17. Mol Biol Cell.

[12] McEwan DG, Popovic D, Gubas A, Terawaki S, Suzuki H, Stadel D (2015). PLEKHM1 regulates autophagosome-lysosome fusion through HOPS complex and LC3/GABARAP proteins. Mol Cell.

[13] Ravikumar B, Sarkar S, Davies JE, Futter M, Garcia-Arencibia M, Green-Thompson ZW (2010). Regulation of mammalian autophagy in physiology and pathophysiology. Physiol Rev.

[14] Abada A, Levin-Zaidman S, Porat Z, Dadosh T, Elazar Z (2017). SNARE priming is essential for maturation of autophagosomes but not for their formation. Proc Natl Acad Sci U S A.

[15] Mizushima N (2007). Autophagy: process and function. Gene Dev.

[16] Glick D, Barth S, Macleod KF (2010). Autophagy: cellular and molecular mechanisms. J Pathol.

[17] Birgisdottir AB, Lamark T, Johansen T (2013). The LIR motif-crucial for selective autophagy. J Cell Sci.

[18] Zaffagnini G, Martens S (2016). Mechanisms of Selective Autophagy. J Mol Biol.

[19] Rogov VV, Stolz A, Ravichandran AC, Rios-Szwed DO, Suzuki H, Kniss A (2017). Structural and functional analysis of the GABARAP interaction motif (GIM). EMBO Rep.

[20] Noda NN, Kumeta H, Nakatogawa H, Satoo K, Adachi W, Ishii J (2008). Structural basis of target recognition by Atg8/LC3 during selective autophagy. Genes Cells.

[21] Johansen T, Lamark T (2011). Selective autophagy mediated by autophagic adapter proteins. Autophagy.

[22] Gatica D, Lahiri V, Klionsky DJ (2018). Cargo recognition and degradation by selective autophagy. Nat Cell Biol.

[23] Menzies FM, Fleming A, Caricasole A, Bento CF, Andrews SP, Ashkenazi A (2017). Autophagy and Neurodegeneration: Pathogenic Mechanisms and Therapeutic Opportunities. Neuron.

[24] Plaza-Zabala A, Sierra-Torre V, Sierra A (2017). Autophagy and Microglia: Novel Partners in Neurodegeneration and Aging. Int J Mol Sci.

[25] Lei Y, Zhang D, Yu J, Dong H, Zhang J, Yang S (2017). Targeting autophagy in cancer stem cells as an anticancer therapy. Cancer Lett.

[26] Guo JY, White E (2016). Autophagy, Metabolism, and Cancer. Cold Spring Harb Symp Quant Biol.

[27] Jacob JA, Salmani JM, Jiang Z, Feng L, Song J, Jia X (2017). Autophagy: An overview and its roles in cancer and obesity. Clin Chim Acta.

[28] Miettinen TP, Bjorklund M (2016). The mevalonate pathway as a metabolic requirement for autophagy-implications for growth control, proteostasis, and disease. Mol Cell Oncol.

[29] Wang F, Jia J, Rodrigues B (2017). Autophagy, Metabolic Disease, and Pathogenesis of Heart Dysfunction. Can J Cardiol.

[30] Zhang S, Lin X, Li G, Shen X, Niu D, Lu G (2017). Knockout of Eva1a leads to rapid development of heart failure by impairing autophagy. Cell Death Dis.

[31] Zhong Z, Sanchez-Lopez E, Karin M (2016). Autophagy, NLRP3 inflammasome and auto-inflammatory/immune diseases. Clin Exp Rheumatol.

[32] Suh HW, Kim JK, Kim TS, Jo EK (2017). New insights into vitamin D and autophagy in inflammatory bowel diseases. Curr Med Chem.

[33] Kim I, Rodriguez-Enriquez S, Lemasters JJ (2007). Selective degradation of mitochondria by mitophagy. Arch Biochem Biophys.

[34] Youle RJ, Narendra DP (2011). Mechanisms of mitophagy. Nat Rev Mol Cell Biol.

[35] Weidberg H, Amon A (2018). MitoCPR-A surveillance pathway that protects mitochondria in response to protein import stress. Science.

[36] Desai R, Campanella M (2018). MitoCPR: Meticulous Monitoring of Mitochondrial Proteostasis. Mol Cell.

[37] Tang MZ, Luo XL, Huang Z, Chen LX (2018). MitoCPR: a novel protective mechanism in response to mitochondrial protein import stress. Acta Bioch Bioph Sin.

[38] Pires J, Haynes CM (2018). Mitochondrial Biogenesis: MitoCPR Resuscitates Import-Defective Mitochondria. Curr Biol.

[39] Zhuang H, Tian W, Li W, Zhang X, Wang J, Yang Y (2016). Autophagic Cell Death and Apoptosis Jointly Mediate Cisatracurium Besylate-Induced Cell Injury. Int J Mol Sci.

[40] Okamoto K, Kondo-Okamoto N, Ohsumi Y (2009). Mitochondria-anchored receptor Atg32 mediates degradation of mitochondria via selective autophagy. Dev Cell.

[41] Kanki T, Wang K, Cao Y, Baba M, Klionsky DJ (2009). Atg32 Is a Mitochondrial Protein that Confers Selectivity during Mitophagy. Developmental Cell.

[42] Liu L, Feng D, Chen G, Chen M, Zheng Q, Song P (2012). Mitochondrial outer-membrane protein FUNDC1 mediates hypoxia-induced mitophagy in mammalian cells. Nat Cell Biol.

[43] Chen G, Han Z, Feng D, Chen Y, Chen L, Wu H (2014). A regulatory signaling loop comprising the PGAM5 phosphatase and CK2 controls receptor-mediated mitophagy. Mol Cell.

[44] Chen M, Chen Z, Wang Y, Tan Z, Zhu C, Li Y (2016). Mitophagy receptor FUNDC1 regulates mitochondrial dynamics and mitophagy. Autophagy.

[45] Novak I, Kirkin V, McEwan DG, Zhang J, Wild P, Rozenknop A (2010). Nix is a selective autophagy receptor for mitochondrial clearance. EMBO Rep.

[46] Schweers RL, Zhang J, Randall MS, Loyd MR, Li W, Dorsey FC (2007). NIX is required for programmed mitochondrial clearance during reticulocyte maturation. P Natl Acad Sci USA.

[47] Hanna RA, Quinsay MN, Orogo AM, Giang K, Rikka S, Gustafsson AB (2012). Microtubule-associated protein 1 light chain 3 (LC3) interacts with Bnip3 protein to selectively remove endoplasmic reticulum and mitochondria via autophagy. J Biol Chem.

[48] Zhu Y, Massen S, Terenzio M, Lang V, Chen-Lindner S, Eils R (2013). Modulation of serines 17 and 24 in the LC3-interacting region of Bnip3 determines pro-survival mitophagy versus apoptosis. J Biol Chem.

[49] Wei Y, Chiang WC, Sumpter R Jr, Mishra P, Levine B (2017). Prohibitin 2 Is an Inner Mitochondrial Membrane Mitophagy Receptor. Cell.

[50] Bhujabal Z, Birgisdottir AB, Sjottem E, Brenne HB, Overvatn A, Habisov S (2017). FKBP8 recruits LC3A to mediate Parkin-independent mitophagy. EMBO Rep.

[51] Lim GG, Lim KL (2017). Parkin-independent mitophagy-FKBP8 takes the stage. EMBO Rep.

[52] Wong YC, Holzbaur ELF (2014). Optineurin is an autophagy receptor for damaged mitochondria in parkin-mediated mitophagy that is disrupted by an ALS-linked mutation. P Natl Acad Sci USA.

[53] Lazarou M, Sliter DA, Kane LA, Sarraf SA, Wang C, Burman JL (2015). The ubiquitin kinase PINK1 recruits autophagy receptors to induce mitophagy. Nature.

[54] Gao F, Chen D, Si JM, Hu QS, Qin ZH, Fang M (2015). The mitochondrial protein BNIP3L is the substrate of PARK2 and mediates mitophagy in PINK1/PARK2 pathway. Hum Mol Genet.

[55] Shi J, Fung G, Deng H, Zhang J, Fiesel FC, Springer W (2015). NBR1 is dispensable for PARK2-mediated mitophagy regardless of the presence or absence of SQSTM1. Cell Death Dis.

[56] Cianfanelli V, De Zio D, Di Bartolomeo S, Nazio F, Strappazzon F, Cecconi F (2015). Ambra1 at a glance. J Cell Sci.

[57] Tumbarello DA, Manna PT, Allen M, Bycroft M, Arden SD, Kendrick-Jones J (2015). The Autophagy Receptor TAX1BP1 and the Molecular Motor Myosin VI Are Required for Clearance of Salmonella Typhimurium by Autophagy. PLoS Pathog.

[58] Chu CT, Ji J, Dagda RK, Jiang JF, Tyurina YY, Kapralov AA (2013). Cardiolipin externalization to the outer mitochondrial membrane acts as an elimination signal for mitophagy in neuronal cells. Nat Cell Biol.

[59] Chao H, Lin C, Zuo Q, Liu Y, Xiao M, Xu X (2019). Cardiolipin-Dependent Mitophagy Guides Outcome after Traumatic Brain Injury. J Neurosci.

[60] Sentelle RD, Senkal CE, Jiang W, Ponnusamy S, Gencer S, Selvam SP (2012). Ceramide targets autophagosomes to mitochondria and induces lethal mitophagy. Nat Chem Biol.

[61] Tian W, Li W, Chen Y, Yan Z, Huang X, Zhuang H (2015). Phosphorylation of ULK1 by AMPK regulates translocation of ULK1 to mitochondria and mitophagy. FEBS Lett.

[62] Li J, Qi W, Chen G, Feng D, Liu J, Ma B (2015). Mitochondrial outer-membrane E3 ligase MUL1 ubiquitinates ULK1 and regulates selenite-induced mitophagy. Autophagy.

[63] Wu H, Xue D, Chen G, Han Z, Huang L, Zhu C (2014). The BCL2L1 and PGAM5 axis defines hypoxia-induced receptor-mediated mitophagy. Autophagy.

[64] Chen Z, Liu L, Cheng Q, Li Y, Wu H, Zhang W (2017). Mitochondrial E3 ligase MARCH5 regulates FUNDC1 to fine-tune hypoxic mitophagy. EMBO Rep.

[65] Wu W, Li W, Chen H, Jiang L, Zhu R, Feng D (2016). FUNDC1 is a novel mitochondrial-associated-membrane (MAM) protein required for hypoxia-induced mitochondrial fission and mitophagy. Autophagy.

[66] Wu W, Lin C, Wu K, Jiang L, Wang X, Li W (2016). FUNDC1 regulates mitochondrial dynamics at the ER-mitochondrial contact site under hypoxic conditions. EMBO J.

[67] Zhang J, Ney PA (2009). Role of BNIP3 and NIX in cell death, autophagy, and mitophagy. Cell Death Differ.

[68] Zhang J, Loyd MR, Randall MS, Waddell MB, Kriwacki RW, Ney PA (2012). A short linear motif in BNIP3L (NIX) mediates mitochondrial clearance in reticulocytes. Autophagy.

[69] Li W, Zhang X, Zhuang H, Chen HG, Chen Y, Tian W (2014). MicroRNA-137 is a novel hypoxia-responsive microRNA that inhibits mitophagy via regulation of two mitophagy receptors FUNDC1 and NIX. J Biol Chem.

[70] Otsu K, Murakawa T, Yamaguchi O (2015). BCL2L13 is a mammalian homolog of the yeast mitophagy receptor Atg32. Autophagy.

[71] Aoki Y, Kanki T, Hirota Y, Kurihara Y, Saigusa T, Uchiumi T (2011). Phosphorylation of Serine 114 on Atg32 mediates mitophagy. Mol Biol Cell.

[72] Yamaguchi O, Murakawa T, Nishida K, Otsu K (2016). Receptor-mediated mitophagy. J Mol Cell Cardiol.

[73] Murakawa T, Yamaguchi O, Hashimoto A, Hikoso S, Takeda T, Oka T (2015). Bcl-2-like protein 13 is a mammalian Atg32 homologue that mediates mitophagy and mitochondrial fragmentation. Nat Commun.

[74] Yazdankhah M, Farioli-Vecchioli S, Tonchev AB, Stoykova A, Cecconi F (2014). The autophagy regulators Ambra1 and Beclin 1 are required for adult neurogenesis in the brain subventricular zone. Cell Death Dis.

[75] Van Humbeeck C, Cornelissen T, Vandenberghe W (2011). Ambra1: a Parkin-binding protein involved in mitophagy. Autophagy.

[76] Di Rita A, Peschiaroli A, P DA, Strobbe D, Hu Z, Gruber J (2018). HUWE1 E3 ligase promotes PINK1/PARKIN-independent mitophagy by regulating AMBRA1 activation via IKKalpha. Nat Commun.

[77] Strappazzon F, Di Rita A, Peschiaroli A, Leoncini PP, Locatelli F, Melino G (2020). HUWE1 controls MCL1 stability to unleash AMBRA1-induced mitophagy. Cell Death Differ.

[78] Merkwirth C, Langer T (2009). Prohibitin function within mitochondria: essential roles for cell proliferation and cristae morphogenesis. Biochim Biophys Acta.

[79] He L, Zhou H, Liu H, Qu H (2017). Prohibitin 2/PHB2 in Parkin-mediated mitophagy: a potential therapeutic target for mitochondrial diseases. Acta Biochim Biophys Sin (Shanghai).

[80] Zhou Q, Li H, Li H, Nakagawa A, Lin JL, Lee ES (2016). Mitochondrial endonuclease G mediates breakdown of paternal mitochondria upon fertilization. Science.

[81] Yan C, Gong L, Chen L, Xu M, Abou-Hamdan H, Tang M (2020). PHB2 (prohibitin 2) promotes PINK1-PRKN/Parkin-dependent mitophagy by the PARL-PGAM5-PINK1 axis. Autophagy.

[82] Li XX, Tsoi B, Li YF, Kurihara H, He RR (2015). Cardiolipin and Its Different Properties in Mitophagy and Apoptosis. J Histochem Cytochem.

[83] Betaneli V, Petrov EP, Schwille P (2012). The role of lipids in VDAC oligomerization. Biophys J.

[84] Shen ZN, Li YR, Gasparski AN, Abeliovich H, Greenberg ML (2017). Cardiolipin Regulates Mitophagy through the Protein Kinase C Pathway. Journal of Biological Chemistry.

[85] Jiang W, Ogretmen B (2013). Ceramide stress in survival versus lethal autophagy paradox: ceramide targets autophagosomes to mitochondria and induces lethal mitophagy. Autophagy.

[86] Whitworth AJ, Pallanck LJ (2009). The PINK1/Parkin pathway: a mitochondrial quality control system?. J Bioenerg Biomembr.

[87] Ashrafi G, Schwarz TL (2013). The pathways of mitophagy for quality control and clearance of mitochondria. Cell Death Differ.

[88] Wang Y, Nartiss Y, Steipe B, McQuibban GA, Kim PK (2012). ROS-induced mitochondrial depolarization initiates PARK2/PARKIN-dependent mitochondrial degradation by autophagy. Autophagy.

[89] Burman JL, Pickles S, Wang CX, Sekine S, Vargas JNS, Zhang Z (2017). Mitochondrial fission facilitates the selective mitophagy of protein aggregates. J Cell Biol.

[90] Narendra DP, Jin SM, Tanaka A, Suen DF, Gautier CA, Shen J (2010). PINK1 is selectively stabilized on impaired mitochondria to activate Parkin. PLoS Biol.

[91] Vives-Bauza C, Zhou C, Huang Y, Cui M, de Vries RL, Kim J (2010). PINK1-dependent recruitment of Parkin to mitochondria in mitophagy. Proc Natl Acad Sci U S A.

[92] Kondapalli C, Kazlauskaite A, Zhang N, Woodroof HI, Campbell DG, Gourlay R (2012). PINK1 is activated by mitochondrial membrane potential depolarization and stimulates Parkin E3 ligase activity by phosphorylating Serine 65. Open Biol.

[93] Kane LA, Lazarou M, Fogel AI, Li Y, Yamano K, Sarraf SA (2014). PINK1 phosphorylates ubiquitin to activate Parkin E3 ubiquitin ligase activity. J Cell Biol.

[94] Okatsu K, Kimura M, Oka T, Tanaka K, Matsuda N (2015). Unconventional PINK1 localization to the outer membrane of depolarized mitochondria drives Parkin recruitment. J Cell Sci.

[95] Okatsu K, Koyano F, Kimura M, Kosako H, Saeki Y, Tanaka K (2015). Phosphorylated ubiquitin chain is the genuine Parkin receptor. J Cell Biol.

[96] Wauer T, Simicek M, Schubert A, Komander D (2015). Mechanism of phospho-ubiquitin-induced PARKIN activation. Nature.

[97] Wauer T, Swatek KN, Wagstaff JL, Gladkova C, Pruneda JN, Michel MA (2015). Ubiquitin Ser65 phosphorylation affects ubiquitin structure, chain assembly and hydrolysis. EMBO J.

[98] Koyano F, Okatsu K, Kosako H, Tamura Y, Go E, Kimura M (2014). Ubiquitin is phosphorylated by PINK1 to activate parkin. Nature.

[99] Imai Y, Soda M, Inoue H, Hattori N, Mizuno Y, Takahashi R (2001). An unfolded putative transmembrane polypeptide, which can lead to endoplasmic reticulum stress, is a substrate of Parkin. Cell.

[100] Imai Y, Soda M, Hatakeyama S, Akagi T, Hashikawa T, Nakayama KI (2002). CHIP is associated with Parkin, a gene responsible for familial Parkinson's disease, and enhances its ubiquitin ligase activity. Mol Cell.

[101] Fallon L, Belanger CM, Corera AT, Kontogiannea M, Regan-Klapisz E, Moreau F (2006). A regulated interaction with the UIM protein Eps15 implicates parkin in EGF receptor trafficking and PI(3)K-Akt signalling. Nat Cell Biol.

[102] Trempe JF, Chen CX, Grenier K, Camacho EM, Kozlov G, McPherson PS (2009). SH3 domains from a subset of BAR proteins define a Ubl-binding domain and implicate parkin in synaptic ubiquitination. Mol Cell.

[103] Sarraf SA, Raman M, Guarani-Pereira V, Sowa ME, Huttlin EL, Gygi SP (2013). Landscape of the PARKIN-dependent ubiquitylome in response to mitochondrial depolarization. Nature.

[104] Durcan TM, Fon EA (2015). The three 'P's of mitophagy: PARKIN, PINK1, and post-translational modifications. Genes Dev.

[105] Jian FL, Chen D, Chen L, Yan CJ, Lu B, Zhu YS (2018). Sam50 Regulates PINK1-Parkin-Mediated Mitophagy by Controlling PINK1 Stability and Mitochondrial Morphology. Cell Rep.

[106] Stolz A, Ernst A, Dikic I (2014). Cargo recognition and trafficking in selective autophagy. Nature Cell Biology.

[107] Rogov V, Dotsch V, Johansen T, Kirkin V (2014). Interactions between Autophagy Receptors and Ubiquitin-like Proteins Form the Molecular Basis for Selective Autophagy. Mol Cell.

[108] Heo JM, Ordureau A, Paulo JA, Rinehart J, Harper JW (2015). The PINK1-PARKIN Mitochondrial Ubiquitylation Pathway Drives a Program of OPTN/NDP52 Recruitment and TBK1 Activation to Promote Mitophagy. Mol Cell.

[109] Strappazzon F, Nazio F, Corrado M, Cianfanelli V, Romagnoli A, Fimia GM (2015). AMBRA1 is able to induce mitophagy via LC3 binding, regardless of PARKIN and p62/SQSTM1. Cell Death Differ.

[110] Van Humbeeck C, Cornelissen T, Hofkens H, Mandemakers W, Gevaert K, De Strooper B (2011). Parkin interacts with Ambra1 to induce mitophagy. J Neurosci.

[111] Nazio F, Strappazzon F, Antonioli M, Bielli P, Cianfanelli V, Bordi M (2013). mTOR inhibits autophagy by controlling ULK1 ubiquitylation, self-association and function through AMBRA1 and TRAF6. Nat Cell Biol.

[112] Ordureau A, Sarraf SA, Duda DM, Heo JM, Jedrychowski MP, Sviderskiy VO (2014). Quantitative proteomics reveal a feedforward mechanism for mitochondrial PARKIN translocation and ubiquitin chain synthesis. Mol Cell.

[113] Ordureau A, Paulo JA, Zhang W, Ahfeldt T, Zhang J, Cohn EF (2018). Dynamics of PARKIN-Dependent Mitochondrial Ubiquitylation in Induced Neurons and Model Systems Revealed by Digital Snapshot Proteomics. Mol Cell.

[114] Ying H, Yue BY (2016). Optineurin: The autophagy connection. Exp Eye Res.

[115] Richter B, Sliter DA, Herhaus L, Stolz A, Wang CX, Beli P (2016). Phosphorylation of OPTN by TBK1 enhances its binding to Ub chains and promotes selective autophagy of damaged mitochondria. P Natl Acad Sci USA.

[116] Padman BS, Nguyen TN, Uoselis L, Skulsuppaisarn M, Nguyen LK, Lazarou M (2019). LC3/GABARAPs drive ubiquitin-independent recruitment of Optineurin and NDP52 to amplify mitophagy. Nat Commun.

[117] Yamada T, Dawson TM, Yanagawa T, Iijima M, Sesaki H (2019). SQSTM1/p62 promotes mitochondrial ubiquitination independently of PINK1 and PRKN/parkin in mitophagy. Autophagy.

[118] Zhong Z, Umemura A, Sanchez-Lopez E, Liang S, Shalapour S, Wong J (2016). NF-kappaB Restricts Inflammasome Activation via Elimination of Damaged Mitochondria. Cell.

[119] Cornelissen T, Haddad D, Wauters F, Van Humbeeck C, Mandemakers W, Koentjoro B (2014). The deubiquitinase USP15 antagonizes Parkin-mediated mitochondrial ubiquitination and mitophagy. Hum Mol Genet.

[120] Pickrell AM, Youle RJ (2015). The Roles of PINK1, Parkin, and Mitochondrial Fidelity in Parkinson's Disease. Neuron.

[121] Bingol B, Tea JS, Phu L, Reichelt M, Bakalarski CE, Song Q (2014). The mitochondrial deubiquitinase USP30 opposes parkin-mediated mitophagy. Nature.

[122] Wang Y, Serricchio M, Jauregui M, Shanbhag R, Stoltz T, Di Paolo CT (2015). Deubiquitinating enzymes regulate PARK2-mediated mitophagy. Autophagy.

[123] Eiyama A, Okamoto K (2015). PINK1/Parkin-mediated mitophagy in mammalian cells. Curr Opin Cell Biol.

[124] Wang L, Cho YL, Tang Y, Wang J, Park JE, Wu Y (2018). PTEN-L is a novel protein phosphatase for ubiquitin dephosphorylation to inhibit PINK1-Parkin-mediated mitophagy. Cell Res.

[125] Gouspillou G, Godin R, Piquereau J, Picard M, Mofarrahi M, Mathew J (2018). Protective role of Parkin in skeletal muscle contractile and mitochondrial function. J Physiol.

[126] Gegg ME, Cooper JM, Chau KY, Rojo M, Schapira AHV, Taanman JW (2010). Mitofusin 1 and mitofusin 2 are ubiquitinated in a PINK1/parkin-dependent manner upon induction of mitophagy. Hum Mol Genet.

[127] Glauser L, Sonnay S, Stafa K, Moore DJ (2011). Parkin promotes the ubiquitination and degradation of the mitochondrial fusion factor mitofusin 1. J Neurochem.

[128] Chen Y, Dorn GW 2nd (2013). PINK1-phosphorylated mitofusin 2 is a Parkin receptor for culling damaged mitochondria. Science.

[129] Wang X (2017). Destructive cellular paths underlying familial and sporadic Parkinson disease converge on mitophagy. Autophagy.

[130] Lahiri V, Klionsky DJ (2017). Functional impairment in RHOT1/Miro1 degradation and mitophagy is a shared feature in familial and sporadic Parkinson disease. Autophagy.

[131] Hsieh CH, Shaltouki A, Gonzalez AE, Bettencourt da Cruz A, Burbulla LF, St Lawrence E (2016). Functional Impairment in Miro Degradation and Mitophagy Is a Shared Feature in Familial and Sporadic Parkinson's Disease. Cell Stem Cell.

[132] Sliter DA, Martinez J, Hao L, Chen X, Sun N, Fischer TD (2018). Parkin and PINK1 mitigate STING-induced inflammation. Nature.

[133] McWilliams TG, Prescott AR, Montava-Garriga L, Ball G, Singh F, Barini E (2018). Basal Mitophagy Occurs Independently of PINK1 in Mouse Tissues of High Metabolic Demand. Cell Metab.

[134] Ni HM, Williams JA, Ding WX (2015). Mitochondrial dynamics and mitochondrial quality control. Redox Biol.

[135] Osellame LD, Singh AP, Stroud DA, Palmer CS, Stojanovski D, Ramachandran R (2016). Cooperative and independent roles of the Drp1 adaptors Mff, MiD49 and MiD51 in mitochondrial fission. J Cell Sci.

[136] Kageyama Y, Hoshijima M, Seo K, Bedja D, Sysa-Shah P, Andrabi SA (2014). Parkin-independent mitophagy requires Drp1 and maintains the integrity of mammalian heart and brain. EMBO J.

[137] Senft D, Ronai ZA (2016). Regulators of mitochondrial dynamics in cancer. Curr Opin Cell Biol.

[138] Gomes LC, Di Benedetto G, Scorrano L (2011). During autophagy mitochondria elongate, are spared from degradation and sustain cell viability. Nat Cell Biol.

[139] Rambold AS, Kostelecky B, Elia N, Lippincott-Schwartz J (2011). Tubular network formation protects mitochondria from autophagosomal degradation during nutrient starvation. Proc Natl Acad Sci U S A.

[140] Wang ZG, Jiang H, Chen S, Du FH, Wang XD (2012). The Mitochondrial Phosphatase PGAM5 Functions at the Convergence Point of Multiple Necrotic Death Pathways. Cell.

[141] Taguchi N, Ishihara N, Jofuku A, Oka T, Mihara K (2007). Mitotic phosphorylation of dynamin-related GTPase Drp1 participates in mitochondrial fission. J Biol Chem.

[142] Chen G, Kroemer G, Kepp O (2020). Mitophagy: An Emerging Role in Aging and Age-Associated Diseases. Front Cell Dev Biol.

[143] Shirakabe A, Zhai P, Ikeda Y, Saito T, Maejima Y, Hsu CP (2016). Drp1-Dependent Mitochondrial Autophagy Plays a Protective Role Against Pressure Overload-Induced Mitochondrial Dysfunction and Heart Failure. Circulation.

[144] Zhao C, Chen Z, Qi J, Duan S, Huang Z, Zhang C (2017). Drp1-dependent mitophagy protects against cisplatin-induced apoptosis of renal tubular epithelial cells by improving mitochondrial function. Oncotarget.

[145] Vantaggiato C, Castelli M, Giovarelli M, Orso G, Bassi MT, Clementi E (2019). The Fine Tuning of Drp1-Dependent Mitochondrial Remodeling and Autophagy Controls Neuronal Differentiation. Front Cell Neurosci.

[146] West AP, Shadel GS, Ghosh S (2011). Mitochondria in innate immune responses. Nat Rev Immunol.

[147] West AP, Brodsky IE, Rahner C, Woo DK, Erdjument-Bromage H, Tempst P (2011). TLR signalling augments macrophage bactericidal activity through mitochondrial ROS. Nature.

[148] Palikaras K, Lionaki E, Tavernarakis N (2018). Mechanisms of mitophagy in cellular homeostasis, physiology and pathology. Nat Cell Biol.

[149] Zhang YF, Yao YK, Qiu XX, Wang GD, Hu Z, Chen SY (2019). Listeria hijacks host mitophagy through a novel mitophagy receptor to evade killing. Nat Immunol.

[150] Wang M, Kaufman RJ (2016). Protein misfolding in the endoplasmic reticulum as a conduit to human disease. Nature.

[151] Yin Y, Sun G, Li E, Kiselyov K, Sun D (2017). ER stress and impaired autophagy flux in neuronal degeneration and brain injury. Ageing Res Rev.

[152] Zhang Z, Gao W, Zhou L, Chen Y, Qin S, Zhang L (2019). Repurposing Brigatinib for the Treatment of Colorectal Cancer Based on Inhibition of ER-phagy. Theranostics.

[153] Nie T, Yang S, Ma H, Zhang L, Lu F, Tao K (2016). Regulation of ER stress-induced autophagy by GSK3beta-TIP60-ULK1 pathway. Cell Death Dis.

[154] Wang T, Yuan Y, Zou H, Yang J, Zhao S, Ma Y (2016). The ER stress regulator Bip mediates cadmium-induced autophagy and neuronal senescence. Sci Rep.

[155] Bernales S, Schuck S, Walter P (2007). ER-phagy: selective autophagy of the endoplasmic reticulum. Autophagy.

[156] Lipatova Z, Segev N (2015). A Role for Macro-ER-Phagy in ER Quality Control. Plos Genet.

[157] Munakata N, Klionsky DJ (2010). "Autophagy suite": Atg9 cycling in the cytoplasm to vacuole targeting pathway. Autophagy.

[158] Sclafani A, Chen S, Rivera-Molina F, Reinisch K, Novick P, Ferro-Novick S (2010). Establishing a role for the GTPase Ypt1p at the late Golgi. Traffic.

[159] Lipatova Z, Belogortseva N, Zhang XQ, Kim J, Taussig D, Segev N (2012). Regulation of selective autophagy onset by a Ypt/Rab GTPase module. Proc Natl Acad Sci U S A.

[160] Lipatova Z, Segev N (2012). A Ypt/Rab GTPase module makes a PAS. Autophagy.

[161] Lipatova Z, Shah AH, Kim JJ, Mulholland JW, Segev N (2013). Regulation of ER-phagy by a Ypt/Rab GTPase module. Mol Biol Cell.

[162] Bernales S, McDonald KL, Walter P (2006). Autophagy counterbalances endoplasmic reticulum expansion during the unfolded protein response. PLoS Biol.

[163] Schuck S, Gallagher CM, Walter P (2014). ER-phagy mediates selective degradation of endoplasmic reticulum independently of the core autophagy machinery. J Cell Sci.

[164] Mochida K, Oikawa Y, Kimura Y, Kirisako H, Hirano H, Ohsumi Y (2015). Receptor-mediated selective autophagy degrades the endoplasmic reticulum and the nucleus. Nature.

[165] Chen S, Cui Y, Parashar S, Novick PJ, Ferro-Novick S (2018). ER-phagy requires Lnp1, a protein that stabilizes rearrangements of the ER network. Proc Natl Acad Sci U S A.

[166] Grumati P, Dikic I, Stolz A (2018). ER-phagy at a glance. J Cell Sci.

[167] Chen QZ, Xiao Y, Chai PY, Zheng PL, Teng JL, Chen JG (2019). ATL3 Is a Tubular ER-Phagy Receptor for GABARAP-Mediated Selective Autophagy. Curr Biol.

[168] Chino H, Hatta T, Natsume T, Mizushima N (2019). Intrinsically Disordered Protein TEX264 Mediates ER-phagy. Mol Cell.

[169] Ji CH, Kim HY, Heo AJ, Lee SH, Lee MJ, Bin Kim S (2019). The N-Degron Pathway Mediates ER-phagy. Mol Cell.

[170] Khaminets A, Heinrich T, Mari M, Grumati P, Huebner AK, Akutsu M (2015). Regulation of endoplasmic reticulum turnover by selective autophagy. Nature.

[171] Lennemann NJ, Coyne CB (2017). Dengue and Zika viruses subvert reticulophagy by NS2B3-mediated cleavage of FAM134B. Autophagy.

[172] Chiramel AI, Dougherty JD, Nair V, Robertson SJ, Best SM (2016). FAM134B, the Selective Autophagy Receptor for Endoplasmic Reticulum Turnover, Inhibits Replication of Ebola Virus Strains Makona and Mayinga. J Infect Dis.

[173] Chiramel AI, Best SM (2018). Role of autophagy in Zika virus infection and pathogenesis. Virus Res.

[174] Greiner M, Kreutzer B, Jung V, Grobholz R, Hasenfus A, Stohr RF (2011). Silencing of the SEC62 gene inhibits migratory and invasive potential of various tumor cells. Int J Cancer.

[175] Linxweiler M, Linxweiler J, Barth M, Benedix J, Jung V, Kim YJ (2012). Sec62 bridges the gap from 3q amplification to molecular cell biology in non-small cell lung cancer. Am J Pathol.

[176] Weng L, Du J, Zhou Q, Cheng B, Li J, Zhang D (2012). Identification of cyclin B1 and Sec62 as biomarkers for recurrence in patients with HBV-related hepatocellular carcinoma after surgical resection. Mol Cancer.

[177] Hagerstrand D, Tong A, Schumacher SE, Ilic N, Shen RR, Cheung HW (2013). Systematic interrogation of 3q26 identifies TLOC1 and SKIL as cancer drivers. Cancer Discov.

[178] Fumagalli F, Noack J, Bergmann TJ, Presmanes EC, Pisoni GB, Fasana E (2016). Translocon component Sec62 acts in endoplasmic reticulum turnover during stress recovery. Nat Cell Biol.

[179] Bergmann TJ, Fumagalli F, Loi M, Molinari M (2017). Role of SEC62 in ER maintenance: A link with ER stress tolerance in SEC62-overexpressing tumors?. Mol Cell Oncol.

[180] Grumati P, Morozzi G, Holper S, Mari M, Harwardt MI, Yan R (2017). Full length RTN3 regulates turnover of tubular endoplasmic reticulum via selective autophagy. Elife.

[181] Smith MD, Harley ME, Kemp AJ, Wills J, Lee M, Arends M (2018). CCPG1 Is a Non-canonical Autophagy Cargo Receptor Essential for ER-Phagy and Pancreatic ER Proteostasis. Dev Cell.

[182] Mizushima N (2018). A Dual Binding Receptor for ER-phagy. Dev Cell.

[183] An H, Ordureau A, Paulo JA, Shoemaker CJ, Denic V, Harper JW (2019). TEX264 Is an Endoplasmic Reticulum-Resident ATG8-Interacting Protein Critical for ER Remodeling during Nutrient Stress. Mol Cell.

[184] Glick D, Zhang WS, Beaton M, Marsboom G, Gruber M, Simon MC (2012). BNip3 Regulates Mitochondrial Function and Lipid Metabolism in the Liver. Mol Cell Biol.

[185] Yang H, Ni HM, Guo FL, Ding YF, Shi YH, Lahiri P (2016). Sequestosome 1/p62 Protein Is Associated with Autophagic Removal of Excess Hepatic Endoplasmic Reticulum in Mice. J Biol Chem.

[186] Cribb AE, Peyrou M, Muruganandan S, Schneider L (2005). The endoplasmic reticulum in xenobiotic toxicity. Drug Metab Rev.

[187] Park C, Cuervo AM (2013). Selective Autophagy: Talking with the UPS. Cell Biochem Biophys.

[188] Manley S, Williams JA, Ding WX (2013). Role of p62/SQSTM1 in liver physiology and pathogenesis. Exp Biol Med.

[189] Nthiga TM, Kumar Shrestha B, Sjottem E, Bruun JA, Bowitz Larsen K, Bhujabal Z (2020). CALCOCO1 acts with VAMP-associated proteins to mediate ER-phagy. EMBO J.

[190] Hosoi T, Ozawa K (2009). Endoplasmic reticulum stress in disease: mechanisms and therapeutic opportunities. Clin Sci (Lond).

[191] Hubner CA, Dikic I (2020). ER-phagy and human diseases. Cell Death Differ.

[192] Du J, Wang XN, Miereles C, Bailey JL, Debigare R, Zheng B (2004). Activation of caspase-3 is an initial step triggering accelerated muscle proteolysis in catabolic conditions. J Clin Invest.

[193] Sandri M (2013). Protein breakdown in muscle wasting: role of autophagy-lysosome and ubiquitin-proteasome. Int J Biochem Cell Biol.

[194] Cuervo AM, Palmer A, Rivett AJ, Knecht E (1995). Degradation of proteasomes by lysosomes in rat liver. Eur J Biochem.

[195] Marshall RS, Li FQ, Gemperline DC, Book AJ, Vierstra RD (2015). Autophagic Degradation of the 26S Proteasome Is Mediated by the Dual ATG8/Ubiquitin Receptor RPN10 in Arabidopsis. Mol Cell.

[196] Bartel B (2015). Proteaphagy-Selective Autophagy of Inactive Proteasomes. Mol Cell.

[197] Wen X, Klionsky DJ (2016). The proteasome subunit RPN10 functions as a specific receptor for degradation of the 26S proteasome by macroautophagy in Arabidopsis. Autophagy.

[198] Marshall RS, McLoughlin F, Vierstra RD (2016). Autophagic Turnover of Inactive 26S Proteasomes in Yeast Is Directed by the Ubiquitin Receptor Cue5 and the Hsp42 Chaperone. Cell Rep.

[199] Marshall RS, Vierstra RD (2015). Eat or be eaten: The autophagic plight of inactive 26S proteasomes. Autophagy.

[200] Husnjak K, Dikic I (2012). Ubiquitin-Binding Proteins: Decoders of Ubiquitin-Mediated Cellular Functions. Annu Rev Biochem.

[201] Hamazaki J, Hirayama S, Murata S (2015). Redundant Roles of Rpn10 and Rpn13 in Recognition of Ubiquitinated Proteins and Cellular Homeostasis. Plos Genet.

[202] Noda NN, Ohsumi Y, Inagaki F (2010). Atg8-family interacting motif crucial for selective autophagy. FEBS Lett.

[203] Cohen-Kaplan V, Livneh I, Avni N, Fabre B, Ziv T, Kwon YT (2016). p62- and ubiquitin-dependent stress-induced autophagy of the mammalian 26S proteasome. Proc Natl Acad Sci U S A.

[204] Marshall RS, Vierstra RD (2019). Dynamic Regulation of the 26S Proteasome: From Synthesis to Degradation. Front Mol Biosci.

[205] Waite KA, De-La Mota-Peynado A, Vontz G, Roelofs J (2016). Starvation Induces Proteasome Autophagy with Different Pathways for Core and Regulatory Particles. J Biol Chem.

[206] Nemec AA, Howell LA, Peterson AK, Murray MA, Tomko RJ Jr (2017). Autophagic clearance of proteasomes in yeast requires the conserved sorting nexin Snx4. J Biol Chem.

[207] Kraft C, Deplazes A, Sohrmann M, Peter M (2008). Mature ribosomes are selectively degraded upon starvation by an autophagy pathway requiring the Ubp3p/Bre5p ubiquitin protease. Nat Cell Biol.

[208] Beese CJ, Brynjolfsdottir SH, Frankel LB (2020). Selective Autophagy of the Protein Homeostasis Machinery: Ribophagy, Proteaphagy and ER-Phagy. Front Cell Dev Biol.

[209] Marshall RS, Vierstra RD (2018). To save or degrade: balancing proteasome homeostasis to maximize cell survival. Autophagy.

[210] Overbye A, Fengsrud M, Seglen PO (2007). Proteomic analysis of membrane-associated proteins from rat liver autophagosomes. Autophagy.

[211] Lu K, Psakhye I, Jentsch S (2014). Autophagic clearance of polyQ proteins mediated by ubiquitin-Atg8 adaptors of the conserved CUET protein family. Cell.

[212] Lu K, Psakhye I, Jentsch S (2014). A new class of ubiquitin-Atg8 receptors involved in selective autophagy and polyQ protein clearance. Autophagy.

[213] Filimonenko M, Isakson P, Finley KD, Anderson M, Jeong H, Melia TJ (2010). The selective macroautophagic degradation of aggregated proteins requires the PI3P-binding protein Alfy. Mol Cell.

[214] Lamark T, Johansen T (2012). Aggrephagy: selective disposal of protein aggregates by macroautophagy. Int J Cell Biol.

[215] van Blitterswijk M, van Vught PWJ, van Es MA, Schelhaas HJ, van der Kooi AJ, de Visser M (2012). Novel optineurin mutations in sporadic amyotrophic lateral sclerosis patients. Neurobiol Aging.

[216] Maruyama H, Morino H, Ito H, Izumi Y, Kato H, Watanabe Y (2010). Mutations of optineurin in amyotrophic lateral sclerosis. Nature.

[217] Yamamoto A, Simonsen A (2011). The elimination of accumulated and aggregated proteins: a role for aggrephagy in neurodegeneration. Neurobiol Dis.

[218] McEwan DG, Dikic I (2011). The Three Musketeers of Autophagy: phosphorylation, ubiquitylation and acetylation. Trends Cell Biol.

[219] Conway O, Akpinar HA, Rogov VV, Kirkin V (2020). Selective Autophagy Receptors in Neuronal Health and Disease. J Mol Biol.

[220] Vicencio E, Beltran S, Labrador L, Manque P, Nassif M, Woehlbier U (2020). Implications of Selective Autophagy Dysfunction for ALS Pathology. Cells.

[221] Eskelinen EL, Reggiori F, Baba M, Kovacs AL, Seglen PO (2011). Seeing is believing: the impact of electron microscopy on autophagy research. Autophagy.

[222] Carloni S, Albertini MC, Galluzzi L, Buonocore G, Proietti F, Balduini W (2014). Increased autophagy reduces endoplasmic reticulum stress after neonatal hypoxia-ischemia: role of protein synthesis and autophagic pathways. Exp Neurol.

[223] Lardeux BR, Heydrick SJ, Mortimore GE (1987). RNA degradation in perfused rat liver as determined from the release of [14C]cytidine. J Biol Chem.

[224] Boglev Y, Badrock AP, Trotter AJ, Du Q, Richardson EJ, Parslow AC (2013). Autophagy induction is a Tor- and Tp53-independent cell survival response in a zebrafish model of disrupted ribosome biogenesis. PLoS Genet.

[225] Ossareh-Nazari B, Nino CA, Bengtson MH, Lee JW, Joazeiro CA, Dargemont C (2014). Ubiquitylation by the Ltn1 E3 ligase protects 60S ribosomes from starvation-induced selective autophagy. J Cell Biol.

[226] Muller M, Kotter P, Behrendt C, Walter E, Scheckhuber CQ, Entian KD (2015). Synthetic quantitative array technology identifies the Ubp3-Bre5 deubiquitinase complex as a negative regulator of mitophagy. Cell Rep.

[227] Soncini C, Berdo I, Draetta G (2001). Ras-GAP SH3 domain binding protein (G3BP) is a modulator of USP10, a novel human ubiquitin specific protease. Oncogene.

[228] Buchan JR, Kolaitis RM, Taylor JP, Parker R (2013). Eukaryotic Stress Granules Are Cleared by Autophagy and Cdc48/VCP Function. Cell.

[229] Kedersha N, Panas MD, Achorn CA, Lyons S, Tisdale S, Hickman T (2016). G3BP-Caprin1-USP10 complexes mediate stress granule condensation and associate with 40S subunits. J Cell Biol.

[230] Kraft C, Peter M (2008). Is the Rsp5 ubiquitin ligase involved in the regulation of ribophagy?. Autophagy.

[231] Gupta R, Kus B, Fladd C, Wasmuth J, Tonikian R, Sidhu S (2007). Ubiquitination screen using protein microarrays for comprehensive identification of Rsp5 substrates in yeast. Mol Syst Biol.

[232] Ossareh-Nazari B, Bonizec M, Cohen M, Dokudovskaya S, Delalande F, Schaeffer C (2010). Cdc48 and Ufd3, new partners of the ubiquitin protease Ubp3, are required for ribophagy. EMBO Rep.

[233] Baxter BK, Abeliovich H, Zhang X, Stirling AG, Burlingame AL, Goldfarb DS (2005). Atg19p ubiquitination and the cytoplasm to vacuole trafficking pathway in yeast. J Biol Chem.

[234] Wyant GA, Abu-Remaileh M, Frenkel EM, Laqtom NN, Dharamdasani V, Lewis CA (2018). NUFIP1 is a ribosome receptor for starvation-induced ribophagy. Science.

[235] An H, Harper JW (2018). Systematic analysis of ribophagy in human cells reveals bystander flux during selective autophagy. Nat Cell Biol.

[236] Waliullah TM, Yeasmin AM, Kaneko A, Koike N, Terasawa M, Totsuka T (2017). Rim15 and Sch9 kinases are involved in induction of autophagic degradation of ribosomes in budding yeast. Biosci Biotechnol Biochem.

[237] Urban J, Soulard A, Huber A, Lippman S, Mukhopadhyay D, Deloche O (2007). Sch9 is a major target of TORC1 in Saccharomyces cerevisiae. Mol Cell.

[238] Yorimitsu T, Zaman S, Broach JR, Klionsky DJ (2007). Protein kinase A and Sch9 cooperatively regulate induction of autophagy in Saccharomyces cerevisiae. Mol Biol Cell.

[239] Yang Z, Geng J, Yen WL, Wang K, Klionsky DJ (2010). Positive or negative roles of different cyclin-dependent kinase Pho85-cyclin complexes orchestrate induction of autophagy in Saccharomyces cerevisiae. Mol Cell.

[240] Suzuki K (2013). Selective autophagy in budding yeast. Cell Death Differ.

[241] Hillwig MS, Contento AL, Meyer A, Ebany D, Bassham DC, Macintosh GC (2011). RNS2, a conserved member of the RNase T2 family, is necessary for ribosomal RNA decay in plants. Proc Natl Acad Sci U S A.

[242] Nishida Y, Arakawa S, Fujitani K, Yamaguchi H, Mizuta T, Kanaseki T (2009). Discovery of Atg5/Atg7-independent alternative macroautophagy. Nature.

[243] Tsuboyama K, Koyama-Honda I, Sakamaki Y, Koike M, Morishita H, Mizushima N (2016). The ATG conjugation systems are important for degradation of the inner autophagosomal membrane. Science.

[244] Denton D, Kumar S (2018). Ribophagy: new receptor discovered. Cell Res.

[245] Bardoni B, Willemsen R, Weiler IJ, Schenck A, Severijnen LA, Hindelang C (2003). NUFIP1 (nuclear FMRP interacting protein 1) is a nucleocytoplasmic shuttling protein associated with active synaptoneurosomes. Exp Cell Res.

[246] Jin M, Klionsky DJ (2018). Finding a ribophagy receptor. Autophagy.

[247] Schrader M, Bonekamp NA, Islinger M (2012). Fission and proliferation of peroxisomes. Bba-Mol Basis Dis.

[248] Delille HK, Bonekamp NA, Schrader M (2006). Peroxisomes and disease-an overview. Int J Biomed Sci.

[249] Huybrechts SJ, Van Veldhoven PP, Brees C, Mannaerts GP, Los GV, Fransen M (2009). Peroxisome dynamics in cultured mammalian cells. Traffic.

[250] Okumoto K, Kametani Y, Fujiki Y (2011). Two Proteases, Trypsin Domain-containing 1 (Tysnd1) and Peroxisomal Lon Protease (PsLon), Cooperatively Regulate Fatty Acid beta-Oxidation in Peroxisomal Matrix. J Biol Chem.

[251] Yokota S, Oda T, Fahimi HD (2001). The role of 15-lipoxygenase in disruption of the peroxisomal membrane and in programmed degradation of peroxisomes in normal rat liver. J Histochem Cytochem.

[252] Yokota S (2003). Degradation of normal and proliferated peroxisomes in rat hepatocytes: Regulation of peroxisomes quantity in cells. Microsc Res Techniq.

[253] Sakai Y, Oku M, van der Klei IJ, Kiel JAKW (2006). Pexophagy: Autophagic degradation of peroxisomes. Bba-Mol Cell Res.

[254] Monastyrska I, Klionsky DJ (2006). Autophagy in organelle homeostasis: peroxisome turnover. Mol Aspects Med.

[255] Farre JC, Manjithaya R, Mathewson RD, Subramani S (2008). PpAtg30 tags peroxisomes for turnover by selective autophagy. Dev Cell.

[256] Motley AM, Nuttall JM, Hettema EH (2012). Atg36: the Saccharomyces cerevisiae receptor for pexophagy. Autophagy.

[257] Deosaran E, Larsen KB, Hua R, Sargent G, Wang Y, Kim S (2013). NBR1 acts as an autophagy receptor for peroxisomes. J Cell Sci.

[258] Yamashita S, Abe K, Tatemichi Y, Fujiki Y (2014). The membrane peroxin PEX3 induces peroxisome-ubiquitination-linked pexophagy. Autophagy.

[259] Rubio N, Verrax J, Dewaele M, Verfaillie T, Johansen T, Piette J (2014). p38(MAPK)-regulated induction of p62 and NBR1 after photodynamic therapy promotes autophagic clearance of ubiquitin aggregates and reduces reactive oxygen species levels by supporting Nrf2-antioxidant signaling. Free Radic Biol Med.

[260] Nazarko TY (2014). Atg37 regulates the assembly of the pexophagic receptor protein complex. Autophagy.

[261] Burnett SF, Farre JC, Nazarko TY, Subramani S (2015). Peroxisomal Pex3 activates selective autophagy of peroxisomes via interaction with the pexophagy receptor Atg30. J Biol Chem.

[262] Nazarko TY, Ozeki K, Till A, Ramakrishnan G, Lotfi P, Yan M (2014). Peroxisomal Atg37 binds Atg30 or palmitoyl-CoA to regulate phagophore formation during pexophagy. J Cell Biol.

[263] Zientara-Rytter K, Ozeki K, Nazarko TY, Subramani S (2018). Pex3 and Atg37 compete to regulate the interaction between the pexophagy receptor, Atg30, and the Hrr25 kinase. Autophagy.

[264] Sargent G, van Zutphen T, Shatseva T, Zhang L, Di Giovanni V, Bandsma R (2016). PEX2 is the E3 ubiquitin ligase required for pexophagy during starvation. J Cell Biol.

[265] Marcassa E, Kallinos A, Jardine J, Rusilowicz-Jones EV, Martinez A, Kuehl S (2018). Dual role of USP30 in controlling basal pexophagy and mitophagy. EMBO Rep.

[266] Riccio V, Demers N, Hua R, Vissa M, Cheng DT, Strilchuk AW (2019). Deubiquitinating enzyme USP30 maintains basal peroxisome abundance by regulating pexophagy. J Cell Biol.

[267] Riccio V, McQuibban GA, Kim PK (2019). USP30: protector of peroxisomes and mitochondria. Mol Cell Oncol.

[268] Liu X, Ma C, Subramani S (2012). Recent advances in peroxisomal matrix protein import. Curr Opin Cell Biol.

[269] Zientara-Rytter K, Subramani S (2016). Autophagic degradation of peroxisomes in mammals. Biochem Soc Trans.

[270] Nordgren M, Francisco T, Lismont C, Hennebel L, Brees C, Wang B (2015). Export-deficient monoubiquitinated PEX5 triggers peroxisome removal in SV40 large T antigen-transformed mouse embryonic fibroblasts. Autophagy.

[271] Zhang J, Tripathi DN, Jing J, Alexander A, Kim J, Powell RT (2015). ATM functions at the peroxisome to induce pexophagy in response to ROS. Nat Cell Biol.

[272] Law KB, Bronte-Tinkew D, Di Pietro E, Snowden A, Jones RO, Moser A (2017). The peroxisomal AAA ATPase complex prevents pexophagy and development of peroxisome biogenesis disorders. Autophagy.

[273] Hara-Kuge S, Fujiki Y (2008). The peroxin Pex14p is involved in LC3-dependent degradation of mammalian peroxisomes. Exp Cell Res.

[274] Roach PJ (2011). AMPK -> ULK1 -> autophagy. Mol Cell Biol.

[275] Kim J, Kundu M, Viollet B, Guan KL (2011). AMPK and mTOR regulate autophagy through direct phosphorylation of Ulk1. Nat Cell Biol.

[276] Russell RC, Tian Y, Yuan H, Park HW, Chang YY, Kim J (2013). ULK1 induces autophagy by phosphorylating Beclin-1 and activating VPS34 lipid kinase. Nat Cell Biol.

[277] Zhang J, Kim J, Alexander A, Cai S, Tripathi DN, Dere R (2013). A tuberous sclerosis complex signalling node at the peroxisome regulates mTORC1 and autophagy in response to ROS. Nat Cell Biol.

[278] Alexander A, Cai SL, Kim J, Nanez A, Sahin M, MacLean KH (2010). ATM signals to TSC2 in the cytoplasm to regulate mTORC1 in response to ROS. Proc Natl Acad Sci U S A.

[279] Iwata J, Ezaki J, Komatsu M, Yokota S, Ueno T, Tanida I (2006). Excess peroxisomes are degraded by autophagic machinery in mammals. J Biol Chem.

[280] Shibata M, Oikawa K, Yoshimoto K, Kondo M, Mano S, Yamada K (2013). Highly oxidized peroxisomes are selectively degraded via autophagy in Arabidopsis. Plant Cell.

[281] Kim J, Lee H, Lee HN, Kim SH, Shin KD, Chung T (2013). Autophagy-related proteins are required for degradation of peroxisomes in Arabidopsis hypocotyls during seedling growth. Plant Cell.

[282] Du H, Kim S, Hur YS, Lee MS, Lee SH, Cheon CI (2015). A Cytosolic Thioredoxin Acts as a Molecular Chaperone for Peroxisome Matrix Proteins as Well as Antioxidant in Peroxisome. Mol Cells.

[283] Wanders RJA, Waterham HR, Ferdinandusse S (2016). Metabolic Interplay between Peroxisomes and Other Subcellular Organelles Including Mitochondria and the Endoplasmic Reticulum. Front Cell Dev Biol.

[284] Cho DH, Kim YS, Jo DS, Choe SK, Jo EK (2018). Pexophagy: Molecular Mechanisms and Implications for Health and Diseases. Mol Cells.

[285] Lass A, Zimmermann R, Oberer M, Zechner R (2011). Lipolysis - a highly regulated multi-enzyme complex mediates the catabolism of cellular fat stores. Prog Lipid Res.

[286] Kimmel AR, Brasaemle DL, McAndrews-Hill M, Sztalryd C, Londos C (2010). Adoption of PERILIPIN as a unifying nomenclature for the mammalian PAT-family of intracellular lipid storage droplet proteins. J Lipid Res.

[287] Wang CW (2016). Lipid droplets, lipophagy, and beyond. Biochim Biophys Acta.

[288] Singh R, Kaushik S, Wang Y, Xiang Y, Novak I, Komatsu M (2009). Autophagy regulates lipid metabolism. Nature.

[289] Lam T, Harmancey R, Vasquez H, Gilbert B, Patel N, Hariharan V (2016). Reversal of intramyocellular lipid accumulation by lipophagy and a p62-mediated pathway. Cell Death Discov.

[290] Martinez-Lopez N, Singh R (2016). Telemetric control of peripheral lipophagy by hypothalamic autophagy. Autophagy.

[291] Kaushik S, Rodriguez-Navarro JA, Arias E, Kiffin R, Sahu S, Schwartz GJ (2011). Autophagy in hypothalamic AgRP neurons regulates food intake and energy balance. Cell Metab.

[292] Martinez-Vicente M, Talloczy Z, Wong E, Tang G, Koga H, Kaushik S (2010). Cargo recognition failure is responsible for inefficient autophagy in Huntington's disease. Nat Neurosci.

[293] Xu X, Grijalva A, Skowronski A, van Eijk M, Serlie MJ, Ferrante AW Jr (2013). Obesity activates a program of lysosomal-dependent lipid metabolism in adipose tissue macrophages independently of classic activation. Cell Metab.

[294] Khaldoun SA, Emond-Boisjoly MA, Chateau D, Carriere V, Lacasa M, Rousset M (2014). Autophagosomes contribute to intracellular lipid distribution in enterocytes. Mol Biol Cell.

[295] Schroeder B, Schulze RJ, Weller SG, Sletten AC, Casey CA, McNiven MA (2015). The small GTPase Rab7 as a central regulator of hepatocellular lipophagy. Hepatology.

[296] Schulze RJ, McNiven MA (2014). A well-oiled machine: DNM2/dynamin 2 helps keep hepatocyte lipophagy running smoothly. Autophagy.

[297] Zechner R, Madeo F, Kratky D (2017). Cytosolic lipolysis and lipophagy: two sides of the same coin. Nat Rev Mol Cell Biol.

[298] Schreiber R, Xie H, Schweiger M (2019). Of mice and men: The physiological role of adipose triglyceride lipase (ATGL). Biochim Biophys Acta Mol Cell Biol Lipids.

[299] Martinez-Lopez N, Garcia-Macia M, Sahu S, Athonvarangkul D, Liebling E, Merlo P (2016). Autophagy in the CNS and Periphery Coordinate Lipophagy and Lipolysis in the Brown Adipose Tissue and Liver. Cell Metab.

[300] Sathyanarayan A, Mashek MT, Mashek DG (2017). ATGL Promotes Autophagy/Lipophagy via SIRT1 to Control Hepatic Lipid Droplet Catabolism. Cell Rep.

[301] Kim KY, Jang HJ, Yang YR, Park KI, Seo J, Shin IW (2016). SREBP-2/PNPLA8 axis improves non-alcoholic fatty liver disease through activation of autophagy. Sci Rep.

[302] Negoita F, Blomdahl J, Wasserstrom S, Winberg ME, Osmark P, Larsson S (2019). PNPLA3 variant M148 causes resistance to starvation-mediated lipid droplet autophagy in human hepatocytes. J Cell Biochem.

[303] Dupont N, Chauhan S, Arko-Mensah J, Castillo EF, Masedunskas A, Weigert R (2014). Neutral lipid stores and lipase PNPLA5 contribute to autophagosome biogenesis. Curr Biol.

[304] Shpilka T, Welter E, Borovsky N, Amar N, Mari M, Reggiori F (2015). Lipid droplets and their component triglycerides and steryl esters regulate autophagosome biogenesis. EMBO J.

[305] Ward C, Martinez-Lopez N, Otten EG, Carroll B, Maetzel D, Singh R (2016). Autophagy, lipophagy and lysosomal lipid storage disorders. Biochim Biophys Acta.

[306] Varshney R, Varshney R, Mishra R, Gupta S, Sircar D, Roy P (2018). Kaempferol alleviates palmitic acid-induced lipid stores, endoplasmic reticulum stress and pancreatic beta-cell dysfunction through AMPK/mTOR-mediated lipophagy. J Nutr Biochem.

[307] Kurahashi T, Hamashima S, Shirato T, Lee J, Homma T, Kang ES (2015). An SOD1 deficiency enhances lipid droplet accumulation in the fasted mouse liver by aborting lipophagy. Biochem Biophys Res Commun.

[308] Xiong J, Wang K, He J, Zhang G, Zhang D, Chen F (2016). TFE3 Alleviates Hepatic Steatosis through Autophagy-Induced Lipophagy and PGC1alpha-Mediated Fatty Acid beta-Oxidation. Int J Mol Sci.

[309] Settembre C, De Cegli R, Mansueto G, Saha PK, Vetrini F, Visvikis O (2013). TFEB controls cellular lipid metabolism through a starvation-induced autoregulatory loop. Nat Cell Biol.

[310] Settembre C, Di Malta C, Polito VA, Garcia Arencibia M, Vetrini F, Erdin S (2011). TFEB links autophagy to lysosomal biogenesis. Science.

[311] Napolitano G, Ballabio A (2016). TFEB at a glance. J Cell Sci.

[312] O'Rourke EJ, Ruvkun G (2013). MXL-3 and HLH-30 transcriptionally link lipolysis and autophagy to nutrient availability. Nat Cell Biol.

[313] Zhu L, Yuan Y, Yuan L, Li L, Liu F, Liu J (2020). Activation of TFEB-mediated autophagy by trehalose attenuates mitochondrial dysfunction in cisplatin-induced acute kidney injury. Theranostics.

[314] Tatsumi T, Takayama K, Ishii S, Yamamoto A, Hara T, Minami N (2018). Forced lipophagy reveals that lipid droplets are required for early embryonic development in mouse. Development.

[315] Lee JM, Wagner M, Xiao R, Kim KH, Feng D, Lazar MA (2014). Nutrient-sensing nuclear receptors coordinate autophagy. Nature.

[316] Liu K, Czaja MJ (2013). Regulation of lipid stores and metabolism by lipophagy. Cell Death Differ.

[317] Zhu X, Xiong T, Liu P, Guo X, Xiao L, Zhou F (2018). Quercetin ameliorates HFD-induced NAFLD by promoting hepatic VLDL assembly and lipophagy via the IRE1a/XBP1s pathway. Food Chem Toxicol.

[318] Ding WX, Li M, Yin XM (2011). Selective taste of ethanol-induced autophagy for mitochondria and lipid droplets. Autophagy.

[319] Narabayashi K, Ito Y, Eid N, Maemura K, Inoue T, Takeuchi T (2015). Indomethacin suppresses LAMP-2 expression and induces lipophagy and lipoapoptosis in rat enterocytes via the ER stress pathway. J Gastroenterol.

[320] Czaja MJ (2016). Function of Autophagy in Nonalcoholic Fatty Liver Disease. Dig Dis Sci.

[321] Zhang J, Lan Y, Li MY, Lamers MM, Fusade-Boyer M, Klemm E (2018). Flaviviruses Exploit the Lipid Droplet Protein AUP1 to Trigger Lipophagy and Drive Virus Production. Cell Host Microbe.

[322] Jung WH, Liu CC, Yu YL, Chang YC, Lien WY, Chao HC (2017). Lipophagy prevents activity-dependent neurodegeneration due to dihydroceramide accumulation *in vivo*. EMBO Rep.

[323] Gomez de Cedron M, Ramirez de Molina A (2016). Microtargeting cancer metabolism: opening new therapeutic windows based on lipid metabolism. J Lipid Res.

[324] Zhao T, Du H, Ding X, Walls K, Yan C (2015). Activation of mTOR pathway in myeloid-derived suppressor cells stimulates cancer cell proliferation and metastasis in lal(-/-) mice. Oncogene.

[325] Kounakis K, Chaniotakis M, Markaki M, Tavernarakis N (2019). Emerging Roles of Lipophagy in Health and Disease. Front Cell Dev Biol.

[326] Rui YN, Xu Z, Patel B, Chen Z, Chen D, Tito A (2015). Huntingtin functions as a scaffold for selective macroautophagy. Nat Cell Biol.

[327] Martinez-Vicente M, Cuervo AM (2007). Autophagy and neurodegeneration: when the cleaning crew goes on strike. Lancet Neurol.

[328] Saftig P, Klumperman J (2009). Lysosome biogenesis and lysosomal membrane proteins: trafficking meets function. Nat Rev Mol Cell Bio.

[329] Aits S, Jaattela M (2013). Lysosomal cell death at a glance. J Cell Sci.

[330] Boya P, Kroemer G (2008). Lysosomal membrane permeabilization in cell death. Oncogene.

[331] Maejima I, Takahashi A, Omori H, Kimura T, Takabatake Y, Saitoh T (2013). Autophagy sequesters damaged lysosomes to control lysosomal biogenesis and kidney injury. Embo Journal.

[332] Hung YH, Chen LMW, Yang JY, Yang WY (2013). Spatiotemporally controlled induction of autophagy-mediated lysosome turnover. Nat Commun.

[333] Hasegawa J, Maejima I, Iwamoto R, Yoshimori T (2015). Selective autophagy: Lysophagy. Methods.

[334] Dehay B, Bove J, Rodriguez-Muela N, Perier C, Recasens A, Boya P (2010). Pathogenic Lysosomal Depletion in Parkinson's Disease. J Neurosci.

[335] Mizushima N (2019). The ubiquitin E2 enzyme UBE2QL1 mediates lysophagy. EMBO Rep.

[336] Yoshida Y, Yasuda S, Fujita T, Hamasaki M, Murakami A, Kawawaki J (2017). Ubiquitination of exposed glycoproteins by SCF(FBXO27) directs damaged lysosomes for autophagy. Proc Natl Acad Sci U S A.

[337] Huett A, Heath RJ, Begun J, Sassi SO, Baxt LA, Vyas JM (2012). The LRR and RING Domain Protein LRSAM1 Is an E3 Ligase Crucial for Ubiquitin-Dependent Autophagy of Intracellular Salmonella Typhimurium. Cell Host Microbe.

[338] Houzelstein D, Goncalves IR, Fadden AJ, Sidhu SS, Cooper DNW, Drickamer K (2004). Phylogenetic analysis of the vertebrate galectin family. Mol Biol Evol.

[339] Paz I, Sachse M, Dupont N, Mounier J, Cederfur C, Enninga J (2010). Galectin-3, a marker for vacuole lysis by invasive pathogens. Cell Microbiol.

[340] Thurston TLM, Wandel MP, von Muhlinen N, Foeglein A, Randow F (2012). Galectin 8 targets damaged vesicles for autophagy to defend cells against bacterial invasion. Nature.

[341] Chauhan S, Kumar S, Jain A, Ponpuak M, Mudd MH, Kimura T (2016). TRIMs and Galectins Globally Cooperate and TRIM16 and Galectin-3 Co-direct Autophagy in Endomembrane Damage Homeostasis. Dev Cell.

[342] Fujita N, Morita E, Itoh T, Tanaka A, Nakaoka M, Osada Y (2013). Recruitment of the autophagic machinery to endosomes during infection is mediated by ubiquitin. J Cell Biol.

[343] Fiskin E, Bionda T, Dikic I, Behrends C (2016). Global Analysis of Host and Bacterial Ubiquitinome in Response to Salmonella Typhimurium Infection. Mol Cell.

[344] Yoshida Y, Yasuda S, Fujita T, Hamasaki M, Murakami A, Kawawaki J (2017). Ubiquitination of exposed glycoproteins by SCFFBXO27 directs damaged lysosomes for autophagy. P Natl Acad Sci USA.

[345] Papadopoulos C, Kirchner P, Bug M, Grum D, Koerver L, Schulze N (2017). VCP/p97 cooperates with YOD1, UBXD1 and PLAA to drive clearance of ruptured lysosomes by autophagy. EMBO J.

[346] Seczynska M, Dikic I (2017). Removing the waste bags: how p97 drives autophagy of lysosomes. EMBO J.

[347] Ferreira CR, Gahl WA (2017). Lysosomal storage diseases. Transl Sci Rare Dis.

[348] Marques ARA, Saftig P (2019). Lysosomal storage disorders-challenges, concepts and avenues for therapy: beyond rare diseases. J Cell Sci.

[349] Rigante D, Cipolla C, Basile U, Gulli F, Savastano MC (2017). Overview of immune abnormalities in lysosomal storage disorders. Immunol Lett.

[350] Goldstein JL, Brown MS (2015). A century of cholesterol and coronaries: from plaques to genes to statins. Cell.

[351] Sergin I, Evans TD, Zhang XY, Bhattacharya S, Stokes CJ, Song E (2017). Exploiting macrophage autophagy-lysosomal biogenesis as a therapy for atherosclerosis. Nat Commun.

[352] Evans TD, Jeong SJ, Zhang X, Sergin I, Razani B (2018). TFEB and trehalose drive the macrophage autophagy-lysosome system to protect against atherosclerosis. Autophagy.

[353] Sambri I, D'Alessio R, Ezhova Y, Giuliano T, Sorrentino NC, Cacace V (2017). Lysosomal dysfunction disrupts presynaptic maintenance and restoration of presynaptic function prevents neurodegeneration in lysosomal storage diseases. EMBO Mol Med.

[354] Beck M (2018). Treatment strategies for lysosomal storage disorders. Dev Med Child Neurol.

[355] Platt FM, d'Azzo A, Davidson BL, Neufeld EF, Tifft CJ (2018). Lysosomal storage diseases. Nat Rev Dis Primers.

[356] Platt FM (2018). Emptying the stores: lysosomal diseases and therapeutic strategies. Nat Rev Drug Discov.

[357] Park YE, Hayashi YK, Bonne G, Arimura T, Noguchi S, Nonaka I (2009). Autophagic degradation of nuclear components in mammalian cells. Autophagy.

[358] Huang HH, Kawamata T, Horie T, Tsugawa H, Nakayama Y, Ohsumi Y (2015). Bulk RNA degradation by nitrogen starvation-induced autophagy in yeast. EMBO J.

[359] Kvam E, Goldfarb DS (2007). Nucleus-vacuole junctions and piecemeal microautophagy of the nucleus in S. cerevisiae. Autophagy.

[360] Millen JI, Krick R, Prick T, Thumm M, Goldfarb DS (2009). Measuring piecemeal microautophagy of the nucleus in Saccharomyces cerevisiae. Autophagy.

[361] Roberts P, Moshitch-Moshkovitz S, Kvam E, O'Toole E, Winey M, Goldfarb DS (2003). Piecemeal microautophagy of nucleus in Saccharomyces cerevisiae. Mol Biol Cell.

[362] Mijaljica D, Prescott M, Devenish RJ (2012). A Late Form of Nucleophagy in Saccharomyces cerevisiae. Plos One.

[363] Lee IH, Kawai Y, Fergusson MM, Rovira II, Bishop AJR, Motoyama N (2012). Atg7 Modulates p53 Activity to Regulate Cell Cycle and Survival During Metabolic Stress. Science.

[364] Simon HU, Yousefi S, Schmid I, Friis R (2014). ATG5 can regulate p53 expression and activation. Cell Death Dis.

[365] Lu JH, He LQ, Behrends C, Araki M, Araki K, Wang QJ (2014). NRBF2 regulates autophagy and prevents liver injury by modulating Atg14L-linked phosphatidylinositol-3 kinase III activity. Nat Commun.

[366] Huang R, Xu YF, Wan W, Shou X, Qian JL, You ZY (2015). Deacetylation of Nuclear LC3 Drives Autophagy Initiation under Starvation. Mol Cell.

[367] Xu F, Fang YX, Yan LL, Xu L, Zhang SP, Cao Y (2017). Nuclear localization of Beclin 1 promotes radiation-induced DNA damage repair independent of autophagy. Sci Rep.

[368] Isakson P, Holland P, Simonsen A (2013). The role of ALFY in selective autophagy. Cell Death Differ.

[369] Shimi T, Pfleghaar K, Kojima S, Pack CG, Solovei I, Goldman AE (2008). The A- and B-type nuclear lamin networks: microdomains involved in chromatin organization and transcription. Genes Dev.

[370] Shimi T, Butin-Israeli V, Adam SA, Hamanaka RB, Goldman AE, Lucas CA (2011). The role of nuclear lamin B1 in cell proliferation and senescence. Genes Dev.

[371] Ivanov A, Pawlikowski J, Manoharan I, van Tuyn J, Nelson DM, Rai TS (2013). Lysosome-mediated processing of chromatin in senescence. J Cell Biol.

[372] Dou ZX, Xu CY, Donahue G, Shimi T, Pan JA, Zhu JJ (2015). Autophagy mediates degradation of nuclear lamina. Nature.

[373] Dou ZX, Ivanov A, Adams PD, Berger SL (2016). Mammalian autophagy degrades nuclear constituents in response to tumorigenic stress. Autophagy.

[374] Rello-Varona S, Lissa D, Shen SS, Niso-Santano M, Senovilla L, Marino G (2012). Autophagic removal of micronuclei. Cell Cycle.

[375] Akinduro O, Sully K, Patel A, Robinson DJ, Chikh A, McPhail G (2016). Constitutive Autophagy and Nucleophagy during Epidermal Differentiation. J Invest Dermatol.

[376] Baron O, Boudi A, Dias C, Schilling M, Nolle A, Vizcay-Barrena G (2017). Stall in Canonical Autophagy-Lysosome Pathways Prompts Nucleophagy-Based Nuclear Breakdown in Neurodegeneration. Curr Biol.

[377] Strzyz P (2015). Nuclear autophagy in tumour suppression. Nat Rev Mol Cell Bio.

[378] Sharma V, Verma S, Seranova E, Sarkar S, Kumar D (2018). Selective Autophagy and Xenophagy in Infection and Disease. Front Cell Dev Biol.

[379] Gomes LC, Dikic I (2014). Autophagy in antimicrobial immunity. Mol Cell.

[380] Whang MI, Tavares RM, Benjamin DI, Kattah MG, Advincula R, Nomura DK (2017). The Ubiquitin Binding Protein TAX1BP1 Mediates Autophagasome Induction and the Metabolic Transition of Activated T Cells. Immunity.

[381] Yang Q, Liu TT, Lin H, Zhang M, Wei J, Luo WW (2017). TRIM32-TAX1BP1-dependent selective autophagic degradation of TRIF negatively regulates TLR3/4-mediated innate immune responses. PLoS Pathog.

[382] Sagnier S, Daussy CF, Borel S, Robert-Hebmann V, Faure M, Blanchet FP (2015). Autophagy restricts HIV-1 infection by selectively degrading Tat in CD4^+^ T lymphocytes. J Virol.

[383] Nardacci R, Amendola A, Ciccosanti F, Corazzari M, Esposito V, Vlassi C (2014). Autophagy plays an important role in the containment of HIV-1 in nonprogressor-infected patients. Autophagy.

[384] Sparrer KMJ, Gableske S, Zurenski MA, Parker ZM, Full F, Baumgart GJ (2017). TRIM23 mediates virus-induced autophagy via activation of TBK1. Nat Microbiol.

[385] Wiersinga WJ, Rhodes A, Cheng AC, Peacock SJ, Prescott HC (2020). Pathophysiology, Transmission, Diagnosis, and Treatment of Coronavirus Disease 2019 (COVID-19): A Review. JAMA.

[386] Satoo K, Noda NN, Kumeta H, Fujioka Y, Mizushima N, Ohsumi Y (2009). The structure of Atg4B-LC3 complex reveals the mechanism of LC3 processing and delipidation during autophagy. EMBO J.

[387] Yamaguchi M, Noda NN, Nakatogawa H, Kumeta H, Ohsumi Y, Inagaki F (2010). Autophagy-related protein 8 (Atg8) family interacting motif in Atg3 mediates the Atg3-Atg8 interaction and is crucial for the cytoplasm-to-vacuole targeting pathway. J Biol Chem.

[388] Gao C, Cao W, Bao L, Zuo W, Xie G, Cai T (2010). Autophagy negatively regulates Wnt signalling by promoting Dishevelled degradation. Nat Cell Biol.

[389] Pankiv S, Alemu EA, Brech A, Bruun JA, Lamark T, Overvatn A (2010). FYCO1 is a Rab7 effector that binds to LC3 and PI3P to mediate microtubule plus end-directed vesicle transport. J Cell Biol.

[390] Yorimitsu T, Klionsky DJ (2005). Atg11 links cargo to the vesicle-forming machinery in the cytoplasm to vacuole targeting pathway. Mol Biol Cell.

[391] Suzuki K, Kondo C, Morimoto M, Ohsumi Y (2010). Selective transport of alpha-mannosidase by autophagic pathways: identification of a novel receptor, Atg34p. J Biol Chem.

[392] Torggler R, Papinski D, Kraft C (2017). Assays to Monitor Autophagy in Saccharomyces cerevisiae. Cells.

[393] Orhon I, Reggiori F (2017). Assays to Monitor Autophagy Progression in Cell Cultures. Cells.

[394] Li W, Li S, Li Y, Lin X, Hu Y, Meng T (2019). Immunofluorescence Staining Protocols for Major Autophagy Proteins Including LC3, P62, and ULK1 in Mammalian Cells in Response to Normoxia and Hypoxia. Methods Mol Biol.

[395] Anding AL, Baehrecke EH (2017). Cleaning House: Selective Autophagy of Organelles. Dev Cell.

[396] Zheng K, He Z, Kitazato K, Wang Y (2019). Selective Autophagy Regulates Cell Cycle in Cancer Therapy. Theranostics.

